# Design of a metabolically-stable peptide therapeutic with triple-hormone-receptor agonist activity

**DOI:** 10.1007/s10822-026-00903-5

**Published:** 2026-07-30

**Authors:** Shubham Vishnoi, Sarah Hudson, Shayon Bhattacharya, Damien Thompson

**Affiliations:** 1 Department of Physics, SSPC the Research Ireland Centre for Pharmaceuticals, Bernal Institute, University of Limerick, Limerick, V94 T9PX Ireland; 2 Department of Chemical Sciences, SSPC the Research Ireland Centre for Pharmaceuticals, Bernal Institute, University of Limerick, Limerick, V94 T9PX Ireland; 3 Department of Biological Sciences, SSPC the Research Ireland Centre for Pharmaceuticals, Bernal Institute, University of Limerick, Limerick, V94 T9PX Ireland; 4 Health Research Institute, University of Limerick, Limerick, V94 T9PX Ireland

**Keywords:** Peptide-based therapeutics, Diabetes, G protein-coupled receptor (GPCR) modelling, Peptide drug design, Triple agonist, GLP-1, Polypharmacy

## Abstract

Peptide-based therapeutics such as tirzepatide and semaglutide target the neuroendocrine system to remediate both diabetes and obesity. In addition to integrating the complementary actions of two endogenous metabolically related hormones targeting glucagon-like peptide-1 and glucagon receptors (GLP-1R and GCGR), the synergistic targeting of a third G protein-coupled receptor (GPCR), namely the gastric inhibitory polypeptide receptor (GIPR), may achieve optimal control of blood glucose levels and weight loss in patients with diabetes and obesity. In this work, we harness the predictive power of sequence-derived in silico mutagenesis to rationally design effective single-molecule peptides with triple-receptor-agonist activities. We model the binding profiles of our designed peptides to the GIP, GLP-1 and GCG receptor triad through multiple, long, repeat molecular simulations and effective binding enthalpy calculations to assess their predicted multi-receptor engagement profiles. Our data predict a balanced, highly favourable effective binding enthalpy profile of our newly designed peptides for the three receptors compared to endogenous peptides and the experimental reference peptides, with high targeted specificity for the receptors. The peptides are further engineered to structurally support metabolic stability by substituting sites prone to proteolytic cleavage. The predicted triple-hormone-receptor agonist peptide mechanism opens new avenues for the synthesis and activity testing of multi-targeting peptides with improved stability, offering a computational design strategy for testing the potential reduction of adverse gastrointestinal effects of peptide therapeutics and support future development of lower-dose oral formulations.

**Supplementary Information:**

The online version contains supplementary material available at 10.1007/s10822-026-00903-5.

## Introduction

Metabolic syndrome affects an estimated one in three American adults and around one in four globally, creating increased risk of cardiovascular diseases and diabetes through a cluster of co-occurring conditions, including obesity, high cholesterol, and insulin resistance [1, 2]. One of the most prevalent metabolic diseases is type 2 diabetes mellitus (T2DM), which accounts for approximately 90% of all cases of diabetes [3]. Obesity is the leading risk factor for T2DM as it negatively impacts lipid metabolism, insulin sensitivity, and pancreatic β-cell functioning [4]. The sharp rise in obesity and associated comorbidities in the last 30 years motivates the development of effective pharmacotherapy to treat metabolic disorders [5]. G protein-coupled receptors (GPCRs) continue to be the primary targets for the treatment of T2DM [6].

Several drugs for the treatment of metabolic disorders, such as diabetes and obesity, have been developed based on the incretins, gut-derived hormones, including glucagon-like peptide-1 (GLP-1) and gastric inhibitory polypeptide (GIP), secreted by L and K cells of the intestine, respectively. These hormones stimulate insulin secretion [7], modulate hepatic glycogen and fat content, and control appetite and blood glucose levels [8]. GLP-1 is a potent regulator of blood glucose, primarily by stimulating insulin secretion, inhibiting glucagon secretion, slowing gastric emptying, and promoting satiety. Additionally, GLP-1 supports pancreatic β-cell proliferation and survival, which may enhance insulin production over time [9]. In contrast, GIP also stimulates insulin secretion in a glucose-dependent manner but is particularly involved in lipid metabolism, promoting fatty acid uptake and triglyceride storage in adipose tissue [10, 11]. Pancreatic α-cells produce the glucagon (GCG) hormone that stimulates the production of glucose from non-carbohydrate substrates in the liver (hepatocytic gluconeogenesis), thereby increasing blood glucose levels [12].

The newly developed peptide-based drug tirzepatide, a dual agonist targeting the GLP-1 receptor (GLP-1R) and GIPR, has shown promising clinical outcomes in lowering blood glucose and reducing body weight in individuals with obesity [13]. However, severe gastrointestinal adverse events have been reported with continued use of tirzepatide [14]. To address this, polyagonist therapeutics that simultaneously activate multiple receptors are hypothesised to offer more balanced glycemic and metabolic regulation pathways, presenting a distinct molecular strategy compared to currently available mono- and dual-receptor agonists [15]. More recently, the triple-hormone-receptor agonist retatrutide (LY3437943) has advanced through pivotal Phase 3 clinical trials, highlighting the therapeutic potential of simultaneous tri-receptor activation for metabolic disease [16].

**Fig. 1:**
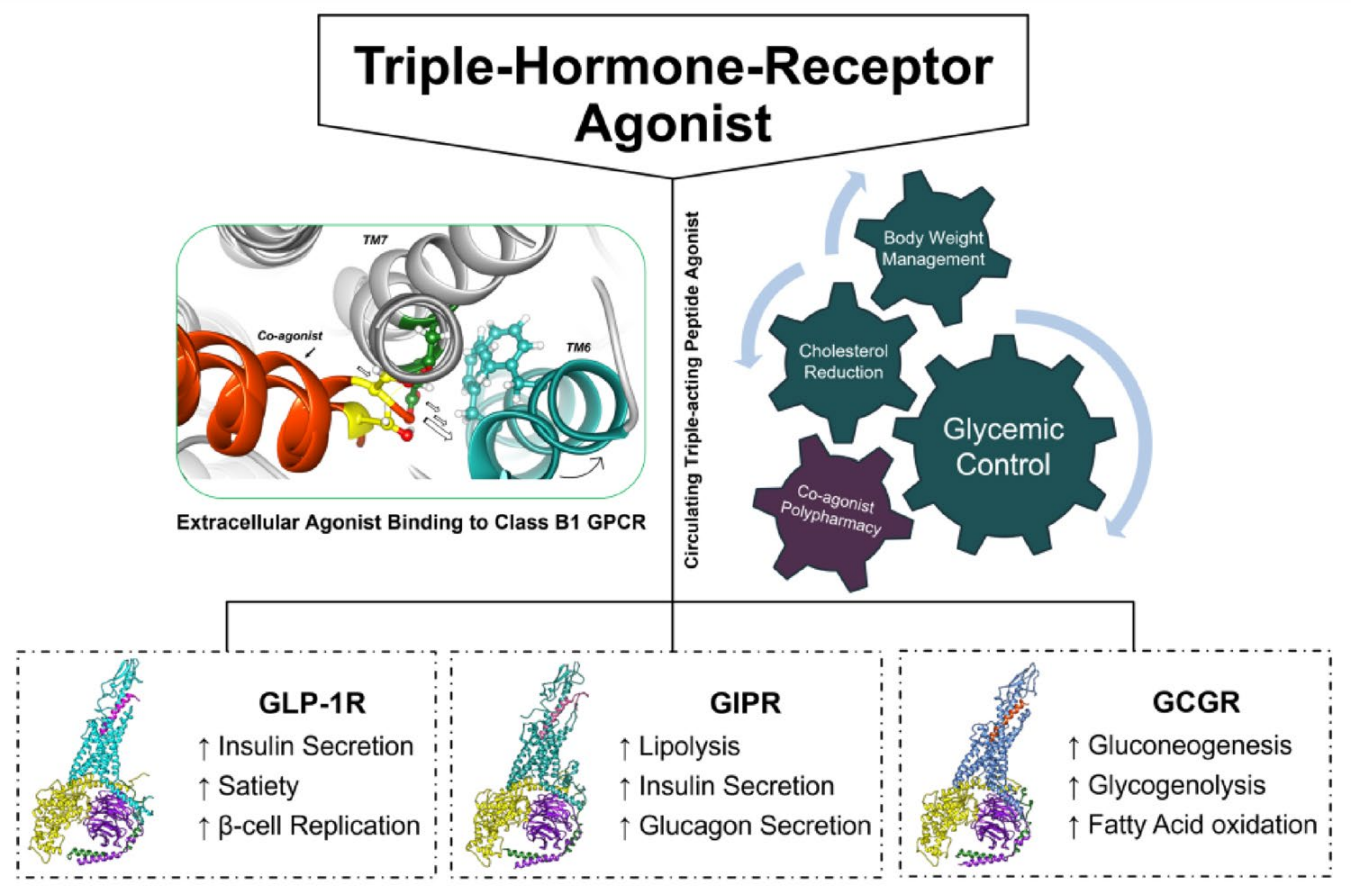
Strategy for targeting class B1 GPCR signalling using a polypharmacy approach. An effective triple-hormone-receptor agonist must target Glucagon-like Peptide-1 Receptor (GLP-1R), Glucagon Receptor (GCGR) and Gastric Inhibitory Polypeptide Receptor (GIPR), and is a promising avenue to restore regulation of glucose and lipid metabolism simultaneously by enhancing insulin secretion, promoting lipid oxidation, gluconeogenesis (production of glucose from non-carbohydrate sources) and glycogenolysis (breakdown of glycogen to glucose)

Here, we explore the synergistic targeting of three GPCRs—GCGR, GIPR, and GLP-1R, by bioactive peptides (Fig. 1). We propose a novel, rationally engineered unimolecular triple-hormone-receptor agonist peptide framework, advancing on in silico design rules that we recently developed for a dual-agonist peptide targeting GCGR and GLP-1R with strong predicted binding affinity [17]. The triple agonist peptide designed in this work is intended to function as a hybrid that mimics the interactions of three endogenous peptide hormones—GCG (29 residues), GLP-1 (31 residues), and GIP (42 residues), with their respective cognate receptors GCGR, GLP-1R, and GIPR.

The balanced, controlled activation of these three GPCRs targeted simultaneously could serve as a key pharmacotherapeutic strategy to enhance insulin secretion and improve glucose homeostasis [18]. Given the overlapping roles of GLP-1, GIP, and GCG, the rational combination of their engineered analogues may yield synergistic therapeutic benefits in managing diabetes and related metabolic diseases. This approach supports the development of unimolecular polypharmacy drugs and potentially enhances patient adherence and clinical outcomes (Fig. 1) [19].

Here, we utilise sequence-derived in silico mutagenesis to rationally design a new peptide therapeutic and investigate its ability to simultaneously activate GIPR, GLP-1R and GCGR as a synergistic triple agonist (Table S1). To optimise receptor targeting, we consider both specificity (primarily binding to the intended receptor) and selectivity (preferential binding among related receptors). We predict the effective binding enthalpy, specificity, and selectivity of our designed peptide via molecular dynamics simulations, assessing the feasibility of triple-hormone-receptor agonism. While assessing absolute binding affinities remains a computational challenge [20], molecular dynamics-based end-state methods, such as MM/PBSA [21], have emerged as robust tools for navigating the exponentially expanding peptide sequence space [22–24]. These approaches could enable precise control of inter- and intramolecular interactions [17], providing the thermodynamic insights necessary to rationally design functional triple-agonists that surpass the limitations of experience-based or purely experimental exploration.

The novel peptide scaffold is generated from a combination of endogenous peptide sequences and rational, template-based design rules to establish a structural model for multi-receptor targeting. We then explore the atomic-level conformational dynamics and receptor activation and signalling by fully mapping extracellular peptide binding in the membrane environment, including intracellular Gs protein coupling (which initiates downstream signalling), using long-timescale molecular dynamics (MD) simulations. This is complemented by residue-level interaction mapping and effective binding enthalpies calculations. Our data identify a rationally engineered triple-hormone-receptor agonist peptide with improved predicted balanced effective binding enthalpies and selectivity over both endogenous peptides and experimental drug candidates.

## Results

### Designed triple-hormone-receptor agonist peptide shows balanced predicted effective binding enthalpy profiles to GCGR, GLP-1R and GIPR

**Fig. 2:**
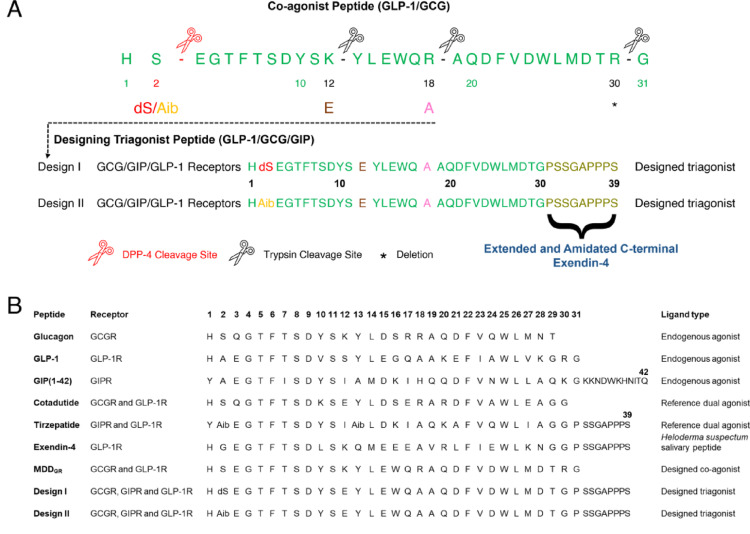
Modelled peptide sequences and design rules. **A** The modelled triple-hormone-receptor agonist sequence design rules, including mutations to impede proteolytic cleavage by dipeptidyl peptidase 4 (DPP-4) and trypsin enzymes. The nonstandard amino acid residue at position 2 is α-amino isobutyric acid (Aib) or dextroserine (dS). **B** Amino acid sequences of all modelled peptides, including Design I and Design II

**Fig. 3:**
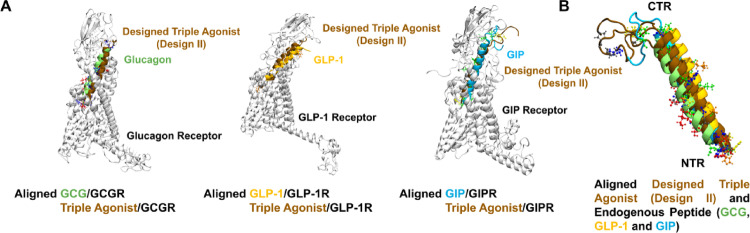
Structural insights into the binding of the designed triple agonist peptide with three class B1 GPCRs: GCGR, GLP-1R and GIPR. The figure highlights the binding modes of the designed triple-hormone-receptor agonist Design II, compared with the native endogenous ligands—glucagon, GLP-1, and GIP, for their respective receptors, represented as superimposed 3D coordinates. The designed peptide shows high effective binding enthalpies for GCGR, GLP-1R, and GIPR, adopting distinct, stable binding poses in each receptor. **A** Comparisons of receptor conformations and binding pockets stabilised by the receptor and the triple agonist peptide, showing superimposition of the receptor–ligand complexes from the receptor:Gs complex structures with native endogenous ligands and the triple agonist peptide. **B** Superimposition of the peptide ligands (glucagon, GLP-1 and GIP and triple agonist peptide) from the three receptors:Gs complex structures with native endogenous ligands and Design II peptide in complex with the three receptors:Gs complex structures, reveals the structural overlap in the conformations. The N terminus of the designed triple agonist forms an extensive hydrogen bond network with polar residues (depicted using CPK representation and coloured by residue name) deep in the transmembrane (TM) binding cavity in the receptors, similar to the interactions observed with their endogenous ligands in the complex

**Fig. 4:**
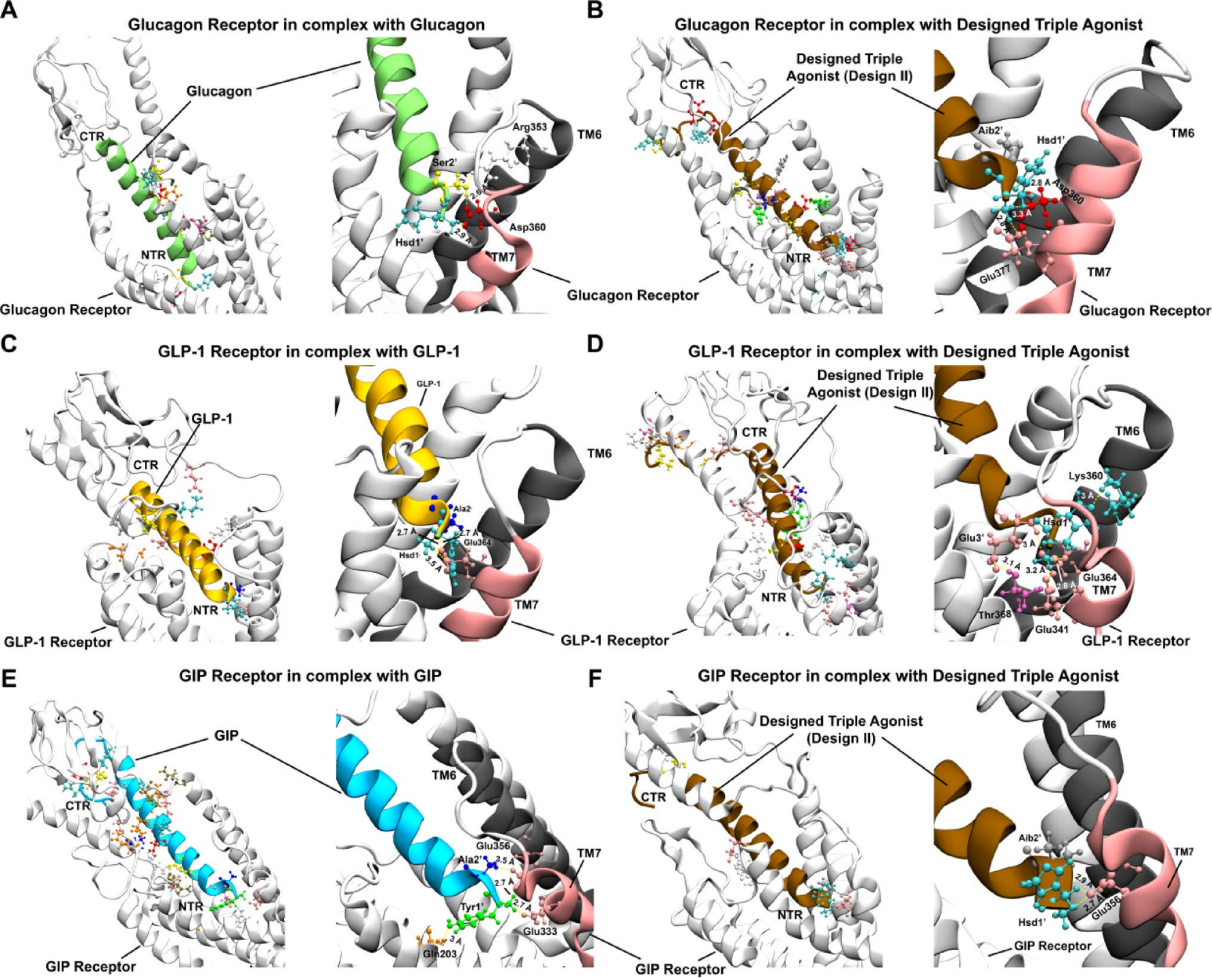
Detailed binding cavity interactions across the three receptors. **A** and **B** Interactions of glucagon and triple agonist peptide within the GCGR TM Binding Cavity. In GCGR, the dynamics reveal critical hydrogen-bond interactions between the triple agonist peptide and the receptor (His1’-Asp360 and Aib2’-Asp360) analogous to those in the GCGR:GCG complex (His1’-Asp360 and Ser2’-Asp360). **C** and **D** Interactions of GLP-1 and triple agonist peptide within the GLP-1R TM Binding Cavity. In GLP-1R, the dynamics reveal critical hydrogen bond interactions between the triple agonist peptide and the receptor (His1’-Glu364 and Glu3’-Glu364) mirroring those in the GLP-1:GLP-1R complex (His1’-Glu364 and Ala2’-Glu364). The hydrogen bond between Glu3’-Glu364 in the triple agonist:GLP-1R complex compensates for native contact Ala2’-Glu364 in GLP-1:GLP-1R complex, while the designed triple agonist also forms an additional His1’-Lys360 hydrogen bond. **E** and **F** Interactions of GIP and triple agonist peptide within the GIPR TM Binding Cavity. In GIPR, the dynamics reveal critical hydrogen bond interactions between the triple agonist peptide and the receptor (His1’-Glu356 and Aib2’-Glu356) mimicking those in the GIPR:GIP complex (Tyr1’-Glu333 and Ala2’-Glu356). Such extracellular binding interactions and structural shifts are key in promoting signal transduction and receptor activation across all three GPCRs, illustrating the versatility of the designed triple agonist in mimicking native ligand interactions to trigger downstream signalling mechanisms

**Fig. 5:**
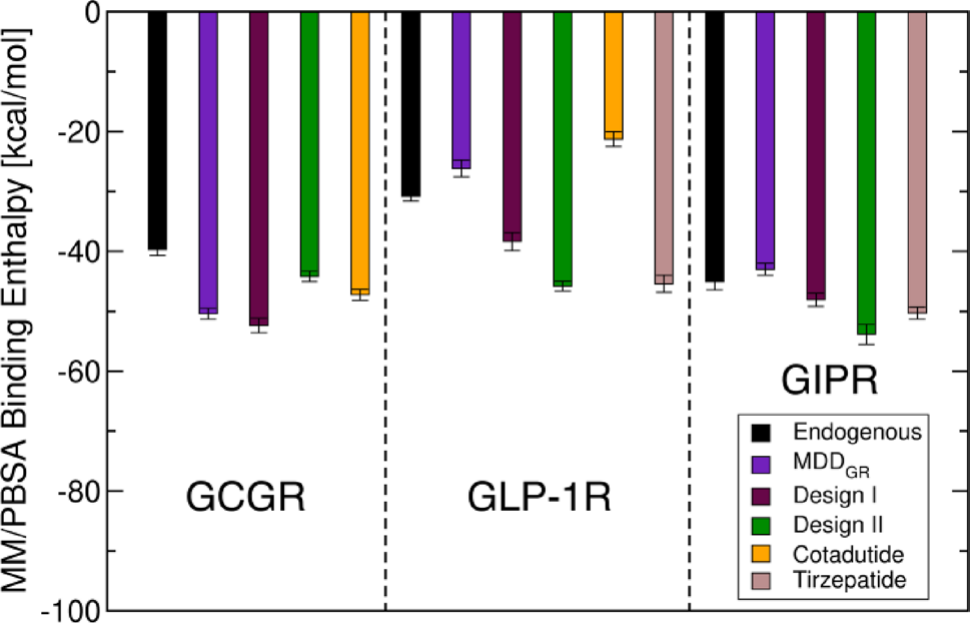
Predicted MM/PBSA effective binding enthalpies (Δ*H*_eff_ in kcal/mol) of agonist peptides in complex with GCG, GLP-1, and GIP receptors, averaged over the final 50 ns of molecular dynamics and across replicate runs. Enthalpy ranking according to the Δ*H*_eff_ values: GCGR—Design I > MDD_GR_ > Design II > Cotadutide > Endogenous ligand (glucagon); GLP-1R—Design II ~ Tirzepatide > Design I > Endogenous ligand (GLP-1) > MDD_GR_ > Cotadutide; GIPR—Design II > Tirzepatide > Design I > Endogenous ligand (GIP) > MDD_GR_

**Fig. 6:**
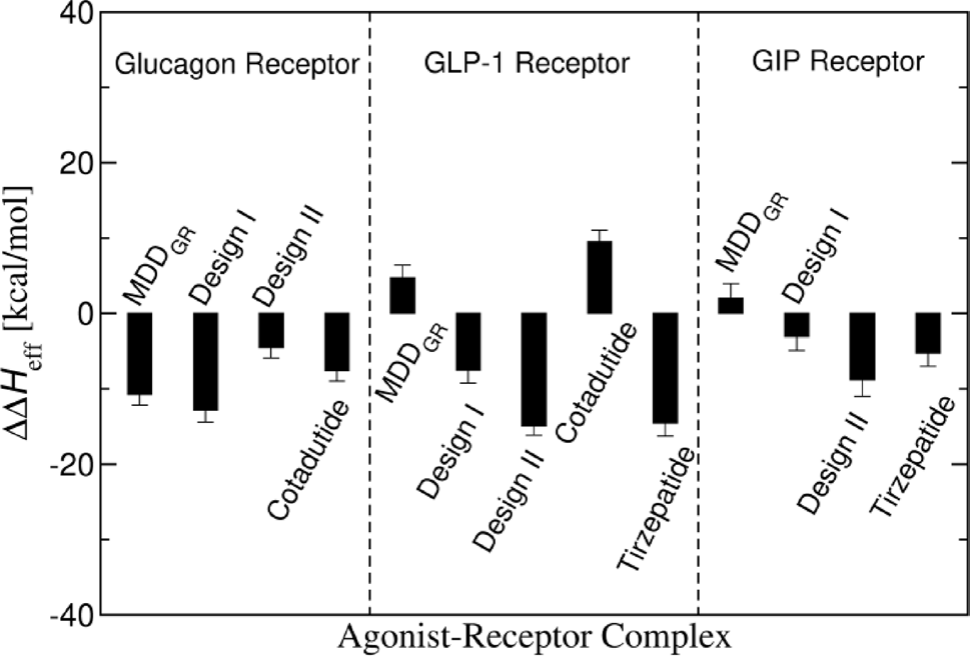
Relative effective binding enthalpies of designed peptide agonists. Effective binding enthalpy difference (ΔΔH_eff_ in kcal/mol) of the computationally designed triple agonist peptides calculated relative to the endogenous ligands (represented by the baseline at zero). Error bars represent the standard error (± SE) from n = 3 independent local sampling replicates

Table 1 lists the eight ligands that were simulated in complex with the three GPCRs: GCGR, GLP-1R and GIPR across 16 models (Fig. 2). Figures 3 and 4 illustrate the binding of the designed triple agonist peptide (Design II) to GCGR, GLP-1R, and GIPR, showing its strong interaction with each receptor compared to their native ligands. The figure highlights key extracellular binding interactions in targeted GPCRs that may contribute to receptor activation, demonstrating how the designed agonist effectively engages with all three targets. Hydrogen bonds formed by the designed triple agonist (Design II) are speculated to stabilise receptor conformations that could facilitate the outward movement of TM6 (Table S2). These binding dynamics predict how the triple agonist may prime each receptor for activation, offering insights into the design's effectiveness in engaging multiple GPCR targets through strategic extracellular interactions. The binding strengths of our designed peptides (Design I and Design II) to the receptors were benchmarked against those of the endogenous and reference peptides (Fig. 5 and Supplementary Note S1). The endogenous peptide ligands sample effective binding enthalpies (Δ*H*_eff_) values (Fig. 5) of − 39 ± 1 kcal/mol, − 30 ± 1 kcal/mol, and − 45 ± 4 kcal/mol with GCGR, GLP-1R, and GIPR, respectively. Figure 6 below shows the average relative effective binding enthalpy (ΔΔ*H*_eff_) profiles (from the individual timelines of Δ*H*_eff_ in Figs. S1, S2A, B), which indicate the potential binding specificities of the designed peptides to GCGR, GLP-1R and GIPR. The energy metrics reported herein represent effective binding enthalpies (${\Delta H}_{eff}$ = ${\Delta E}_{MM}$ + ${\Delta G}_{solvation}$) rather than true Gibbs binding free energies, as the conformational entropy term ($-\mathrm{T}\Delta S$) was not explicitly included. The omission of the entropy term is a recognised limitation of endpoint MM/PBSA methods and is frequently adopted in comparative binding studies owing to the high computational cost and convergence challenges associated with estimating entropy [25, 26]. Consequently, the calculated values should be interpreted as relative thermodynamic descriptors rather than absolute binding free energies.

**Table 1:** Targeted class B1 GPCRs along with their modelled endogenous ligands, designed peptide molecules as triple agonists and reference dual agonists (MDD_GR_^a^ and peptide-based drugs^b^)

Target	Peptide ligands				
GCGR	Glucagon	Design I	Design II	MDD_GR_^a^	Cotadutide^b^
GLP-1R	GLP-1	Design I	Design II	MDD_GR_^a^	Cotadutide^b^ Tirzepatide^b^
GIPR	GIP(1–42)^c^	Design I	Design II	MDD_GR_^a^	Tirzepatide^b^

^a^MDD_GR_ is the dual agonist designed in previous work [17]. ^b^For comparison, we have simulated the reference drug peptides (cotadutide and tirzepatide) without their respective fatty acid modification (which improves albumin binding in vivo but is not relevant for the current binding study—see details under Supplementary Note S1). ^c^GIP is specified as GIP(1–42) here to denote the full-length peptide sequence, distinguishing it from shorter variants

Although the peptides differ in length (30–42 residues), structural analysis indicates that the additional residues in the engineered designs remain largely solvent-exposed and conformationally flexible rather than becoming buried within the receptor interface. Consequently, their effective loss of conformational entropy upon binding is expected to be substantially smaller than theoretical upper-bound estimates based solely on peptide length. Furthermore, incorporation of conformationally constrained Aib residues is expected to reduce the entropic cost of binding. While explicit interaction entropy calculations were not performed, these considerations suggest that the principal affinity rankings reported here are unlikely to be qualitatively altered by entropic effects. Accordingly, all comparisons are discussed in terms of predicted effective binding enthalpies rather than absolute binding free energies (For details, see Supplementary Note S2).

Across the three receptors, Design II maintains more favourable predicted effective binding enthalpies than the corresponding endogenous ligands and benchmark peptides. For GLP-1R and GIPR, these differences substantially exceed the expected magnitude of length-dependent entropic contributions. At GCGR, where the computed enthalpy difference is smaller, the results should be interpreted more cautiously, as they support a qualitative ranking rather than a definitive quantitative distinction.

The computed raw effective binding enthalpy differentials of our designed peptides, extracted from the data in Fig. 5, clearly show their potential binding ability, with the endogenous ligands used as a reference (Table S3). Design I exhibits a more favourable binding enthalpy for GCGR than the native ligands, with an overall Δ*H*_eff_ of − 52.4 ± 1.2 kcal/mol. In addition, Design I shows an improvement in binding enthalpy compared to the native endogenous ligand at GLP-1R and GIPR, with corresponding Δ*H*_eff_ of − 38.4 ± 1.5 kcal/mol and − 48.1 ± 1.1 kcal/mol, respectively. Design II, on the other hand, exhibits a more favourable enthalpy than the native ligands binding to GCGR and GLP-1R, with Δ*H*_eff_ of − 44.2 ± 0.9 kcal/mol and − 45.8 ± 0.8 kcal/mol, respectively. It also has a modestly higher enthalpy for GIPR than for GIP, with a Δ*H*_eff_ of − 53.8 ± 1.7 kcal/mol, resulting in a raw improvement in binding enthalpy over the native hormone ligand. The elevated predicted binding enthalpy of Design II is consistent with stable occupation of the orthosteric binding pocket, providing a structural basis for receptor activation and downstream Gs-mediated intracellular signalling. Design II demonstrates a markedly stronger binding enthalpy for GLP-1R than cotadutide, but it exhibits a weaker calculated value than Design I and cotadutide at GCGR. At both GLP-1R and GIPR, Design II achieves the most favourable binding among all ligands tested, including tirzepatide. Nonetheless, the unmodified peptide helical backbone of tirzepatide exhibits consistently high enthalpy at these receptors, yielding deeper values than the endogenous peptides at GLP-1R and GIPR, and approaching the effective binding enthalpy of Design II at GIPR; however, the ΔΔ*H*_eff_ changes for Design I and II at GIPR, as well as MDD_GR_ at GCGR, are not statistically significant as their error bars overlap, representing neutral variations (Fig. 6).

As an additional predictor of binding potential, we ranked the mean interaction energies of the peptide ligands with the three receptors, GCGR, GLP-1R, and GIPR (Fig. S3; see also Table S4). The interaction energy timelines are plotted in Fig. S4. More favourable interaction energies (*i.e*., those with higher magnitude negative values) reflect the stronger binding of the ligand to the receptor in terms of their electrostatic and van der Waals contacts. The interaction energies represent averages across three replicates, each of which sampled the final 50 ns of dynamics. Our designed peptide ligand, Design II, exhibits the most favourable interaction energies for GCGR and GLP-1R and ranks highly for GIPR, consistent with effective binding enthalpy rankings and supporting its strong overall binding profile. We calculated the standard error in replicate simulations to evaluate the reproducibility of our data. Fig. S5 shows the interaction energies of all sixteen receptor-agonist complexes. The results demonstrate consistency among replicate runs for each system. The triplicate simulation timelines underscore the robustness and reproducibility of our findings, ensuring that all conclusions are based on well-sampled and reliable data. Additional details on the replicate simulations and error analysis are provided in Supplementary Note S3, and information on the secondary structure analyses is provided in Supplementary Note S4.

Design II peptide and tirzepatide show comparably strong interactions with GLP-1R and GIPR. The ranking of interaction energies suggests that our potential tri-agonist-designed peptides (Design I/II) bind most strongly to GCGR and GLP-1R compared to other peptide ligands in this study. That is, Design II shows notable improvement in binding strengths over the endogenous ligands (GCG and GLP-1), the designed dual agonist in our previous work, MDD_GR_, and the reference peptide cotadutide, offering a promising structural candidate for future pharmacological evaluation. GIP, a 42-residue endogenous peptide, shows the most favourable interaction energy with GIPR, followed by Design II, tirzepatide and Design I peptides. GIP also has the strongest binding enthalpy for GIPR compared to both designed and reference peptides (tirzepatide). Overall, our data suggests that the newly designed tri-agonist peptide Design II shows improved computed binding enthalpy relative to the endogenous ligands, indicating balanced triple agonist binding profiles co-targeting the three GCG, GLP-1, and GIP receptors, and affinities comparable to those of reference approved peptide drugs.

### Deciphering the triple-hormone-receptor agonist binding mechanism to GCGR, GLP-1R and GIPR

We note that the effective binding enthalpies (Δ*H*_eff_) of our designed peptide agonists (bearing the modelled residue mutations/substitutions) towards the three studied receptors can differ significantly relative to those of the native glucagon, GLP-1 and GIP hormones. Of particular interest are the residues in peptide ligands that are proximal to receptor transmembrane helices and may mediate ligand binding. Stabilising contacts are evident in the interaction maps shown in Fig. S6. These intermolecular interactions stabilise agonist binding to the extracellular domain (ECD), extracellular loops (ECL1, ECL2, and ECL3), and the transmembrane 7 (TM7) helices of the GPCR during dynamics. The computed agonist binding enthalpies reflect the stability of the receptor-ligand complexes and the contacts formed during the simulation (hydrogen bonds, salt bridges, and hydrophobic interactions), steering the triple-hormone-receptor agonist binding pattern toward class B1 GPCRs (Fig. S6). Figure S7 reports the average number of intermolecular peptide-receptor hydrogen bonds (H-bonds) formed during the MD trajectories. The H-bond timelines (Fig. S8) indicate stable H-bond contact patterns, with average numbers of 12 ± 1, 8 ± 1, and 17 ± 0 H-bonds for the endogenous ligand complexes with GCGR, GLP-1R, and GIPR, respectively. Similarly, stable populations of intermolecular H-bonds form during the Design II MD simulation of 13 ± 1, 17 ± 0, and 9 ± 1 H-bonds to GCGR, GLP-1R, and GIPR, respectively. We also note that our designed peptide (Design II) forms better H-bond contacts with the GCG and GLP-1 receptors, but fewer H-bonds with GIPR than the endogenous peptide ligands, consistent with the computed enthalpic and interaction strengths.

The simulations reveal that critical hydrogen bond interactions between the peptide ligands and receptor residues, particularly at positions 1 and 2 of the peptide N-terminus, support structural stabilisation within the core pocket (Fig. 4). Specifically, hydrogen bonds formed by His1 and Aib2 residues in the Designed Triple Agonist (Design II) contribute to a stable anchoring of the ligand within the binding pocket across the glucagon, GLP-1, and GIP receptors (Table S2). Within these localised production windows, these interactions help maintain the preparatory conformations that are structurally required to eventually facilitate the outward shift of transmembrane helix 6 (TM6), a classic hallmark of long-timescale GPCR activation [17, 27, 28]. This local anchoring represents an essential early state in propagating structural changes toward the intracellular domain (ICD). These newly formed and stabilised hydrogen bonds in the engineered agonist complexes underscore how selective ligand design can fine-tune localised receptor states while mimicking the spatial behaviour of endogenous ligands. Such structural frameworks advance our understanding of receptor dynamics and inform ligand design strategies for optimising GPCR-targeted therapeutics.

### Triple-hormone-receptor agonist-induced GPCR-G_α_ interactions for synergistic activation of GCGR, GLP-1R and GIPR

All three studied receptors (GCGR, GLP-1R and GIPR) in this work are known to activate the alpha subunit of the coupled Gs protein (G_α_) and subsequently trigger the production of cAMP (cyclic adenosine monophosphate), secondary intracellular messengers in GPCR signal transduction [29–31]. Downstream cellular propagation relies on the binding of active receptors with partner heterotrimeric G proteins [32]. Previous studies have reported that incretins and glucagon act by deploying G_α_ for intracellular signalling to stimulate β-cell proliferation and survival [33, 34]. The key role of G_α_ was emphasised in studies showing that mice with β-cell-specific G_α_ deficiency developed diabetes characterised by reduced insulin levels and decreased β-cell proliferation [35]. Hence, to evaluate the structural variations relevant to intracellular downstream signalling pathways, we modelled the dynamics of the heterotrimeric Gs protein in complex with the ligand-bound triple-hormone-receptor agonist. We modelled the interactions between the Gα subunit and receptors in their active states, bound to peptide ligands, to understand how the system's energetics and short-range structural adjustments initialise early-stage assembly. We calculated interaction energy profiles from dynamics of Class B1 GPCRs bound to agonists and the heterotrimeric Gs protein (Gα and Gβγ subunits), measuring the general thermodynamic balance (Fig. S9) between local variations in weakened GPCR-G coupling and improved ligand-GPCR binding. Additionally, we assess the minor spatial variations of Gs protein into its component α and βγ subunits [36, 37] (Fig. S10) by tracking the timelines of distances between the centre of mass (COM) of the subunits (Fig. S11).

The computed molecular dynamics structures show that the Gα subunit exhibits a minor variation relative to the Gβγ subunit by ~ 0.2 nm during the ligand complexation, with the glucagon:GCGR:Gs system showing a variation of 0.3 nm (Fig. S11). These sub-nanometer shifts fall within the range of standard thermal fluctuations and are not interpreted here as full macroscopic dissociation events. The computed interaction energies of GPCR with Gs do not directly correlate with the absolute strength of ligand binding from our models but vary across all simulated agonist:GPCR:Gs systems (Fig. S9). Thus, we propose that different agonists may induce their action by different mechanisms and hence be able to modulate the interactions with bound Gs protein differently, despite binding to the same general receptor binding pocket. We note from previous reports that the Rhodopsin GPCR is activated on the order of milliseconds, and other GPCRs take extended windows to reach their active conformation, fully execute outward TM6 movements, or complete heterotrimeric G-protein separation [38, 39]. Thus, the sub-microsecond sampling times are explicitly characterised to detect only the pre-activation preparatory states and local equilibrium fluctuations, rather than full macroscopic activation pathways or Gs subunit dissociation. With future improvements in HPC, millisecond atomistic MD will become more feasible, and supplementary advanced sampling techniques and mesoscale models, such as coarse-grained [40] modelling of GPCRs, will help further explore the dissociation process of heterotrimeric Gs protein in agonist:GPCR:Gs complexes, building on the present baseline profiles.

The 300 ns production trajectories sample local peptide–receptor interactions, hydrogen-bonding networks and short-range helical fluctuations. Although this timescale is insufficient to characterise large-scale conformational transitions associated with Class B GPCR activation, such as TM6 outward movement or extensive G protein rearrangement, cumulative running-average analysis (Fig. S2B) indicates that the calculated effective binding enthalpies stabilise over the final 50 ns window. The observed ~ 0.2 nm variation in Gα positioning is consistent with thermal fluctuations within an equilibrated complex and is therefore not interpreted here as evidence of receptor activation. Accordingly, the reported ${\Delta H}_{eff}$ values provide a consistent basis for relative comparison of peptide–receptor interactions, while larger-scale conformational transitions remain beyond the scope of the present simulations.

## Discussion

**Fig. 7:**
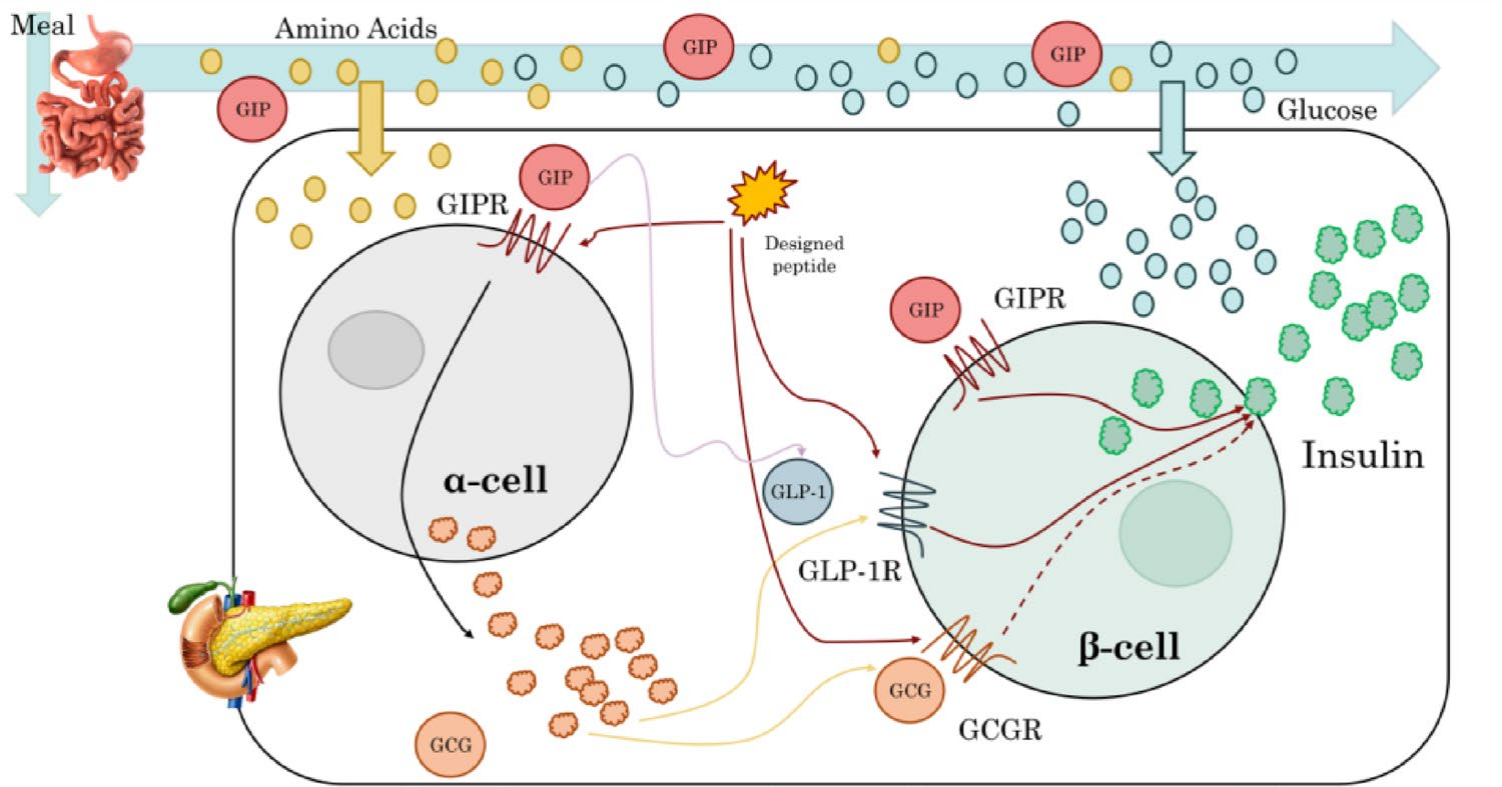
Glucagon/GIP/GLP-1 receptor triple agonism. Interdependence of GCG/GIP/GLP-1 receptor activation and paracrine neighbour-to-neighbour signalling between α and β cells in the pancreatic islets. The metabolic pathway shows that GIP potentiates amino acid-stimulated glucagon secretion and dose-dependently stimulates GLP-1 release. Abbreviations are defined in the text. Figure adapted from El et al*.*[44]. © The Authors, some rights reserved; exclusive licensee AAAS. Distributed under a CC BY-NC 4.0 license http://creativecommons.org/licenses/by-nc/4.0/. Reprinted with permission from AAAS

Previous reports [15, 27, 28, 41] suggest that the development of a synthetic peptide agonist capable of targeting three different hormone receptors that are key metabolic regulators could be a promising molecular approach to target pathways governing glucose homeostasis, adiposity and lipid metabolism (Fig. 7) [18]. With the recent approval of tirzepatide [42, 43] as a dual-hormone-receptor agonist that targets both GLP-1R and GIPR, investigating the potential of simultaneously targeting the GIP receptor with GLP-1 and GCG receptors to develop a triple-hormone-receptor agonist holds immense promise for next-generation peptide-based therapeutics.

The design of the triple agonists in this work was motivated by and extends from our previous work on the design of a 31-residue-long co-agonist peptide [17] targeting both glucagon and GLP-1 receptors simultaneously. Here, our designed unimolecular triple-hormone-receptor agonist is a hybridised peptide, screened and optimised through several iterations of phage-display selection [45] and atomic-level computational modelling (see Methods). The goal was to maintain balanced, highly favourable predicted effective binding enthalpies across all three hormone receptors. Triple-hormone-receptor agonists were further engineered by incorporating three amino acid substitutions/modifications starting from the sequence of our previously designed co-agonist peptide MDD_GR_, with the incorporation of an additional extended C-terminus (Fig. 2A, B).

These engineered modifications were introduced as a rational design strategy intended to reduce susceptibility to DPP-4 and trypsin-mediated degradation. While experimental assay validation is required to demonstrate metabolic stability, we introduced both dextro chiral inversion [46] and nonstandard amino acid substitutions on the second position of the peptide with the intent to protect it from likely DPP-4 cleavage. The extended Exendin-4 C-terminus also provided a secondary beneficial role, in improving binding to these receptors, as evident from the interaction maps (Fig. S6). We note, however, that these modifications were designed strictly as a rational strategy to reduce susceptibility to specific enzymatic cleavage (trypsin and DPP-4), and other proteolytic or chemical degradation pathways were not assessed in this computational study.

Design I and Design II are the two newly designed 39-residue therapeutic peptides in this study (for complete peptide sequence, refer to Fig. 2). Both show comparable or improved computed binding enthalpy relative to the simulated native peptide ligand glucagon and the dual-agonist MDD_GR_ at GCGR (Fig. 6). About GLP-1R, Design II and the unmodified peptide backbone of tirzepatide both show the strongest effective binding enthalpy, followed by Design I and the endogenous ligand GLP-1. Design II enthalpy is also more favourable than that of reference peptides, particularly unmodified peptide backbone of cotadutide, and exhibits an energetic trend comparable to tirzepatide. For GIPR binding, the Design II peptide exhibits the most favourable binding interactions, surpassing both tirzepatide and endogenous peptide GIP in effective binding enthalpy, though tirzepatide shows slightly weaker enthalpic contributions in comparison. The comparative reference peptides used in this study, including tirzepatide, cotadutide and retatrutide, were modelled as unmodified peptide backbones without the clinically incorporated lipid or PEG conjugates. These chemical modifications primarily influence in vivo pharmacokinetic behaviour, serum half-life and systemic exposure rather than intrinsic receptor recognition. Consequently, the calculated ΔHeff values represent intrinsic receptor–peptide interaction enthalpies and should not be interpreted as direct measures of clinical potency.

Overall, Design II demonstrates balanced highly favourable effective binding enthalpy across all three simulated receptors, highlighting its potential as a synergistic tri-agonist. The more favourable, effective binding enthalpies of Design II compared to endogenous peptide hormones indicate that the mutations and nonstandard amino acid modifications in the peptide with an extended C-terminus, enhance receptor engagement. This prediction of enhanced receptor engagement provides a structural model for multi-receptor targeting, which can be evaluated in future experimental studies of downstream metabolic pathways.

To contextualise these designs within the current therapeutic landscape, we also compared our computational models with retatrutide (LY3437943), a clinically advanced triple agonist (Table S5 and Figs. S12, S13). Our thermodynamic analysis shows that retatrutide binds to GIPR (− 40.22 ± 1.24 kcal/mol) and GLP-1R (− 29.56 ± 1.3 kcal/mol) with higher enthalpy than GCGR (− 14.11 ± 1.07 kcal/mol), where it shows a weaker computed binding enthalpy relative to native glucagon (− 39.7 ± 1.1 kcal/mol; Table S6). In comparison, Design II shows improved computed binding enthalpy relative to the native baselines across all three targets (GCGR: − 44.2 ± 0.9, GLP-1R: − 45.8 ± 0.8, GIPR: − 53.9 ± 1.7 kcal/mol; Table S6), demonstrating a well-balanced multi-receptor engagement profile in these simulations. Structural alignments demonstrate that Design II recapitulates the key helical anchoring interactions observed in retatrutide, while introducing additional complementary electrostatic contacts within the receptor binding pocket. The largest difference is observed for GCGR, where Design II exhibits a computed binding enthalpy that is significantly (25.59 kcal/mol) more favourable than the retatrutide control. Similarly, at the GIPR interface, Design II shows improved computed binding enthalpy relative to the unmodified peptide backbones of both native GIP and the retatrutide control (− 53.9 versus − 45.1 and − 40.2 kcal/mol, respectively), supporting balanced tri-receptor engagement within this computational model.

Our computationally designed peptide therapeutics may offer a versatile platform for developing and synthesising triple-acting peptide agonists of class B1 GPCRs and could assist in the future design of bioactive therapeutics with improved pharmacodynamics. While the predicted binding enthalpies of our designed peptides indicate promising trends in receptor targeting, it is important to note and interpret these results in the context of the inherent limitations of MM/PBSA [47]. The method's modular nature and lack of dependence on a training set make it attractive for exploring relative binding trends and guiding computational screening, yet it relies on several approximations, including incomplete treatment of conformational entropy and neglect of contributions from ordered binding-site water molecules [47, 48]. Variants of the method and adjustments to parameters such as residue atomic charges and dielectric constants can substantially affect the computed energies [21, 47]. Accordingly, the calculated Δ*H*_eff_ differences between our designs and reference peptides should be interpreted as qualitative indicators of relative binding behaviour rather than quantitative measures of binding affinity. In particular, ΔΔ*H*_eff_ values for Design I and MDDGR at GIPR and for Design II at GCGR, overlap (Fig. 6), indicating no statistically meaningful difference from the endogenous ligands within the expected systemic uncertainty of MM/PBSA (approximately ± 5–10 kcal/mol) for relative end-state comparisons (see Supplementary Note S2) [48, 49]. These calculations therefore serve primarily to prioritise candidates for future experimental validation. Our modelling data on receptor activation at the sub-microsecond scale predicts that GPCRs can sample many conformations during activation. However, more work in extended/advanced sampling or coarse-grained modelling is needed to better understand the stability of the distinct on/off conformations, whether singular or ensemble, that regulate intracellular signalling networks.

For each complex, three independent molecular dynamics simulations were performed, comprising one original 0.3 µs production run and two repeat simulations of equal duration, resulting in ~ 1 µs of sampling per system. In total, the dataset spans 14.4 µs across all systems. Binding and interaction energies from the repeat simulations were rigorously compared to those from the original trajectories. Replicate simulations were performed for all designed peptides (Design I and II), endogenous ligands, MDD_GR_, and reference peptides, including cotadutide and tirzepatide, across the three receptors. This approach was adopted to validate the consistency of our findings and to ensure robust conclusions. The results presented here thus provide a reliable and comprehensive view of the dynamics and energetics of molecular recognition across the three receptors.

To summarise, our study demonstrates that by selectively and iteratively modifying native hormone sequences through in silico mutagenesis, we rationally designed a new therapeutic peptide—Design II. This peptide incorporates a core design hypothesis intended to mitigate DPP-4 and trypsin enzymatic degradation, while being computationally predicted to exhibit stable effective binding enthalpies across the GCG, GLP-1, and GIP receptors as a potential triple-hormone-receptor agonist. Further, we provide atomic-level insights into the binding mechanisms of the designed peptide with GCGR, GIPR and GLP-1R, which may guide the development of novel therapies for metabolic disorders. While a direct comparison with retatrutide was performed, comparisons with triple-agonist candidates currently in clinical trials [41, 50], were not performed in this study, our computational peptide modelling workflow provides a framework for such analyses (Supplementary Note S5). Future work could utilise this approach to examine atomic-level binding interactions and receptor engagement of existing triple-agonist therapies. Insights gained from these studies may guide rational optimisation of unimolecular designs and help clarify potential advantages in efficacy and receptor selectivity relative to combination therapies.

However, although MD simulations offer reliable physics-based predictions, experimental validation is essential. This includes peptide synthesis, in vitro testing for receptor activation and signalling, and eventual in vivo efficacy and safety studies. We also note that a triple agonist peptide drug, retatrutide [16], is currently in late-phase clinical development for non-alcoholic fatty liver disease. Pending trial results will further elucidate its efficacy, safety profile, and potential side effects.

## Conclusions

Unimolecular multi-receptor affinity peptides for class B1 G protein-coupled receptors (GPCRs) are a class of therapeutics that can simultaneously activate multiple GPCRs, including the GIP, GLP-1 and glucagon receptors and lead to synergistic agonist effects. These receptors play important roles in regulating glucose metabolism, insulin secretion, and appetite, making them attractive targets for the simultaneous treatment of T2DM and obesity. A GIP/GCG/GLP-1 receptor triple agonist is a peptide therapeutic that can activate all three receptors concurrently. This approach has the potential to address multiple metabolic vectors underlying T2DM, obesity, and related comorbidities, such as non-alcoholic fatty liver disease, by targeting multiple pathways involved in glucose metabolism and appetite regulation. The triple-hormone-receptor agonist design also naturally simplifies dosing, patient compliance, and overall therapeutic management. The incorporation of key groups designed to prevent enzymatic proteolysis establishes a computational hypothesis for evaluating how to make the peptide therapeutic more amenable to oral administration at low dosage, with minimal adverse gastrointestinal effects.

By employing a robust computational modelling pipeline, we report here a unimolecular triple-hormone-receptor agonist (or tri-agonist, or triple agonist) peptide analogue that displays predicted, more favourable, and balanced effective binding enthalpies (Δ*H*_eff_) towards three incretin/secretin receptors belonging to the class B1 GPCR family, compared to the endogenous peptide ligands. Our designed 39-residue long peptide, named Design II, targets the key GLP-1, GCG and GIP receptors mediating metabolically active hormonal regulation. The designed triple agonist peptide shows predicted coupled effective binding enthalpy for GCGR, GLP 1R and GIPR that surpasses endogenous ligands and may, in future studies, serve as a lead candidate for in vitro and in vivo validation of metabolic activity. Our models suggest that the reported unimolecular poly-agonist peptide design rules developed in this work have potential applications for further design of potent pharmacologically active peptides in the future to manage metabolic disorders such as diabetes and obesity.

In particular, further development of the proposed unimolecular multi-receptor targeting peptide for GIP/GCG/GLP-1 represents a promising therapeutic avenue for the simultaneous treatment of T2DM, obesity, NAFLD, and NASH. These peptide therapeutics may provide more comprehensive and effective treatments by targeting multiple receptor pathways involved in metabolic disorders. While our atomistic simulations provide molecular-level insight into the behaviour of the engineered triple agonist peptides, we note that future in vitro binding assays and in vivo metabolic studies are essential to test the therapeutic efficacy and pharmacokinetic profiles of these designed peptides. To the best of our knowledge, such intricate and detailed peptide design rules, including the mapping of the simultaneous activation of three Class B1 GPCRs mediated through biological membranes, have not been reported to date, providing new physiologically relevant, experimentally testable hypotheses and exciting opportunities to guide future polypharmacy drug development.

## Methodology

### Sequence-driven rational design of tri-agonist peptides

Here, we set out to rationally design a triple-acting peptide agonist that could potentially serve as an improved therapeutic peptide intervention, benchmarked against the activity of the currently marketed peptide drugs. Starting from the receptor endogenous ligand peptide sequences, we modelled various peptide sequences with triple-hormone-receptor agonist potential. By engineering these lead amino acid sequences through site-specific in silico mutagenesis, we rationally designed peptide molecules (namely Design I and II, see Fig. 2A) with improved affinities towards all three target receptors (Table 1) [51]. Our recently designed GCGR and GLP-1R co-agonist peptide [17] provided us with a starting point to identify several mutation points on the glucagon (GCG) peptide template to facilitate co-targeting to GIPR and to improve overall resistance to proteolysis. These point mutations mediate the residue-wise and the summed contributions of the designed peptides to the receptor binding enthalpies. The net binding enthalpies are predicted from molecular dynamics simulations that capture the complex Coulomb sum of short- and long-range electrostatic interactions in the receptor binding pocket [52, 53].

Peptide engineering at the 30th residue position (Arg30, Fig. 2A) may improve peptide stability against enzymatic proteolysis (see Table S7 for trypsin cleavage site predictions) [17, 54, 55]. The C-terminal sequences of our Design I and Design II peptides (Fig. 2A) are adapted from Exendin-4, a 39-amino acid agonist of GLP-1 receptor (Fig. 2A) approved for the treatment of T2DM. Exendin-4 has been reported to exhibit improved physicochemical and metabolic stability compared to native peptides GLP-1, GCG, and GIP, due to the improved helical stability provided by its C-terminal sequence (residues 31–39), and resistance to enzymatic degradation [54, 56]. Additionally, the peptide design (Fig. 2A, B) includes a nonstandard amino acid residue at position 2 (Aib, α-amino isobutyric acid or dS, dextroserine) to further protect against proteolytic cleavage by dipeptidyl peptidase 4 enzyme (DPP-4) [55, 57]. We emphasise that while these modifications are intended to improve stability against specific enzymatic pathways, they do not comprehensively address all proteolytic or chemical degradation mechanisms. Our designed peptide molecule also incorporates amidation (-CONH_2_) of the C-terminus to enhance the biological activity of the peptide, akin to native endogenous peptides [58].

### Assessing the binding of designed tri-agonist peptides to GIP, GLP-1, and glucagon receptors coupled to their Gs protein

The recently solved high-resolution crystal structures of the three class B1 GPCRs provide important mechanistic insights into the agonist activity. We construct our starting molecular dynamics models based on GIPR, GLP-1R and GCGR coupled to their intracellular heterotrimeric Gs protein and bound to extracellular mono-agonist small molecules and peptide drugs. Table S1 lists the structures deposited in the protein data bank; receptor and peptide sequence-related information is provided in the 'Sequence Information and Analysis' section of the Supporting Information.

To unravel the endogenous ligand-mediated GIP/GLP-1/glucagon receptor activation via G protein coupling, we first modelled GIP, GLP-1 and GCG receptors coupled to the heterotrimeric Gs protein, in complex with their native endogenous peptide ligands. To prepare initial structures of the complexes, we removed nanobody 35 (Nb-35), a G protein mimetic, from crystal structures of agonist–receptor–Gs complexes (PDB code 6X18, GLP-1—GLP-1R—Gs [59]; PDB code 6WPW, GCG—GCGR—Gs [60]; PDB code 7DTY, GIP—GIPR—Gs [61]) as Nb35 locks the GPCRs in specific conformational states [62], masking the natural dynamics (Table 2 and Fig. S14).

**Table 2:** Details of crystal structures used for simulations

Receptor	GCGR (*Homo sapiens*)	GLP-1R (*Homo sapiens*)	GIPR (*Homo sapiens*)
PDB ID	6WPW (Resolution: 3.1Å)	6X18 (Resolution: 2.1Å)	7DTY (Resolution: 3Å)
Endogenous Ligand	Glucagon (29 residues)	GLP-1(7–37) (31 residues), GLP-1(7–36)NH_2_ (30 residues) and Glucagon (29 residues)	GIP(1–42), GIP(1–30)NH_2_ (42, 30 residues respectively)
Crystalised Agonist	Glucagon derivative ZP3780 (synthetic construct, 29 residues)	GLP-1(1–30)-NH_2_ (*Homo sapiens*, 30 residues)	Gastric inhibitory polypeptide (*Homo sapiens*, 42 residues)
Heterotrimeric Subunit (Gα) 393 residues	Guanine nucleotide-binding protein G(s) subunit alpha isoforms short (*Homo sapiens*)	^a^Guanine nucleotide-binding protein G(s) subunit alpha isoforms short (*Homo sapiens*)	Guanine nucleotide-binding protein G(s) subunit alpha isoforms short (*Bos taurus*)
Heterotrimeric Subunit (Gβ) 339 residues	Guanine nucleotide-binding protein G(I)/G(S)/G(T) subunit beta-1 (*Homo sapiens*)	^a^Guanine nucleotide-binding protein G(I)/G(S)/G(T) subunit beta-1 (*Homo sapiens*)	Guanine nucleotide-binding protein G(I)/G(S)/G(T) subunit beta-1 (*Rattus norvegicus*)
Heterotrimeric Subunit (Gγ) 67 residues	Guanine nucleotide-binding protein G(I)/G(S)/G(O) subunit gamma-2 (*Homo sapiens*)	^a^Guanine nucleotide-binding protein G(I)/G(S)/G(O) subunit gamma-2 (*Homo sapiens*)	Guanine nucleotide-binding protein G(I)/G(S)/G(O) subunit gamma-2 (*Bos taurus*)
G-protein mimetic nanobody	Nb35 (Lama glama)	Nb35 (*Lama glama*)	Nb-35 (synthetic construct)

^a^Refer to Table S1 for more details of the subunits

All mutated or missing residues in the extracellular domain (ECD), extracellular loops (ECLs), intracellular loops (ICLs) and termini of receptors, endogenous ligands and heterotrimeric Gs proteins were modelled using the Robetta [63] tool. In addition to the three GPCR receptors in complex with their native ligands (Fig. S15), we modelled 13 different systems to assess the binding enthalpies of the reference and designed drug peptides in complex with GLP-1/GIP/GCG receptor-Gs using CHARMM-GUI PDB Manipulator [64]. Parameters for the nonstandard residues α-amino isobutyric acid (Aib) and dextroserine (dS) were obtained from CHARMM-GUI [64] and the SwissSidechain library [65], respectively, ensuring compatibility with the CHARMM36m force field parameters. Details of all modelled systems are provided in Table S8. The PPM server [66] was utilised to orient the protein complexes to ensure the correct orientation of the receptor transmembrane domain (TMD) inside the fully atomistic POPC (1-palmitoyl–2-oleoyl-sn-glycero-3-phospho­choline) membrane bilayer model, and the agonist–receptor–Gs protein complexes were then embedded in the POPC lipid bilayer.

### Molecular dynamics simulations

All molecular dynamics (MD) simulations were performed using the GROMACS 2018.4 package [67, 68]. This version was selected because it was the well-validated production version available when these simulations were initiated and ensured methodological consistency with our previous dual agonist study [17] and this triple agonist design. CHARMM36m force field parameters [69] were used to represent the agonist–receptor–Gs protein complexes and the POPC phospholipid membrane bilayers. The agonist–receptor–Gs protein–membrane complex was solvated by filling the area above and below the membrane with water molecules represented by the CHARMM-modified TIP3P [69] water model to create a 15 Å thick water layer above the protein and below the membrane to mimic bulk solvation in the z-plane normal to the membrane (Fig. S15). Any excess charges were neutralised by adding the appropriate number of Na^+^ or Cl^−^ counterions [67], and additional NaCl was added to maintain a physiological salt concentration of 0.15 M. Rectangular periodic boundary conditions (PBC) were applied for all systems in all three dimensions, and the Particle Mesh Ewald (PME) method was used to treat all long-range electrostatic interactions. Covalent bonds to hydrogen atoms were constrained using the LINCS algorithm. We first relaxed the systems using 5000 steps of steepest descent energy minimisation, followed by slow heating of the system to 310 K as positional and dihedral restraints were gradually relaxed during 50 ns using a simulation timestep of 1 fs. The restraint-free 0.3 µs production dynamics runs were carried out for each system with a time step of 2 fs in the isothermal–isobaric ensemble (constant pressure–temperature, NPT) at 310 K and 1 bar pressure using the Nosé-Hoover thermostat. Semi-isotropic pressure coupling was applied using the Parrinello Rahman barostat to allow the lipid bilayer to fluctuate in the *xy*-plane independent of the *z*-axis.

All systems exhibited well-equilibrated, ergodic dynamics during the 0.3 µs dynamics as monitored by the temporal evolution of receptor cumulative average secondary structure elements (Fig. S16, with details provided in Supplementary Note S4). We note that the receptor secondary structures are slightly (~ 2%) more stable in helical content when complexed with the new Design II peptide agonist than when complexed with the previous MDD_GR_ agonist [17], exhibiting stabilities more similar to those bound to their endogenous peptide ligands (Table S9). To monitor the structural changes of simulated Gs protein due to the influence of extracellular ligand binding to receptors, we mapped the timelines of secondary structures for heterotrimeric Gs subunits (Figs. S17–S19), and the analysis indicates that Gs protein subunits, when in complex with target receptors, exhibit substantial helical structural content, with GLP-1R—Design II showing the highest percentage of helical content (Table S9). The consistent performance of Design II in stabilising Gs protein secondary structural content across receptor targets suggests a robust strategy for designing peptide ligands that promote the formation of helical structure in Gs proteins. Convergence across three independent replicates was assessed through secondary structure analyses, as shown in Supplementary Figs. S20–S22.

The atomistic molecular dynamics protocol employed in this study follows our previously validated workflow for agonist-bound Class B GPCR systems [17]. To ensure that the present study is fully self-contained, all simulation parameters specific to the current systems are summarised in Table S10, including system composition, equilibration settings, force-field parameters, and MM/PBSA analysis details. Binding enthalpies were calculated using the single-trajectory implementation of gmx_MMPBSA, with frames extracted every 200 ps throughout the 300 ns production trajectories. Final thermodynamic metrics were calculated from the equilibrated 250–300 ns sampling window.

A summary of the modelling datasets is given below (for more details, see Table S8). Dataset I contains six MD simulations of the designed peptide agonists (Design I and Design II) in complex with the GLP-1, GIP and GCG receptors. Dataset II contains six MD simulations of the endogenous agonist and the previously MD-directed designed peptide (MDD_GR_) [17] in complex with the GLP-1, GIP and GCG receptors. Dataset III contains four MD simulations of reference dual agonists (cotadutide and/or tirzepatide) in complex with the GLP-1, GIP and GCG receptors. We investigated tirzepatide as a reference peptide drug to compare the binding enthalpy of the designed triagonist peptide specifically with the GLP-1 and GIP receptors. At the same time, cotadutide was modelled with the GCG and GLP-1 receptors, as both peptides are known to exhibit dual agonistic activity (Supplementary Note S1). All simulations were performed under physiological conditions with the receptor coupled to the Gs protein in a POPC membrane in a fully solvated environment. Each of the 16 complexes was sampled in triplicate across 0.3 µs MD runs, during a total cumulative sampling of 16 × 3 × 0.3 = 14.4 µs of ligand–protein binding dynamics. We conducted replicate simulations for all peptide-receptor complexes, including those with endogenous ligands (glucagon, GLP-1 and GIP), to ensure the reliability and consistency of our findings (see Supplementary Note S3 for details). We calculated ligand–receptor effective binding enthalpies using the Molecular Mechanics Poisson-Boltzmann Surface Area (MM/PBSA) [47, 59, 70] approaches implemented in the gmx_MMPBSA tool [21] (Supplementary Note S2 and Table S6). The internal protein (solute) dielectric constant is set to *ε*_*in*_ = 4, and the external implicit bulk water solvent is set to *ε*_*out*_ = 80. These parameters were applied consistently across all ligand–protein complexes, including designed peptides, the endogenous ligand, and the reference peptide, ensuring that the calculated binding enthalpies are directly comparable and physically meaningful [21, 71] within the implicit solvent framework.

## Supplementary Information

Below is the link to the electronic supplementary material.

Supplementary Material 1

## Data Availability

Data and Software Availability. The focus of our manuscript is on the computational design strategy for therapeutic peptides. The GROMACS 2018.4 software used in this work to produce the molecular dynamics trajectories is free and open-source software. VMD 1.9.3 and the XMGrace programs were used for visualisation and plotting, respectively. All peptide models developed in our computational study were designed with their dynamic self-consistency ascertained from the cross-correlation of predicted properties and benchmarked against the available experimental peptide-based drugs and native hormones. Structural ensembles data that support the findings of this study are openly available for download on Zenodo at 10.5281/zenodo.21384682, reference number [72]. Supporting Information. The Supporting Information (SI) comprises Supplementary Notes S1–S5, Tables S1–S10, Figures S1–S22, and a section on Sequence Information and Analysis.
