# Supplementary material for: Design of a metabolically-stable peptide therapeutic with triple-hormone-receptor agonist activity
Source: J Comput Aided Mol Des. 2026 Jul 30;40(1):191. doi: 10.1007/s10822-026-00903-5 (PMC13424190; doi:10.1007/s10822-026-00903-5)
Supplement: Supplementary file 1 — Supplementary Material 1 [file 10822_2026_903_MOESM1_ESM.pdf]

Supporting Information

Design of a Metabolically-Stable Peptide Therapeutic with Triple-Hormone-Receptor Agonist Activity

Shubham Vishnoi[1, 2], Sarah Hudson[2], Shayon Bhattacharya[3, 4*] and Damien Thompson[1*]

[1]Department of Physics, SSPC the Research Ireland Centre for Pharmaceuticals, Bernal Institute, University of Limerick, V94 T9PX, Ireland.

[2]Department of Chemical Sciences, SSPC the Research Ireland Centre for Pharmaceuticals, Bernal Institute, University of Limerick, V94 T9PX, Ireland.

[3]Department of Biological Sciences, SSPC the Research Ireland Centre for Pharmaceuticals, Bernal Institute, University of Limerick, V94 T9PX, Ireland.

[4]Health Research Institute, University of Limerick, V94 T9PX, Ireland.

Table of Contents

Supplementary Notes

S1. A brief note about reference dual-acting peptide drug molecules S4

S2. Effective binding enthalpy calculations S5

S3. Replicate simulation strategy and comparison S6

S4. Secondary structure analyses S7

S5. Current landscape of triple-agonist candidates in clinical trials and rationale for unimolecular design S8

List of Tables

Table S1. List of structures of Class B1 GPCRs crystalised in active states S10

Table S2. Hydrogen bonding interactions observed in MD-simulated peptide-receptor complexes S12

Table S3. *In silico* receptor binding enthalpies of designed agonist peptides compared to the endogenous ligands S13

Table S4. Interaction energies of the last 50 ns of simulations. S14

Table S5. Effective binding enthalpies for clinical benchmark control, retatrutide (kcal/mol) S14

Table S6. Details of repeat simulations and system parameters S15

Table S7. Trypsin cleavage site predictions on designed GCG/GLP-1 dual agonist (MDD_{GR}) (PeptideCutter) S16

Table S8. List of the simulated systems with the simulations' lengths (in ns) S16

Table S9. Calculated mean helical propensities. S18

Table S10. Simulation parameters for the representative retatrutide:GLP-1R:Gs complex system S19

Sequence Information and Analysis S21

List of Figures

Fig. S1. The timelines of calculated effective binding enthalpies for agonist/receptor complexes S27

Fig. S2. Timelines of effective binding enthalpies binding energies for replicate simulations S28

Fig. S3. Comparison of interaction energy of different simulated agonist peptides in complex with GCG/GLP-1/GIP receptor S29

Fig. S4. The timelines of calculated interaction energies for agonist/receptor complexes S30

Fig. S5. Comparison of interaction energies for replicate simulations S30

Fig. S6. Interaction maps of different agonists and receptor complexes. S31

Fig. S7. Agonist-receptor intermolecular hydrogen bonds S32

Fig. S8. The timelines of the calculated number of H-bonds for agonist/receptor complexes S32

Fig. S9. Calculated interaction energies between Agonist:Receptor, Gα:Receptor and Gβγ:Receptor S33

Fig. S10. Activation of the Gsα subunit of a GPCR. S33

Fig. S11. Calculated COM-COM distances between Gα and Gβγ groups. S34

Fig. S12. Structural comparison and binding pocket alignment of Design II and retatrutide across Class B1 GPCR complexes. S35

Fig. S13. Secondary structure stability and thermodynamic binding enthalpies of retatrutide, in complex with its three targets S36

Fig. S14. Structure of the representative receptor (glucagon receptor) from the Class B1 GPCR family. S37

Fig. S15. Models of endogenous agonist-bound GCG, GIP, and GLP-1 receptors complexed with heterotrimeric Gs protein. S38

Fig. S16. Secondary structural changes in the class B1 GPCRs throughout simulations of peptide agonist:receptor:Gs complex. ... S39

Fig. S17. Structural dynamics of the simulated Gs protein due to the influence of extracellular ligand binding to GCGR. S39

Fig. S18. Structural dynamics of the simulated Gs protein due to the influence of extracellular ligand binding to GLP-1R............. S40

Fig. S19. Structural dynamics of the simulated Gs protein due to the influence of extracellular ligand binding to GIPR. S40

Fig. S20. Secondary structure of GCGR with modelled peptide ligands, three replicates.... .... S41

Fig. S21. Secondary structure of GLP-1R with modelled peptide ligands, three replicates S42

Fig. S22. Secondary structure of GIPR with modelled peptide ligands, three replicates.. S43

Supplementary Note

S1. A brief about reference dual-acting peptide drug molecules

Cotadutide: The experimental drug cotadutide mimics the satiating effect of eating, by modulating the hepatic glycogen and fat content and is currently in phase 2b clinical trials as a dual receptor peptide agonist with balanced GCG/GLP-1 activity[1]. A recent study demonstrates that cotadutide stimulates GCGR to reduce hepatic glycogen and steatosis (fat accumulation in the liver cells), and studies of diet-induced obese mice demonstrate that cotadutide improves insulin sensitivity and restores normal insulin secretion[2]. We used this peptide as a reference to compare the designed triple agonist peptides against the glucagon and GLP-1 receptors. The effective binding enthalpy profiles of cotadutide for GLP-1 and glucagon receptors was evaluated and characterised, followed by a comparison of the enthalpic profiles of the designed triagonist peptides. We also benchmarked the predicted agonistic effect against our previously designed GCG/GLP-1 co-agonist (MDD_{GR})[3] together with the reference endogenous peptide ligands and the cotadutide drug.

Tirzepatide: The recent FDA approval of tirzepatide, a GIP/GLP-1 receptor co-agonist, marks a significant milestone in the development of dual-receptor agonists for the treatment of T2DM, with promising results to date[4,5]. Tirzepatide is a synthetic linear peptide containing 39 amino acids derived from the native GIP sequence. Tirzepatide works by activating both the GIP and GLP-1 receptors, which leads to increased insulin secretion and reduced glucagon secretion, resulting in lower blood glucose levels. It also promotes weight loss and has been shown to have beneficial effects on cardiovascular risk factors, such as blood pressure and lipid levels[5]. In clinical trials, tirzepatide has demonstrated significant improvements in glycaemic control, weight loss, and cardiovascular risk factors compared to other diabetes medications, such as insulin and GLP-1 receptor agonists[6,7]. However, it has also been associated with gastrointestinal side effects, such as nausea, vomiting, and diarrhoea, which may limit its use in some patients[7]. Overall, tirzepatide represents a promising new treatment option for type 2 diabetes and could potentially offer advantages over current therapies in terms of its efficacy and cardiovascular benefits[8]. We investigated tirzepatide as a reference peptide drug to compare the effective binding enthalpy of the designed triagonist peptide for GLP-1 and GIP receptors.

S2. Effective binding enthalpy calculations

Effective Binding enthalpy can be expressed as **Eq. 1**:

$$\Delta H_{\mathrm{eff\,[MM/PBSA]}} = \Delta E_{\mathrm{MM}} + \Delta G_{\mathrm{solvation}} \quad (1)$$

$\Delta H_{\mathrm{eff\,[MM/PBSA]}}$ is the predicted effective binding enthalpy, ΔE_{MM} is the molecular mechanical energy changes in the gas phase, and $\Delta G_{\mathrm{solvation}}$ is the free energy of solvation. This method combines molecular mechanics energies with a Poisson-Boltzmann continuum solvation model and a surface-area term to estimate relative binding strengths. MM/PBSA involves approximations in entropy estimation, implicit treatment of water, and sensitivity to force field parameters, atomic charges and dielectric constants. Computed absolute values, therefore, do not represent true Gibbs free energies (ΔG) because the conformational entropy penalty ($-\mathrm{T}\Delta S$) is omitted. These absolute enthalpy values are approximate and must be interpreted with caution. However, relative trends, that is, calculated binding enthalpy differences comparing one ligand with another within the same computational framework, provide valid and practical guidance for peptide design and prioritisation.

Predicted binding enthalpy profiles and methodological considerations: In interpreting the predicted binding profiles of the designed peptides, it is important to acknowledge the limits of the computational framework. The MM/PBSA approach applied here provides a practical estimate of relative binding strength, but sample size, entropic treatment, and model parameter choices can introduce uncertainty in absolute values. To contextualise the results, simulations of native peptides and reference therapeutic peptides were included as comparative benchmarks, which provide a reference point for assessing the relative effects of the newly designed agonist peptides. The findings reveal consistent trends in receptor engagement; however, they remain computational predictions, and future assay studies will be required to determine whether the proposed interactions and potential metabolic effects are realised experimentally.

The reported metrics represent effective binding enthalpies (ΔH_{eff}). Because the evaluated peptides range in length from 30 to 42 residues, their relative entropic penalties vary with the number of rotatable bonds. While standard isotropic models overestimate the general conformational entropy variance ($-\mathrm{T}\Delta S$) for the 10-residue discrepancy against GCG between 5.0 to 11.0 kcal/mol (or reaching up to ~16.8 kcal/mol when combining global unscaled literature averages of 0.73±0.27 kcal/mol for backbone[9] and 0.95 kcal/mol for side-chains[10]), a residue-specific quantification

restricts this penalty far below the theoretical upper limit. This 10-residue C-terminal extension consists entirely of an Exendin-4-derived tail (GPSSGAPPPS), containing 4 Prolines (0.10 backbone / 0.06 side-chain loss each), 3 Serines (0.79 / 1.11 each), 2 Glycines (0.94 / 0 each), and 1 Alanine (0.65 / 0 each). Residue-specific quantification reduces the maximum theoretical entropic cost to a rigid upper bound of 8.87 kcal/mol (5.3 kcal/mol backbone; 3.57 kcal/mol side chain). Most important, because our structural modelling confirms this tail remains highly flexible and mostly solvent-exposed in the extracellular space rather than burying into the receptor pocket, it could retain the vast majority of its fluid freedom. Scaling this penalty to a realistic 20-30% operational restriction yields a true entropic cost of only ~1.8-2.6 kcal/mol, ensuring that Design II's localised enthalpy margin against native GCG ($\Delta\Delta H_{\mathrm{eff}}$ = -4.5±1.4 kcal/mol) remains stable and insulated from ranking inversion. This structural robustness is further augmented by the inclusion of conformationally constrained Aib modifications within our designs, which minimise the entropic penalty of binding.

On the remaining targets, the calculated enthalpy margins comfortably exceed the entropic threshold. On the GLP-1 Receptor, Design II exhibits a ΔH_{eff} of -45.8±0.8 kcal/mol compared to -30.8±0.8 kcal/mol for native GLP-1 and -21.3±1.2 kcal/mol for cotadutide, establishing favourable enthalpy gaps $\Delta\Delta H_{\mathrm{eff}}$ of around -15.0 kcal/mol and -24.5 kcal/mol, respectively. Against the 42-residue native GIP (ΔH_{eff} = -45±1.4 kcal/mol), our 39-residue Design II (-53.8±1.7 kcal/mol) is shorter by 3 residues. This shorter length yields an inherently lower entropic penalty (~1.5 kcal/mol less penalty), which mathematically widens our reported $\Delta\Delta H_{\mathrm{eff}}$ margin of -8.8±2.2 kcal/mol rather than threatening an inversion. We note that, although the raw interaction energies (ΔE, **Table S4**) for the designs at GIPR are weaker than those of the native counterpart, this is completely offset by a significantly reduced desolvation penalty, yielding a net superior effective binding enthalpy (**Table S3**). Finally, when compared against tirzepatide, which is perfectly length-matched at 39 residues, the length-dependent entropic penalty cancels out entirely. This clean cancellation validates the robust, enhanced affinity trend observed for Design II across both GLP-1R and GIPR (-45.8 vs -45.4 kcal/mol on GLP-1R, and -53.8 vs -50.3 kcal/mol on GIPR).

S3. Replicates of Simulations

To assess the reproducibility and consistency of our molecular dynamics simulations, we performed three independent replicate simulations (triplicates) for each of the sixteen receptor-agonist complexes (**Supplementary Table S6**). These included the endogenous ligands (glucagon, GLP-1 and GIP), the clinical multi-agonists (cotadutide, tirzepatide), a

reported GCG/GLP-1 receptor dual-agonist (MDD_{GR}), and two designed peptides (Design I and Design II), each in complex with their respective receptors (GCGR, GLP-1R and GIPR). Each replicate simulation was run for 0.3 μs, resulting in a cumulative simulation time of 14.4 μs across all systems. For each complex, two independent repeat simulations were performed in addition to the original run, with all simulations initiated from identical starting structures but using distinct random seeds and velocity assignments. These simulations were performed independently to evaluate the reproducibility of binding enthalpies and interaction energies supported by standard error analysis.

Binding interaction energies were calculated using gmx_MMPBSA and analysed over the full 0.3 μs trajectory for each replicate. To evaluate reproducibility, effective binding enthalpy timelines were compared across the three replicates per system, and the average profiles were overlaid (**Fig. S1 and S2**). The similarity of the energy traces and their low variability, supported by standard error estimates, demonstrates the robustness of the simulations and the stability of peptide-receptor interactions over time. We further analysed the timelines of interaction energies for all complexes, including design peptides and the endogenous ligands with their respective receptors. These enthalpic profiles were compared across triplicate simulations to assess consistency, with standard error represented by error bars in the bar chart. In the timeline plot, all three replicates, along with their average profile, are shown for direct comparison (**Fig. S3-S5**). To evaluate reproducibility, we used standard error analysis via block averaging to assess the effective binding enthalpies and interaction energies for each system (**Supplementary Table S6**). For each replicate, average binding enthalpies and interaction energies were calculated over the final 50 ns of the trajectory. Standard errors for these segments were estimated using block averaging in the gmx analyze tool[11], providing a measure of the variability of the energy within each trajectory. For **Table S6**, the final averages reported for each complex were calculated from the three replicate trajectories over the last 50 ns. The associated standard errors were calculated as weighted standard errors based on the estimated errors of the individual replicates.

S4. Secondary structure analyses

The DSSP (Database of Secondary Structure in Proteins) tool by Kabsch and Sander, applied through *GROMACS* tools for secondary structure analyses, was used to analyse the changes in

secondary structural features in the simulated receptors in complex with endogenous and designed peptides[12]. The DSSP tool uses H-bonding patterns and other geometric features to assign secondary structure labels to protein residues. Secondary structure patterns were plotted for GCGR, GLP-1R, and GIPR for all 16 simulated systems to assess convergence during simulations in complex with different endogenous/designed peptides and associated G proteins (**Fig. S13**). We also checked the secondary structure patterns for associated Gs subunits (G_α, G_β and G_γ) (**Fig. S14-S16**).

Design II exhibits the highest mean helical propensity for GCGR (62.40±0.98), indicating a superior structural stability compared to glucagon and MDD_{GR}. In contrast, for GIPR, GIP (58.99±1.2) shows the most favourable propensity, while both Design II (58.23±0.8) and MDD_{GR} (56.95±0.98) demonstrate lower helical stability, suggesting potential differences in receptor activation dynamics among the ligands across different receptors. These findings are further illustrated by the secondary structure analyses presented in the supporting figures (**Fig. S17-19**), which show replicate panels for the GCGR, GLP-1R, and GIPR receptors, respectively, highlighting the structural variability and reproducibility across ligand designs and simulation replicates.

S5. Current landscape of triple-agonist candidates in clinical trials and rationale for unimolecular design

Drug development targeting triple GLP-1/GIP/GCGR agonists represents a promising route for metabolic disorder therapies, building upon the success of dual agonists. Unimolecular peptides simultaneously engaging GLP-1, GIP and glucagon receptors aim to harness synergistic effects on weight reduction, insulin sensitivity and lipid metabolism, potentially offering advantages over mono- and dual-agonist combination therapies[13]. Preclinical studies suggest that triple agonism may provide greater weight loss and broader metabolic benefits compared to dual agonists[14,15]. Key candidates currently under investigation at the time of writing in Nov 2025 include retatrutide (LY3437943)[16], Efocipegtrutide (HM15211)[17] and SAR441255[18]. Retatrutide, a 39-amino acid peptide, demonstrates triple agonist activity with significant preclinical and early clinical efficacy, achieving up to ~24% weight loss at 48 weeks in phase 2 trials, accompanied primarily by mild to moderate gastrointestinal adverse events[15,16]. Efocipegtrutide has shown reductions in body weight and liver fat content in early-

phase studies, while SAR441255 demonstrated significant weight loss in rodent and non-human primate models with favourable tolerability in initial human studies[15,17,18]. These agents provide important benchmarks for understanding the potential of triple agonism in clinical contexts.

Although several dual agonists, including tirzepatide and cotadutide, are already approved or nearing approval with substantial efficacy, unimolecular triple agonists offer conceptual advantages[14]. By integrating multiple receptor activities within a single peptide, unimolecular designs may reduce variability in pharmacokinetics, harmonise receptor engagement and facilitate optimisation of relative agonist potencies, which are challenging to control when combining separate mono- or dual-agonist therapies[15]. Furthermore, the clinical response to receptor agonism may vary across individuals, suggesting that unified unimolecular peptides could support more consistent therapeutic effects.

While direct predictive modelling comparisons with triple-agonist candidates in clinical trials were limited to retatrutide and not extended to other in this study, our computational peptide-modelling workflow provides a platform to evaluate atomic-level binding interactions and receptor engagement. This framework can guide the rational design of novel unimolecular triple agonists, offering a systematic approach to predict and optimise binding selectivity, and hence hypothesise receptor engagement profiles. Future studies could utilise the approach presented in the current work to assess current triple-agonist therapies at atomic resolution, informing the development of new hybrid therapeutic peptides.

In summary, the emerging class of triple GLP-1/GIP/GCGR agonists underscores the potential of unimolecular designs to deliver receptor activation profiles and broader metabolic pathway engagement[14,15]. Computationally guided peptide design, as presented here, could offer a complementary strategy for exploring and streamlining this promising therapeutic space.

Tables

Table S1. List of structures of Class B1 GPCRs (GLP-1R, GCGR and GIPR) crystalised in active state (species: Human, method of crystallisation: cryo-EM; source: GPCRdb, 05/01/2022)**.** The structures used in this study are highlighted in yellow.

RECEPTOR					SIGNAL PROTEIN		STRUCTURE LIGAND		
Receptor	PDB	Resolution	Degree active (%)	% of Seq	Family (Subtype)	% of Seq	Name	Type	Function
GLP-1	7S1M	2.4	100	82	Gs (sα)	59	Exendin-4-D-Ala	peptide	Agonist
GLP-1	7S3I	2.5	100	57	Gs (sα)	59	Exendin-4-D-Ala	peptide	Agonist
GLP-1	7RTB	2.1	100	83	Gs (sα)	60	Peptide-19	peptide	Agonist
GLP-1	7EVM	2.5	100	61	Gs (sα)	60	N-Tert-butyl-6,7-dichloroquinoxalin-2-amine	small molecule	Ago-PAM
GLP-1	7DUR	3.3	100	80	Gs (sα)	60	N-Tert-butyl-6,7-dichloroquinoxalin-2-amine	small molecule	Ago-PAM
GLP-1	7KI1	2.5	100	83	Gs (sα)	89	Taspoglutide	peptide	Agonist
GLP-1	7KI0	2.5	100	83	Gs (sα)	53	Semaglutide	peptide	Agonist
GLP-1	7DUQ	2.5	100	83	Gs (sα)	61	N-Tert-butyl-6,7-dichloroquinoxalin-2-amine	small molecule	Ago-PAM
GLP-1	7E14	2.9	100	81	Gs (sα)	48	N-Tert-butyl-6,7-dichloroquinoxalin-2-amine	small molecule	Ago-PAM
GLP-1	7LCI	2.9	100	85	Gs (sα)	62	PF-06882961	small molecule	Agonist
GLP-1	7LCJ	2.8	97	84			PF-06882961	small molecule	Agonist

GLP-1	7LCK	3.2	97	84			PF-06882961	small molecule	Agonist
GLP-1	6XOX	3.1	100	82	Gs (sα)	56	LY3502970	small molecule	Agonist
GLP-1	6X18	2.1	100	83	Gs (sα)	90	GLP-1	peptide	Agonist
GLP-1	6X19	2.1	100	84	Gs (sα)	89	CHU-128	small molecule	Agonist
GLP-1	6X1A	2.5	100	84	Gs (sα)	89	PF-06882961	small molecule	Agonist
GLP-1	7C2E	4.2	100	82	Gs (sα)	48	RGT1383	small molecule	Agonist
GLP-1	6VCB	3.3	100	82	Gs (sα)	57	LSN3160440	small molecule	PAM
GLP-1	6ORV	3	100	61	Gs (sα)	48	TT-OAD2	small molecule	Agonist
GLP-1	6B3J	3.3	100	82	Gs (sα)	49	Exendin-P5	peptide	Agonist
GCGR	6WPW	3.1	100	83	Gs (sα)	56	Glucagon derivative ZP3780	peptide	Agonist
GCGR	6WHC	3.4	100	78	Gs (sα)	56	Dual-agonist peptide P15	peptide	Agonist
GCGR	6LML	3.9	100	83	Gi/o (αi1)	63	glucagon	peptide	Agonist
GCGR	6LMK	3.7	100	83	Gs (sα)	59	glucagon	peptide	Agonist
GIPR	7DTY	3	100	79	Gs (sα)	59	GIP	peptide	Agonist

Table S2. Hydrogen bonding interactions were observed in MD-simulated peptide-receptor complexes at 0.3 µs, with a hydrogen bond cutoff distance of 3.5 Å and angle cutoff of 30°. Focused on key residues in Peptide Ligand positions 1 and 2 across the glucagon, GLP-1, and GIP receptors in complex with their native ligands and the Designed Triple Agonist (Design II).

**Glucagon Receptor in complex with Glucagon**		**Glucagon Receptor in complex with Designed Triple Agonist (Design II)**	
Acceptor Residue (Peptide Ligand)	Donor Residue (Receptor)	Acceptor Residue (Peptide Ligand)	Donor Residue (Receptor)
**SER2-Side**	**ARG353-Side**	GLU3-Side	LYS162-Side
SER8-Side	ASN273-Main	SER8-Side	ASN273-Main
SER11-Side	THR271-Side	ASP9-Side	TYR113-Side
ASP15-Side	MET4-Main	GLU12-Side	ALA1-Main
ASP15-Side	VAL3-Main	GLU15-Side	VAL3-Main
		GLU15-Side	TYR177-Side
		GLU15-Side	MET4-Main
		ASP28-Side	LYS39-Side
Donor Residue (Peptide Ligand)	Acceptor Residue (Receptor)	Donor Residue (Peptide Ligand)	Acceptor Residue (Receptor)
**HSD1-Main**	**ASP360-Side**	**HSD1-Main**	**ASP360-Side**
**SER2-Side**	**ASP360-Side**	**HSD1-Main**	**GLU337-Side**
LYS12-Side	ASP274-Side	**AIB2-Main**	**ASP360-Side**
ARG18-Side	GLN179-Main	TRP16-Side	MET98-Side
ARG18-Side	LYS180-Main	SER39-Side	HSD19-Side
**GLP-1 Receptor in complex with GLP-1**		**GLP-1 Receptor in complex with Designed Triple Agonist (Design II)**	
Acceptor Residue (Peptide Ligand)	Donor Residue (Receptor)	Acceptor Residue (Peptide Ligand)	Donor Residue (Receptor)
ASP9-Side	ARG353-Side	**HSD1-Side**	**LYS360-Side**
GLU15-Side	SER8-Side	**GLU3-Side**	**THR368-Side**
		ASP9-Side	ARG357-Side
		SER11-Side	ARG276-Side
		GLU15-Side	ARG276-Side
		GLU15-Side	LEU9-Main
		ASP21-Side	ALA185-Main
		ASP21-Side	TYR182-Side
		SER39-Side	GLN24-Side
Donor Residue (Peptide Ligand)	Acceptor Residue (Receptor)	Donor Residue (Peptide Ligand)	Acceptor Residue (Receptor)
**HSD1-Main**	**GLU364-Side**	**HSD1-Main**	**GLU341-Side**
**HSD1-Side**	**GLU364-Side**	**HSD1-Main**	**GLU364-Side**
**ALA2-Main**	**GLU364-Side**	**GLU3-Main**	**GLU364-Side**
LYS20-Side	GLU105-Side	SER32-Side	GLU45-Side
TRP25-Side	GLN188-Side	SER32-Main	GLU45-Side
		PRO37-Main	THR28-Side
		SER39-Side	ARG25-Main
**GIP Receptor in complex with GIP**		**GIP Receptor in complex with Designed Triple Agonist (Design II)**	

Acceptor Residue (Peptide Ligand)	Donor Residue (Receptor)	Acceptor Residue (Peptide Ligand)	Donor Residue (Receptor)
**TYR1-Side**	**GLN203-Side**	GLU15-Side	ARG268-Side
GLU3-Side	ARG162-Side	SER32-Main	GLY89-Main
SER8-Side	ASN269-Main	SER33-Main	GLY89-Main
ASP15-Side	ALA11-Main		
ASP15-Side	ARG268-Side		
GLN19-Side	LEU14-Side		
GLN19-Side	GLN9-Side		
GLN20-Side	ASN99-Side		
Donor Residue (Peptide Ligand)	Acceptor Residue (Receptor)	Donor Residue (Peptide Ligand)	Acceptor Residue (Receptor)
**TYR1-Main**	**GLU333-Side**	**HSD1-Main**	**GLU356-Side**
**TYR1-Main**	**GLU356-Side**	**AIB2-Main**	**GLU356-Side**
**ALA2-Side**	**GLU356-Side**		
SER11-Side	GLU267-Side		
LYS16-Side	GLU101-Side		
GLN19-Side	GLN9-Main		
GLN20-Side	ASN103-Side		
LEU26-Side	MET46-Main		
LYS30-Side	ASP45-Side		
HSD38-Side	ASP45-Main		

Table S3. *In silico* receptor binding enthalpies of designed multi-agonist peptides compared to the corresponding endogenous ligand. Averages of effective binding enthalpies were estimated from the final 50 ns of three replicate 0.3 μs simulations for each receptor-agonist complex. Values represent the average effective binding enthalpy in kcal/mol, calculated using gmx_MMPBSA. This analysis allows comparison between the designed peptides and their native counterparts in terms of predicted receptor engagement.

**Peptide Name**	**Peptide Sequence**	**GCG Receptor**		**GLP-1 Receptor**		**GIP Receptor**	
		Binding Enthalpy ΔH_{eff} [kcal/mol]	Relative Differential $\Delta\Delta H_{eff}$ [kcal/mol]	Binding Enthalpy ΔH_{eff} [kcal/mol]	Relative Differential $\Delta\Delta H_{eff}$ [kcal/mol]	Binding Enthalpy ΔH_{eff} [kcal/mol]	Relative Differential $\Delta\Delta H_{eff}$ [kcal/mol]
**Endogenous Ligand (Glucagon, GLP-1 and GIP, respectively for GCCR, GLP-1R and GIPR)**	Native Ligand	-39.7±1	0.0	-30.8±0.8	0.0	-45±1.4	0.0
**Design I**	39 residues	-52.4±1.2	-12.7	-38.4±1.5	-7.6	-48.1±1.1	-3.1
**Design II**	39 residues	-44.2±0.9	-4.5	-45.8±0.8	-15	-53.8±1.7	-8.8

**MDD_{GR}**	31 residues	-50.4±0.9	-10.7	-26.1±1.4	+4.7	-43±1.3	+2
**Cotadutide**	30 residues	-47.2±0.9	7.5	-21.3±1.2	+9.5	NA	NA
**Tirzepatide**	39 residues	NA	NA	-45.4±1.4	-14.6	-50.3±1	-5.3

Table S4. Peptide-receptor interaction energies. Interaction energies were averaged over the final 50 ns of 0.3 μs molecular dynamics simulations with three replicates per complex. Values represent the raw interaction energies and the corresponding raw difference relative to the native ligand baselines.

**Peptide Name**	**Peptide Sequence**	**GCG Receptor**		**GLP-1 Receptor**		**GIP Receptor**	
		Interaction Energy ΔE [kcal/mol]	Relative Differential $\Delta\Delta E$ [kcal/mol]	Interaction Energy ΔE [kcal/mol]	Relative Differential $\Delta\Delta E$ [kcal/mol]	Interaction Energy ΔE [kcal/mol]	Relative Differential $\Delta\Delta E$ [kcal/mol]
**Endogenous Ligand**	Glucagon for GCCR, GLP-1 for GLP-1R, GIP for GIPR	-354.3±9.5	0.0	-339±15.6	0.0	-469.2±9	0.0
**Design I**	39 residues	-431.4±9.3	-77.1	-389.1±10.1	-50.1	-392.6±29.2	+76.6
**Design II**	39 residues	-448.2±5.8	-93.9	-439.7±9.2	-100.7	-401.9±21.1	+67.3
**MDD_{GR}**	31 residues	-380±15.3	-25.7	-348.4±14.8	-9.4	-379.7±39.5	+89.5
**Cotadutide**	30 residues	-386.7±10	-32.4	-337.4±20.6	+1.6	NA	NA
**Tirzepatide**	39 residues	NA	NA	-416.8±8.6	-77.8	-393.2±16.9	+76

Table S5. Effective binding enthalpies for clinical benchmark control, retatrutide (kcal/mol).

**Receptor**	**Reference Benchmark**	**Mean Binding Enthalpy (ΔH_{eff} ± SE)***
GCGR	Retatrutide Backbone	-14.11±1.07
GLP-1R	Retatrutide Backbone	-29.56±1.24
GIPR	Retatrutide Backbone	-40.22±1.30

*Values represent the mean and standard errors extracted from the last 50 ns of a 300-ns single production trajectory window to provide a computational benchmark for comparison with the engineered peptide designs. These three independent 300 ns simulations (one per receptor) were performed for retatrutide, contributing an additional 0.9 μs of production dynamics. Together with the 16 primary systems simulated in triplicate, this yields a cumulative production simulation time of 14.4 μs (**Table S8**).

Table S6. Details of repeat simulations. This table provides a comprehensive summary of the 16 receptor:ligand:Gs complexes investigated, with a particular focus on the ligand:receptor effective binding enthalpies and interaction energies calculated for each system. For each complex, three independent molecular dynamics simulations were performed, comprising one original 0.3 µs production run and two repeat simulations of equal duration, resulting in 0.9 µs of sampling per system. In total, the dataset spans 14.4 µs across all sixteen systems. Effective binding enthalpies and interaction energies from the repeat simulations are rigorously compared to those obtained in the original trajectories. Averages were calculated over the final 50 ns of each trajectory. Final averages from the three replica simulations are reported, together with the weighted standard error (SEw) calculated from the estimated errors of these trajectories (**Supplementary Notes 2 and 4**). The inclusion of standard error estimates enables quantitative assessment of the consistency, reliability and reproducibility of these energy values across independent replicates.

A. Effective Binding Enthalpy (kcal/mol):

Serial Number	Receptor	Ligand	Dataset 1 (ΔH_{eff} ± Error)	Dataset 2 (ΔH_{eff} ± Error)	Dataset 3 (ΔH_{eff} ± Error)	Average ($\Delta H_{eff} \pm SE_w$)	$\Delta\Delta H_{eff}$ ± Error
1	GCGR	GCG	-40.6 ± 2.2	-40.9 ± 1.6	-37.5 ± 1.8	-39.7 ± 1.1	
**2**	**GCGR**	**Design I**	**-66.0 ± 4.7**	**-51.1 ± 1.6**	**-52.2 ± 2.0**	**-52.5 ± 1.2**	**-12.8 ± 1.6**
3	GCGR	Design II	-50.6 ± 2.2	-42.4 ± 1.0	-51.0 ± 4.2	-44.2 ± 0.9	-4.5 ± 1.4
4	GCGR	MDD_{GR}	-52.2 ± 2.4	-51.2 ± 1.0	-36.7 ± 3.7	-50.4 ± 0.9	-10.8 ± 1.4
5	GCGR	Cotadutide	-48.1 ± 3.1	-48.7 ± 1.0	-31.7 ± 3.4	-47.3 ± 0.9	-7.6 ± 1.4
6	GLP-1R	GLP-1	-30.9 ± 1.4	-18.9 ± 1.8	-38.8 ± 1.4	-30.9 ± 0.9	
7	GLP-1R	Design I	-41.3 ± 1.8	-36.2 ± 3.7	-25.0 ± 4.3	-38.4 ± 1.5	-7.5 ± 1.7
**8**	**GLP-1R**	**Design II**	**-46.6 ± 2.2**	**-44.6 ± 1.0**	**-49.0 ± 1.8**	**-45.8 ± 0.8**	**-14.9 ± 1.2**
9	GLP-1R	MDD_{GR}	-35.9 ± 5.5	-25.5 ± 1.7	-25.4 ± 4.0	-26.2 ± 1.5	4.7 ± 1.7
10	GLP-1R	Cotadutide	-16.0 ± 3.8	-21.8 ± 3.4	-22.0 ± 1.4	-21.3 ± 1.2	9.6 ± 1.5
11	GLP-1R	Tirzepatide	-21.6 ± 3.9	-23.0 ± 2.4	-71.8 ± 2.2	-45.4 ± 1.5	-14.5 ± 1.7
12	GIPR	GIP	-38.5 ± 2.4	-44.5 ± 3.8	-49.6 ± 2.0	-45.1 ± 1.4	
13	GIPR	Design I	-51.1 ± 5.8	-48.4 ± 1.8	-47.8 ± 1.5	-48.2 ± 1.2	-3.1 ± 1.8
**14**	**GIPR**	**Design II**	**-57.6 ± 3.4**	**-60.9 ± 7.7**	**-52.1 ± 2.0**	**-53.9 ± 1.7**	**-8.8 ± 2.2**
15	GIPR	MDD_{GR}	-43.8 ± 1.5	-29.9 ± 5.6	-44.5 ± 3.9	-43.1 ± 1.4	2.0 ± 2.0
16	GIPR	Tirzepatide	-42.2 ± 1.6	-54.5 ± 2.0	-56.8 ± 1.7	-50.3 ± 1.0	-5.3 ± 1.7

B. Interaction Energy (kcal/mol):

Serial Number	Receptor	Ligand	Dataset 1 (ΔE ± Error)	Dataset 2 (ΔE ± Error)	Dataset 3 (ΔE ± Error)	Average ($\Delta E \pm SE_w$)	ΔΔE ± Error
1	GCGR	GCG	-290.3 ± 17.9	-410.8 ± 4.2	-361.8 ± 6.5	-354.3 ± 9.6	
2	GCGR	Design I	-464.6 ± 18.7	-410.0 ± 5.7	-419.8 ± 3.6	-431.5 ± 9.4	-77.2 ± 0.2
**3**	**GCGR**	**Design II**	**-425.9 ± 8.2**	**-452.0 ± 4.4**	**-466.8 ± 5.0**	**-448.2 ± 5.9**	**-93.9 ± 3.7**
4	GCGR	MDD_{GR}	-386.0 ± 5.5	-434.9 ± 19.5	-319.4 ± 21.1	-380.1 ± 15.4	-25.8 ± 5.8
5	GCGR	Cotadutide	-419.1 ± 21.5	-393.5 ± 5.8	-347.5 ± 5.1	-386.7 ± 10.8	-32.4 ± 1.3
6	GLP-1R	GLP-1	-354.2 ± 12.7	-367.6 ± 5.2	-295.4 ± 29.0	-339.0 ± 15.7	
7	GLP-1R	Design I	-455.0 ± 9.8	-360.9 ± 7.4	-351.4 ± 13.3	-389.1 ± 10.1	-50.1 ± 5.5

**8**	**GLP-1R**	**Design II**	**-372.6 ± 13.0**	**-449.6 ± 9.3**	**-497.0 ± 5.5**	**-439.7 ± 9.3**	**-100.7 ± 6.4**
9	GLP-1R	MDD$_{GR}$	-335.2 ± 16.3	-361.4 ± 2.5	-348.8 ± 25.8	-348.5 ± 14.9	-9.4 ± 0.8
10	GLP-1R	Cotadutide	-347.9 ± 11.1	-332.4 ± 23.0	-332.2 ± 28.0	-337.5 ± 20.7	1.6 ± 5.0
11	GLP-1R	Tirzepatide	-340.8 ± 10.9	-411.4 ± 6.1	-498.2 ± 9.1	-416.8 ± 8.7	-77.8 ± 7.0
12	GIPR	GIP	-415.0 ± 13.2	-530.8 ± 6.1	-461.9 ± 8.0	-469.2 ± 9.1	
13	GIPR	Design I	-407.3 ± 13.2	-372.2 ± 12.7	-398.5 ± 61.8	-392.7 ± 29.2	76.5 ± 20.1
**14**	**GIPR**	**Design II**	**-430.3 ± 28.7**	**-418.7 ± 15.0**	**-356.9 ± 19.7**	**-402.0 ± 21.1**	**67.3 ± 12.0**
15	GIPR	MDD$_{GR}$	-390.7 ± 31.1	-325.1 ± 58.8	-423.5 ± 28.7	-379.8 ± 39.5	89.5 ± 30.4
16	GIPR	Tirzepatide	-365.4 ± 15.8	-424.3 ± 10.6	-389.9 ± 24.4	-393.2 ± 16.9	76.0 ± 7.8

Table S7. Trypsin cleavage site predictions on designed GCG/GLP-1 dual agonist (MDD$_{GR}$) (PeptideCutter)[19].

Position of Cleavage Sites	Name of Cleaving Enzyme(s)	Resulting Peptide Sequence	Peptide Length [AA]	Cleavage Probability [%]
12	Trypsin	HSEGTFTSDYSK	12	93.8
18		YLEWQR	6	100
30		AQDFVDWLMDTR	12	100
31		G	1	-

Table S8. The 16 simulated systems, run in triplicate for 0.3 μs each, totalling 14.4 μs of dynamics.

**Serial Number**	**System Name**	**Description**
Modelling of agonist-receptor-Gs protein systems		
1	GCGR:GCG:Gs	Glucagon agonist-bound GCGR in complex with heterotrimeric Gs proteins, composed of Gα, Gβ, and Gγ subunits (active), PDB ID: 6WPW
2	GLP-1R:GLP-1:Gs	Glucagon-like Peptide-1 agonist-bound GLP-1R in complex with heterotrimeric Gs proteins, composed of Gα, Gβ, and Gγ subunits (active), PDB ID: 6X18
3	GIP:GIPR:Gs	Glucose-dependent insulinotropic polypeptide agonist-bound GIPR in complex with heterotrimeric Gs proteins, composed of Gα, Gβ, and Gγ subunits (active), PDB ID: 7DTY
Molecular Dynamics-directed Design (MDD) of Co-agonist Peptides		
4	GCGR:MDD$_{GR}$:Gs	Designed dual agonist-bound GCGR in complex with heterotrimeric Gs proteins, composed of Gα, Gβ, and Gγ subunits

5	GLP-1R:MDD$_{GR}$:Gs	Designed dual agonist-bound GLP-1R in complex with heterotrimeric Gs proteins, composed of Gα, Gβ, and Gγ subunits
6	GIPR:MDD$_{GR}$:Gs	Designed dual agonist-bound GIPR in complex with heterotrimeric Gs proteins, composed of Gα, Gβ, and Gγ subunits<br>[MD-directed design peptide, MDD$_{GR}$ originally constructed with the aim of activity towards both receptors, glucagon receptor and GLP-1 receptor, with residue-wise decomposition energy data obtained from PDL-peptides/endogenous ligands and GCG & GLP-1 receptor simulation, and here we have simulated with all three receptors (GCGR, GLP-1R and GIPR) to test the triple agonist activity.]
Designed Tri-agonist Peptides (Design I and Design II; 39 Residues)		
7	GCGR:Design I:Gs	Designed tri-agonist-bound GCGR in complex with heterotrimeric Gs proteins, composed of Gα, Gβ, and Gγ subunits
8	GLP-1R:Design I:Gs	Designed tri-agonist-bound GLP-1R in complex with heterotrimeric Gs proteins, composed of Gα, Gβ, and Gγ subunits
9	GIPR:Design I:Gs	Designed tri-agonist-bound GIPR in complex with heterotrimeric Gs proteins, composed of Gα, Gβ, and Gγ subunits
10	GCGR:Design II:Gs	Designed tri-agonist-bound GCGR in complex with heterotrimeric Gs proteins, composed of Gα, Gβ, and Gγ subunits
11	GLP-1R:Design II:Gs	Designed tri-agonist-bound GLP-1R in complex with heterotrimeric Gs proteins, composed of Gα, Gβ, and Gγ subunits
12	GIPR:Design II:Gs	Designed tri-agonist-bound GIPR in complex with heterotrimeric Gs proteins, composed of Gα, Gβ, and Gγ subunits
Reference Dual-agonist Ligand System		
13	GCGR:Cotadutide	Reference dual agonist-bound GCGR in complex with heterotrimeric Gs proteins, composed of Gα, Gβ, and Gγ subunits (active)
14	GLP-1R:Cotadutide	Reference dual agonist-bound GLP-1R in complex with heterotrimeric Gs proteins, composed of Gα, Gβ, and Gγ subunits (active)
15	GIPR:Tirzepatide	Reference dual agonist-bound GIPR in complex with heterotrimeric Gs proteins, composed of Gα, Gβ, and Gγ subunits (active)
16	GLP-1R:Tirzepatide	Reference dual agonist-bound GLP-1R in complex with heterotrimeric Gs proteins, composed of Gα, Gβ, and Gγ subunits (active)

Table S9. Calculated mean helical propensities (for the last 50 ns of simulations) along with standard deviation across three different receptor targets: GCGR, GLP-1R, and GIPR. Positive percentages indicate a higher helical propensity for Design II, while negative percentages show a lower helical propensity compared to the other ligands.

Target	Ligand	Receptor Mean Helical Propensity (±SD)	Receptor Helical Propensity Improvement of Design II (%)	Gs Protein Mean Helical Propensity (±SD)		Total Gs Protein Helical Structural Content (%)
**GCGR**	Glucagon	60.47±0.96	3.19	Gα	44.97±1.34	57.08±3.44
				Gβ	8.41±0.54	
				Gγ	3.7±3.12	
	Design II	62.40±0.98	-	Gα	42.25±1.08	54.15±3.07
				Gβ	8.82±0.46	
				Gγ	3.08±2.84	
	MDD_{GR}	60.04±1.18	3.93	Gα	42.85±1.86	55.69±3.67
				Gβ	8.18±0.36	
				Gγ	4.66±3.18	
	Cotadutide	61.08±0.94	2.1			
**GLP-1R**	GLP-1	60.47±0.93	-0.63	Gα	45.64±1.14	57.77±3.67
				Gβ	7.6±0.47	
				Gγ	4.53±3.47	
	Design II	60.09±0.89	-	Gα	44.26±1.22	58.68±4.01
				Gβ	8.26±0.33	
				Gγ	6.16±3.79	
	MDD_{GR}	59.58±0.8	0.85	Gα	44.5±1.03	54.27±2.76
				Gβ	7.74±0.36	
				Gγ	2.03±2.53	
	Cotadutide	60.9±0.95	-1.33			
	Tirzepatide	59.31±1.09	1.31			
**GIPR**	GIP	58.99±1.2	-1.28	Gα	45.84±1.38	57.17±3.47

				Gβ	7.56±0.35	
				Gγ	3.77±3.14	
	Design II	58.23±0.8	-	Gα	47.24±1.04	58.24±2.52
				Gβ	7.85±0.65	
				Gγ	3.15±2.24	
	MDD_{GR}	56.95±0.98	2.24	Gα	44.1±1.97	57.93±4.38
				Gβ	7.82±0.34	
				Gγ	6.01±4.03	
	Tirzepatide	59.37±0.98	1.92			

Table S10. Comprehensive structural, spatial and analytical simulation parameters for the representative Design II:GLP-1R:Gs complex system.

Parameter Category	Specific Computational Attribute	Protocol / Parameter Specification
**System Dimensions**	Representative Box Dimensions	11.32 nm × 11.32 nm × 22.05 nm
	Total Atom Population Range	266,320 atoms per system
	POPC Lipid Bilayer Composition	335 total lipids (~167 per leaflet)
	Bulk Solvent Environment	66,946 TIP3P water molecules (199 Na^+, 182 Cl^- counterions)
**Electrostatics & vdW**	van der Waals Cutoff Distance	1.2 nm (with a Verlet force-switch function starting at 1.0 nm)
	Electrostatic Cutoff Distance	1.2 nm
	PME Grid Spacing/Interpolation	0.12 nm grid / 4th-order cubic splines
**Coupling Mechanics**	Thermostat Type/Coupling constant (τ_t)	Nose-Hoover/1.0 ps (for SOLU, MEMB, and SOLV)

	Temperature Coupling Groups	Group 1: SOLU (Solute) I Group 2: MEMB (Membrane) I Group 3: SOLV (Solvent + Ions)
	Barostat Type/Coupling constant (τ_p)	Parrinello-Rahman/5.0 ps (Semi-isotropic scheme; compressibility: 4.5×10^{-5} bar^{-1})
**Equilibration Strategy**	Restraint Force Constants	Max restraints: Backbone: 4000, Sidechain: 2000, Lipids: 1000, Dihedrals: 1000 ($kJ/mol*nm^2$). Min restraints: Backbone: 50, Sidechain: 0, Lipids: 0, Dihedrals: 0 ($kJ/mol*nm^2$)
	Step-down Scheduling	6-step progressive relaxation equilibration phase (step6.1 with dt = 1 fs to step6.6 with dt = 2 fs)
**Replicate Execution**	Statistical Independence	Initialised *via* random Maxwell-Boltzmann velocity distribution at 310 K using unique random integer seeds (gen-seed = -1)
**Residue Specifications**	Non-standard Amino Acids (Aib, D-Ser)	CHARMM36m parameter stream via CHARMM-GUI FF-converter
	Histidine Protonation States	Assigned *via* default CHARMM-GUI structural patch selection at pH 7.4; peptide N-terminal His1 mapped with a protonated main-chain terminal tail (-NH_3^+) and a neutral ($N\delta^-$) side-chain tautomer.
**Enthalpic Analysis**	Core MM/PBSA Configuration	Single-Trajectory protocol executed *via gmx_MMPBSA*
	Trajectory Extraction Interval	Uniformly sampled snapshot extraction at every 200 ps
	Solute Dielectric (ε_{in})<br>Solvent Dielectric (ε_{out})<br>Membrane Dielectric	4.0<br>80.0<br>21.0

Note: Parameters, box dimensions, atom counts and solvent populations reported in this table are derived directly from the representative Design II:GLP-1R:Gs complex configuration. All other simulated Class B1 GPCR setups share the same structural assembly pipeline and force field execution conditions. Details for all 16 simulated systems, each run in triplicate for 0.3 µs, yielding a cumulative 14.4 µs of production dynamics, are provided in **Table S8**.

Sequence Information and Analysis

Note: This section provides all sequence data and relevant details for this study, including information on the endogenous ligand and the full sequences of three receptors: **GCGR** (Glucagon Receptor), **GLP-1R** (Glucagon-Like Peptide-1 Receptor), and **GIPR** (Glucose-Dependent Insulinotropic Polypeptide Receptor). Additionally, the reference drug peptide sequences for **tirzepatide** and **cotadutide** are included. To enhance clarity, the seven transmembrane (TM) helices of each receptor are highlighted in red. This emphasis is intended to aid in identifying these crucial segments within the receptors, which are key to the study.

Receptor: Glucagon receptor

Gene: GCGR, Organism: *Homo sapiens*

```
>sp|P47871|GLR_HUMAN Glucagon receptor OS=Homo sapiens OX=9606 GN=GCGR PE=1 SV=1
MPPCQPQRPLLLLLLLLACQPQVPSAQVMDFLFEKWKLYGDQCHHNLSLLPPPTELVCNR
TFDKYSCWPDTPANTTANISCPWYLPWHHKVQHRFVFKRCGPDGQWVRGPRGQPWRDASQ
CQMDGEEIEVQKEVAKMYSSFQVMYTVGYSLSLGALLLALAILGGLSKLHCTRNAIHANL
FASFVLKASSVLVIDGLLRTRYSQKIGDDLSVSTWLSDGAVAGCRVAAVFMQYGIVANYC
WLLVEGLYLHNLLGLATLPERSFFSLYLGIGWGAPMLFVVPWAVVKCLFENVQCWTSNDN
MGFWWILRFPVFLAILINFFIFVRIVQLLVAKLRARQMHHTDYKFRLAKSTLTLIPLLGV
HEVVFAFVTDEHAQGTLRSAKLFFDLFLSSFQGLLVAVLYCFLNKEVQSELRRRWHRWRL
GKVLWEERNTSNHRASSSPGHGPPSKELQFGRGGGSQDSSAETPLAGGLPRLAESPF
```

Link: https://www.uniprot.org/uniprot/P47871.fasta

Signal peptide (1-25), receptor chain (26-477)

Receptor: Glucagon-like peptide-1 receptor

Gene: GLP-1R, Organism: *Homo sapiens*

```
>sp|P43220|GLP-1R_HUMAN Glucagon-like peptide-1 receptor OS=Homo sapiens OX=9606
GN=GLP-1R PE=1 SV=2
MAGAPGPLRLALLLLGMVGRAGPRPQGATVSLWETVQKWREYRRQCQRSLTEDPPPATDL
FCNRTFDEYACWPDGEPGSFVNVSCPWYLPWASSVPQGHVYRFCTAEGLWLQKDNSSLPW
RDLSECEESKRGERSSPEEQLLFLYIIYTVGYALSFSALVIASAILLGFRHLHCTRNYIH
LNLFASFILRALSVFIKDAALKWMYSTAAQQHQWDGLLSYQDSLSCRLVFLLMQYCVAAN
YYWLLVEGVYLYTLLAFSVLSEQWIFRLYVSIGWGVPLLFVVPWGIVKYLYEDEGCWTRN
SNMNYWLIIRLPILFAIGVNFLIFVRVICIVVSKLKANLMCKTDIKCRLAKSTLTLIPLL
GTHEVIFAFVMDEHARGTLRFIKLFTELSFTSFQGLMVAILYCFVNNEVQLEFRKSWERW
RLEHLHIQRDSSMKPLKCPTSSLSSGATAGSSMYTATCQASCS
```

Link: https://www.uniprot.org/uniprot/P43220.fasta

Signal peptide (1-23), **receptor chain (24-463)**

Receptor: Gastric inhibitory polypeptide receptor

Gene: GIPR, Organism: *Homo sapiens*

>sp|P48546|GIPR_HUMAN Gastric inhibitory polypeptide receptor OS=Homo sapiens OX=9606 GN=GIPR PE=1 SV=1
MTTSPILQLLLRLSLCGLLLQRAETGSKGQTAGELYQRWERYRRECQETLAAAEPPSGLA
CNGSFDMYVCWDYAAPNATARASCPWYLPWHHHVAAGFVLRQCGSDGQWGLWRDHTQCEN
PEKNEAFLDQRLILERLQVMYTVGYSLSLATLLLALLILSLFRRLHCTRNYIHINLFTSF
MLRAAAILSRDRLLPRPGPYLGDQALALWNQALAACRTAQIVTQYCVGANYTWLLVEGVY
LHSLLVLVGGSEEGHFRYYLLLGWGAPALFVIPWVIVRYLYENTQCWERNEVKAIWWIIR
TPILMTILINFLIFIRILGILLSKLRTRQMRCRDYRLRLARSTLTLVPLLGVHEVVFAPV
TEEQARGALRFAKLGFEIFLSSFQGFLVSVLYCFINKEVQSEIRRGWHHCRLRRSLGEEQ
RQLPERAFRALPSGSGPGEVPTSRGLSSGTLPGPGNEASRELESYC

Link: https://www.uniprot.org/uniprot/P48546.fasta

Signal peptide (1-21), **receptor chain (22-466)**

Agonist: Glucagon and GLP-1

Gene: GCG, Organism: *Homo sapiens*

>sp|P01275|GLUC_HUMAN Pro-glucagon OS=Homo sapiens OX=9606 GN=GCG PE=1 SV=3

MKSIYFVAGLFVMLVQGSWQRSLQDTEEKSRSFSASQADPLSDPDQMNEDKRHSQGTFTS

DYSKYLDSRRAQDFVQWLMNTKRNRNNIAKRHDEFERHAEGTFTSDVSSYLEGQAAKEFI

AWLVKGRGRRDFPEEVAIVEELGRRHADGSFSDEMNTILDNLAARDFINWLIQTKITDRK

Hormone Peptide: Glucagon (53-81) and GLP-1 (92-128)

Link: https://www.uniprot.org/uniprot/P01275.fasta

Agonist: Gastric inhibitory polypeptide

Gene: GIP, Organism: *Homo sapiens*

>sp|P09681|GIP_HUMAN Gastric inhibitory polypeptide OS=Homo sapiens OX=9606 GN=GIP PE=1 SV=1
MVATKTFALLLLSLFLAVGLGEKKEGHFSALPSLPVGSHAKVSSPQPRGPRYAEGTFISD
YSIAMDKIHQQDFVNWLLAQKGKKNDWKHNITQREARALELASQANRKEEEAVEPQSSPA
KNPSDEDLLRDLLIQELLACLLDQTNLCRLRSR

Hormone Peptide: Gastric inhibitory polypeptide (52-93)

Link: https://www.uniprot.org/uniprot/P09681.fasta

Guanine nucleotide-binding protein G(s) subunit alpha isoforms short

Crystalised in complex with glucagon agonist-bound GCGR (6WPW) and GLP-1 agonist-bound GLP-1R (6X18)

>sp|P63092|GNAS2_HUMAN Guanine nucleotide-binding protein G(s) subunit alpha isoforms short OS=Homo sapiens OX=9606 GN=GNAS PE=1 SV=1

MGCLGNSKTEDQRNEEKAQREANKKIEKQLQKDKQVYRATHRLLLLGAGESGKSTIVKQM

RILHVNGFNGEGGEEDPQAARSNSDGEKATKVQDIKNNLKEAIETIVAAMSNLVPPVELA

NPENQFRVDYILSVMNVPDFDFPPEFYEHAKALWEDEGVRACYERSNEYQLIDCAQYFLD

KIDVIKQADYVPSDQDLLRCRVLTSGIFETKFQVDKVNFHMFDVGGQRDERRKWIQCFND

VTAIIFVVASSSYNMVIREDNQTNRLQEALNLFKSIWNNRWLRTISVILFLNKQDLLAEK

VLAGKSKIEDYFPEFARYTTPEDATPEPGEDPRVTRAKYFIRDEFLRISTASGDGRHYCY

PHFTCAVDTENIRRVFNDCRDIIQRMHLRQYELL

Link: https://www.uniprot.org/uniprot/P63092.fasta

Crystalised in complex with GIP agonist-bound GIPR (7DTY)

>sp|P04896|GNAS2_BOVIN Guanine nucleotide-binding protein G(s) subunit alpha isoforms short OS=Bos taurus OX=9913 GN=GNAS PE=1 SV=1
MGCLGNSKTEDQRNEEKAQREANKKIEKQLQKDKQVYRATHRLLLLGAGESGKSTIVKQM
RILHVNGFNGEGGEEDPQAARSNSDGEKATKVQDIKNNLKEAIETIVAAMSNLVPPVELA
NPENQFRVDYILSVMNVPDFDFPPEFYEHAKALWEDEGVRACYERSNEYQLIDCAQYFLD
KIDVIKQDDYVPSDQDLLRCRVLTSGIFETKFQVDKVNFHMFDVGGQRDERRKWIQCFND
VTAIIFVVASSSYNMVIREDNQTNRLQEALNLFKSIWNNRWLRTISVILFLNKQDLLAEK
VLAGKSKIEDYFPEFARYTTPEDATPEPGEDPRVTRAKYFIRDEFLRISTASGDGRHYCY
PHFTCAVDTENIRRVFNDCRDIIQRMHLRQYELL

Initiator methionine (1), Guanine nucleotide-binding protein G(s) subunit alpha isoforms short chain (2-394)

Link: https://www.uniprot.org/uniprot/P04896.fasta

```
CLUSTAL O(1.2.4) multiple sequence alignment


sp|P63092|GNAS2_HUMAN      MGCLGNSKTEDQRNEEKAQREANKKIEKQLQKDKQVYRATHRLLLLGAGESGKSTIVKQM 60
sp|P04896|GNAS2_BOVIN      MGCLGNSKTEDQRNEEKAQREANKKIEKQLQKDKQVYRATHRLLLLGAGESGKSTIVKQM 60
                           ************************************************************

sp|P63092|GNAS2_HUMAN      RILHVNGFNGEGGEEDPQAARSNSDGEKATKVQDIKNNLKEAIETIVAAMSNLVPPVELA 120
sp|P04896|GNAS2_BOVIN      RILHVNGFNGEGGEEDPQAARSNSDGEKATKVQDIKNNLKEAIETIVAAMSNLVPPVELA 120
                           ************************************************************

sp|P63092|GNAS2_HUMAN      NPENQFRVDYILSVMNVPDFDFPPEFYEHAKALWEDEGVRACYERSNEYQLIDCAQYFLD 180
sp|P04896|GNAS2_BOVIN      NPENQFRVDYILSVMNVPDFDFPPEFYEHAKALWEDEGVRACYERSNEYQLIDCAQYFLD 180
                           ************************************************************

sp|P63092|GNAS2_HUMAN      KIDVIKQADYVPSDQDLLRCRVLTSGIFETKFQVDKVNFHMFDVGGQRDERRKWIQCFND 240
sp|P04896|GNAS2_BOVIN      KIDVIKQDDYVPSDQDLLRCRVLTSGIFETKFQVDKVNFHMFDVGGQRDERRKWIQCFND 240
                           ******* ****************************************************

sp|P63092|GNAS2_HUMAN      VTAIIFVVASSSYNMVIREDNQTNRLQEALNLFKSIWNNRWLRTISVILFLNKQDLLAEK 300
sp|P04896|GNAS2_BOVIN      VTAIIFVVASSSYNMVIREDNQTNRLQEALNLFKSIWNNRWLRTISVILFLNKQDLLAEK 300
                           ************************************************************

sp|P63092|GNAS2_HUMAN      VLAGKSKIEDYFPEFARYTTPEDATPEPGEDPRVTRAKYFIRDEFLRISTASGDGRHYCY 360
sp|P04896|GNAS2_BOVIN      VLAGKSKIEDYFPEFARYTTPEDATPEPGEDPRVTRAKYFIRDEFLRISTASGDGRHYCY 360
                           ************************************************************

sp|P63092|GNAS2_HUMAN      PHFTCAVDTENIRRVFNDCRDIIQRMHLRQYELL   394
sp|P04896|GNAS2_BOVIN      PHFTCAVDTENIRRVFNDCRDIIQRMHLRQYELL   394
                           **********************************
```

Sequence Alignment Panel: *Sequence alignment between Human and Bovine Guanine nucleotide-binding protein G(s) subunit alpha isoform.*

Guanine nucleotide-binding protein G(I)/G(S)/G(T) subunit beta-1

Crystalised in complex with glucagon agonist-bound GCGR (6WPW) and GLP-1 agonist-bound GLP-1R (6X18)

```
>sp|P62873|GBB1_HUMAN Guanine nucleotide-binding protein G(I)/G(S)/G(T) subunit
beta-1 OS=Homo sapiens OX=9606 GN=GNB1 PE=1 SV=3
MSELDQLRQEAEQLKNQIRDARKACADATLSQITNNIDPVGRIQMRTRRTLRGHLAKIYA
MHWGTDSRLLVSASQDGKLIIWDSYTTNKVHAIPLRSSWVMTCAYAPSGNYVACGGLDNI
CSIYNLKTREGNVRVSRELAGHTGYLSCCRFLDDNQIVTSSGDTTCALWDIETGQQTTTF
TGHTGDVMSLSLAPDTRLFVSGACDASAKLWDVREGMCRQTFTGHESDINAICFFPNGNA
FATGSDDATCRLFDLRADQELMTYSHDNIICGITSVSFSKSGRLLLAGYDDFNCNVWDAL
KADRAGVLAGHDNRVSCLGVTDDGMAVATGSWDSFLKIWN
```

Link: https://www.uniprot.org/uniprot/P62873.fasta

Crystalised in complex with GIP agonist-bound GIPR (7DTY)

```
>sp|P54311|GBB1_RAT Guanine nucleotide-binding protein G(I)/G(S)/G(T) subunit
beta-1 OS=Rattus norvegicus OX=10116 GN=Gnb1 PE=1 SV=4
MSELDQLRQEAEQLKNQIRDARKACADATLSQITNNIDPVGRIQMRTRRTLRGHLAKIYA
MHWGTDSRLLVSASQDGKLIIWDSYTTNKVHAIPLRSSWVMTCAYAPSGNYVACGGLDNI
CSIYNLKTREGNVRVSRELAGHTGYLSCCRFLDDNQIVTSSGDTTCALWDIETGQQTTTF
TGHTGDVMSLSLAPDTRLFVSGACDASAKLWDVREGMCRQTFTGHESDINAICFFPNGNA
FATGSDDATCRLFDLRADQELMTYSHDNIICGITSVSFSKSGRLLLAGYDDFNCNVWDAL
KADRAGVLAGHDNRVSCLGVTDDGMAVATGSWDSFLKIWN
```

Initiator methionine (1), Guanine nucleotide-binding protein G(I)/G(S)/G(T) subunit beta-1 (2-340)

Link: https://www.uniprot.org/uniprot/P54311.fasta

```
CLUSTAL O(1.2.4) multiple sequence alignment

sp|P62873|GBB1_HUMAN      MSELDQLRQEAEQLKNQIRDARKACADATLSQITNNIDPVGRIQMRTRRTLRGHLAKIYA  60
sp|P54311|GBB1_RAT        MSELDQLRQEAEQLKNQIRDARKACADATLSQITNNIDPVGRIQMRTRRTLRGHLAKIYA  60
                          ************************************************************

sp|P62873|GBB1_HUMAN      MHWGTDSRLLVSASQDGKLIIWDSYTTNKVHAIPLRSSWVMTCAYAPSGNYVACGGLDNI  120
sp|P54311|GBB1_RAT        MHWGTDSRLLVSASQDGKLIIWDSYTTNKVHAIPLRSSWVMTCAYAPSGNYVACGGLDNI  120
                          ************************************************************

sp|P62873|GBB1_HUMAN      CSIYNLKTREGNVRVSRELAGHTGYLSCCRFLDDNQIVTSSGDTTCALWDIETGQQTTTF  180
sp|P54311|GBB1_RAT        CSIYNLKTREGNVRVSRELAGHTGYLSCCRFLDDNQIVTSSGDTTCALWDIETGQQTTTF  180
                          ************************************************************

sp|P62873|GBB1_HUMAN      TGHTGDVMSLSLAPDTRLFVSGACDASAKLWDVREGMCRQTFTGHESDINAICFFPNGNA  240
sp|P54311|GBB1_RAT        TGHTGDVMSLSLAPDTRLFVSGACDASAKLWDVREGMCRQTFTGHESDINAICFFPNGNA  240
                          ************************************************************

sp|P62873|GBB1_HUMAN      FATGSDDATCRLFDLRADQELMTYSHDNIICGITSVSFSKSGRLLLAGYDDFNCNVWDAL  300
sp|P54311|GBB1_RAT        FATGSDDATCRLFDLRADQELMTYSHDNIICGITSVSFSKSGRLLLAGYDDFNCNVWDAL  300
                          ************************************************************

sp|P62873|GBB1_HUMAN      KADRAGVLAGHDNRVSCLGVTDDGMAVATGSWDSFLKIWN      340
sp|P54311|GBB1_RAT        KADRAGVLAGHDNRVSCLGVTDDGMAVATGSWDSFLKIWN      340
                          ****************************************
```

Sequence Alignment Panel: *Sequence alignment between Human and Bovine Guanine nucleotide-binding protein G(I)/G(S)/G(T) subunit beta-1.*

Guanine nucleotide-binding protein G(I)/G(S)/G(O) subunit gamma-2

Crystalised in complex with glucagon agonist-bound GCGR (6WPW) and GLP-1 agonist-bound GLP-1R (6X18)

```
>sp|P59768|GBG2_HUMAN Guanine nucleotide-binding protein G(I)/G(S)/G(O) subunit
gamma-2 OS=Homo sapiens OX=9606 GN=GNG2 PE=1 SV=2
MASNNTASIAQARKLVEQLKMEANIDRIKVSKAAADLMAYCEAHAKEDPLLTPVPASENP
FREKKFFCAIL
```

Link: https://www.uniprot.org/uniprot/P59768.fasta

Crystalised in complex with GIP agonist-bound GIPR (7DTY)

```
>sp|P63212|GBG2_BOVIN Guanine nucleotide-binding protein G(I)/G(S)/G(O) subunit
gamma-2 OS=Bos taurus OX=9913 GN=GNG2 PE=1 SV=2
MASNNTASIAQARKLVEQLKMEANIDRIKVSKAAADLMAYCEAHAKEDPLLTPVPASENP
FREKKFFCAIL
```

Initiator methionine (1), **Guanine nucleotide-binding protein G(I)/G(S)/G(O) subunit gamma-2 (2-68)**, **Propeptide (69-71)**

[Link: https://www.uniprot.org/uniprot/P63212.fasta](https://www.uniprot.org/uniprot/P63212.fasta)

```
CLUSTAL O(1.2.4) multiple sequence alignment


sp|P59768|GBG2_HUMAN      MASNNTASIAQARKLVEQLKMEANIDRIKVSKAAADLMAYCEAHAKEDPLLTPVPASENP   60
sp|P63212|GBG2_BOVIN      MASNNTASIAQARKLVEQLKMEANIDRIKVSKAAADLMAYCEAHAKEDPLLTPVPASENP   60
                          ************************************************************

sp|P59768|GBG2_HUMAN      FREKKFFCAIL   71
sp|P63212|GBG2_BOVIN      FREKKFFCAIL   71
                          ***********
```

Sequence Alignment Panel: *Sequence alignment between Human and Bovine Guanine nucleotide-binding protein G(I)/G(S)/G(O) subunit gamma-2.*

Agonist: Tirzepatide

GIP/GLP-1 Receptor Dual Agonist (Eli Lilly)

Y(*Aib*)EGTFTSDYSI(*Aib*)LDKIAQKAFVQWLIAGGPSSGAPPPS-NH_2

Tirzepatide sequence in comparison to GLP-1, GIP and Exendin-4

**Glucagon**	**HSQGTFTSDYSKYLDSRRAQDFVQWLMNT**
**GLP-1**	**HAEGTFTSDVSSYLEGQAAKEFIAWLVKGRG**
**GIP**	**YAEGTFISDYSIAMDKIHQQDFVNWLLAQKGKKNDWKHNITQ**
**Exendin-4**	**HGEGTFTSDLSKQMEEEAVRLFIEWLKNGGPSSGAPPPS**
****Tirzepatide**	**NH2-Y(*Aib*)EGTFTSDYSI(*Aib*)LDKIAQKAFVQWLIAGGPSSGAPPPS-$CONH_2$**
*****Cotadutide**	**NH2-HSQGTFTSDKSEYLDSERARDFVAWLEAGG-COOH**

Aib: α-amino-isobutyric acid; Colour Codes: GIP: Green; GLP-1: Blue; Common residue in GLP-1, GIP, Exendin-4 and Tirzepatide: Orange; Exendin-4: Purple; Common residue in GLP-1, Exendin-4 and Tirzepatide: Light blue; Mutation: Red.

USFDA Approved Dual Agonist Peptide Drug: Mounjaro (tirzepatide), 39 residues, an analogue of GIP with a C20 fatty-diacid portion attached. The fatty diacid moiety, specifically eicosanedioic acid, is linked to the side chain of the lysine residue through a linkage involving a glutamic acid and two units of (2-(2-aminoethoxy)ethoxy)acetic acid[5,8]. This arrangement results in a prolonged half-life by extended albumin binding. At positions 2 and 13, the peptide sequence of tirzepatide includes two non-coded amino acid residues, namely Aib (α-amino isobutyric acid**)[20].

Tirzepatide peptide modelled with neutral N and amidated C terminals (NNEU and CT2). Tirzepatide has a C20 diacid-γ-Glu(AEEA)2-fatty acid side chain bound to the Lysine (K) in position 20. For comparison purposes, we have simulated both reference peptides (cotadutide and tirzepatide) **without their respective fatty acid modification.**

***Cotadutide peptide modelled with neutral N and C terminals (NNEU and CNEU. Cotadutide is a chimeric peptide employing important residues from both glucagon and GLP-1 into its sequence, with a palmitoyl fatty acid attachment on Lys10 to prolong the circulating half-life[1].

Figures

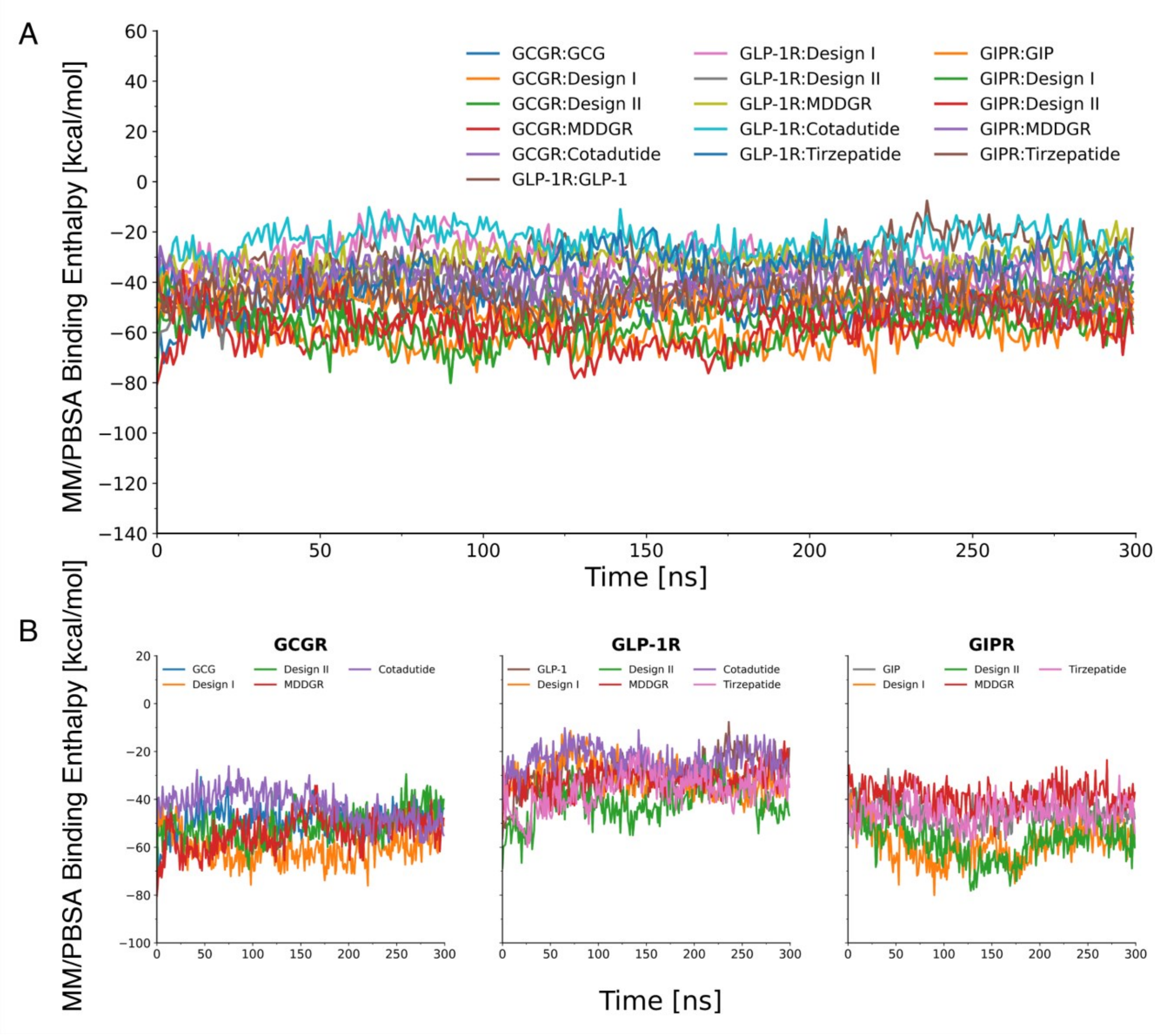

Fig. S1. The timelines of calculated effective binding enthalpies for agonist-receptor complexes. (A) Averaged effective binding enthalpy profiles over 0.3 μs are shown for all sixteen agonist-receptor complexes. Each line represents the mean of three independent 0.3 μs simulations per complex. (B) The same averaged effective binding enthalpy timelines are presented separately by receptor: GCGR, GLP-1R and GIPR. This representation highlights receptor-specific binding profiles and relative binding strengths across endogenous ligands, peptide designs I and II, cotadutide, tirzepatide and MDD_{GR}. Effective binding enthalpies were calculated using gmx_MMPBSA.

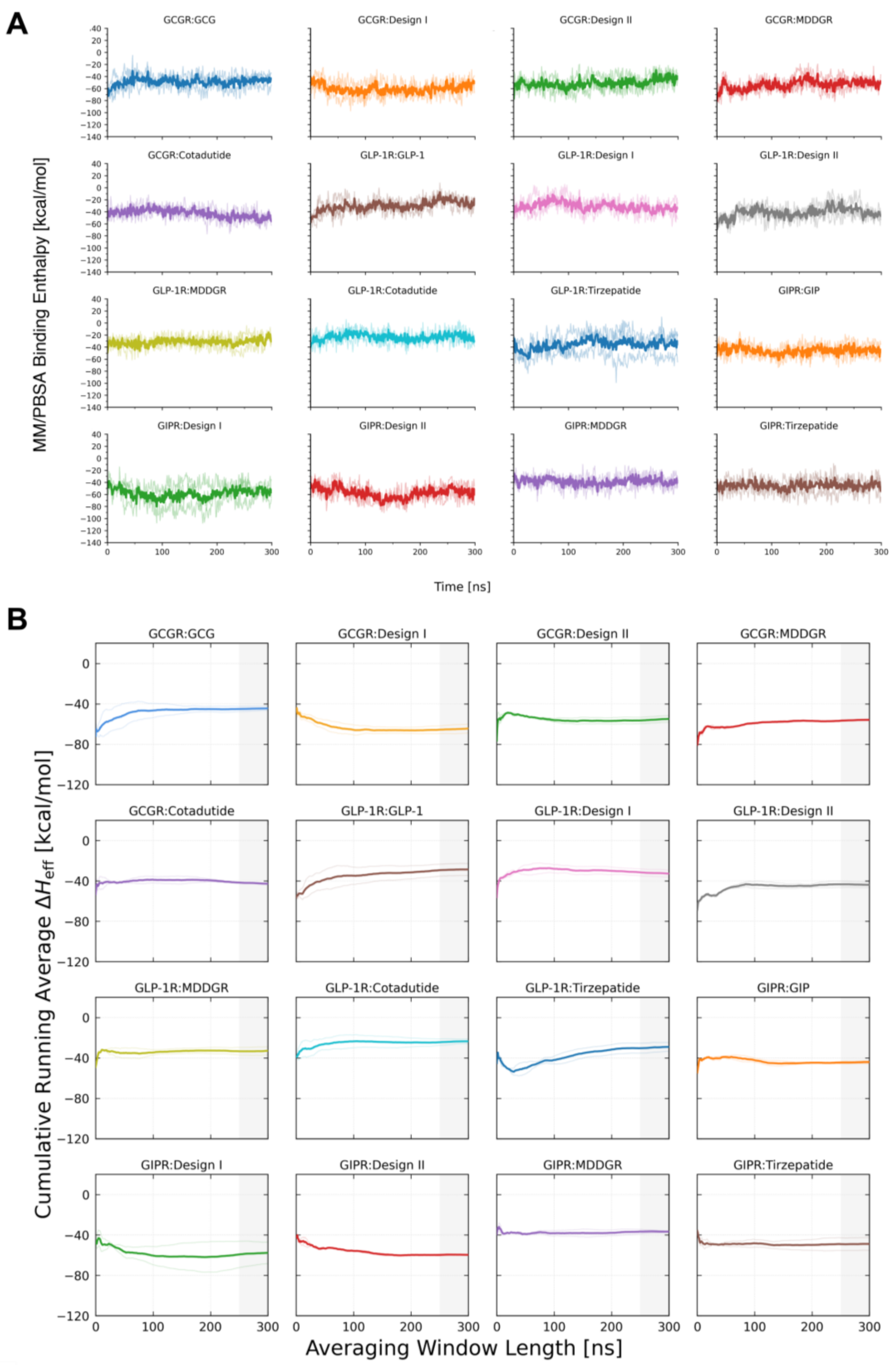

Fig. S2. (A) Timelines of effective binding enthalpies for replicate simulations. Effective binding enthalpies over 0.3 µs are shown for all sixteen receptor-agonist complexes. Each panel presents data from three replicate 0.3 µs simulations (two independent repeats and the original run), with individual replicates span shown in lighter tones. The averaged timeline across replicates is overlaid, highlighting the consistency and reproducibility of effective binding enthalpy profiles across independent simulations. Complexes include the endogenous ligands (glucagon, GLP-1 and GIP), cotadutide, tirzepatide, MDD_{GR}, and peptide designs I and II in complex with their respective receptors (GCGR, GLP-1R and GIPR). **(B)** Cumulative running-average profiles of the calculated effective binding

enthalpies (ΔH_{eff}). ΔH_{eff} are plotted as a function of cumulative averaging window length across the 0.3 µs production trajectories for all sixteen complexes. Thin lines correspond to individual replicate simulations, with the bold line denoting the replicate average. The stabilisation of the running averages in the latter part of the trajectories supports using the final 50 ns (grey-shaded region) for comparative MM/PBSA analysis. The shaded grey rectangles highlight the final 50 ns (250-300 ns) production window utilised for average sampling metrics.

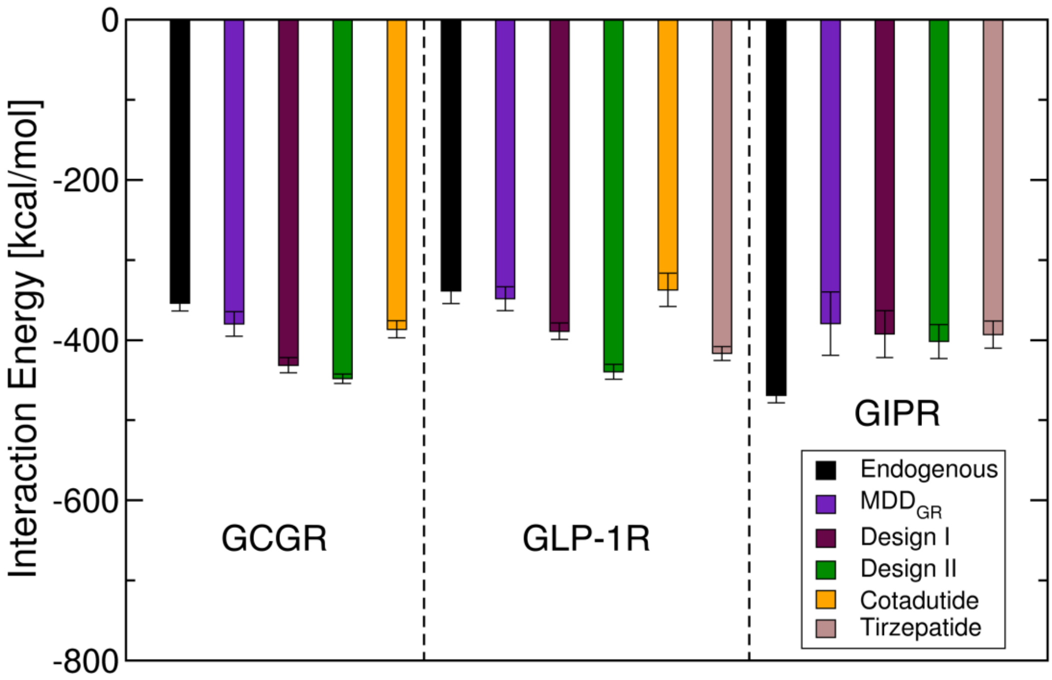

Fig. S3. Comparison of interaction energy (in kcal/mol) for agonist peptides in complex with GCGR, GLP-1R and GIPR. Interaction energies were calculated as the mean values over the final 50 ns of replicate simulations for each receptor-agonist complex. Complexes were ranked based on interaction strength (more negative values indicating stronger interactions). For GCGR: Design II > Design I > Cotadutide > MDD$_{GR}$ > Endogenous ligand (glucagon), for GLP-1R: Design II > Tirzepatide > Design I > MDD$_{GR}$ > Endogenous ligand (GLP-1) > Cotadutide, and for GIPR: Endogenous ligand (GIP) > Design II > Tirzepatide > Design I > MDD$_{GR}$. The interaction energies represent averages across three replicate last 50 ns simulations per complex. Error bars denote the standard error of the mean.

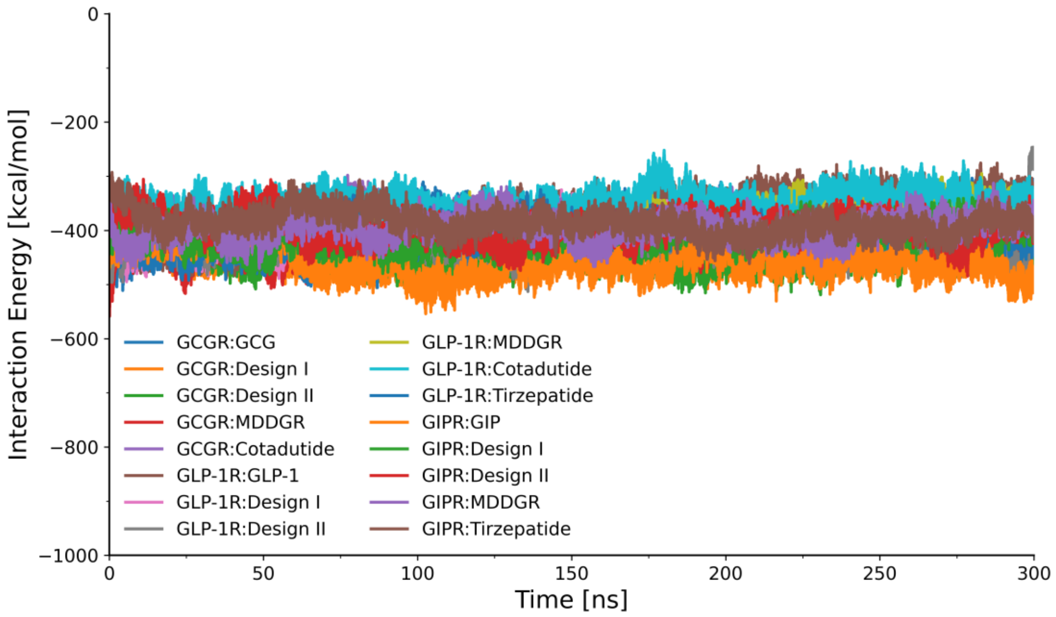

Fig. S4. The timelines of calculated interaction energies for agonist/receptor complexes.

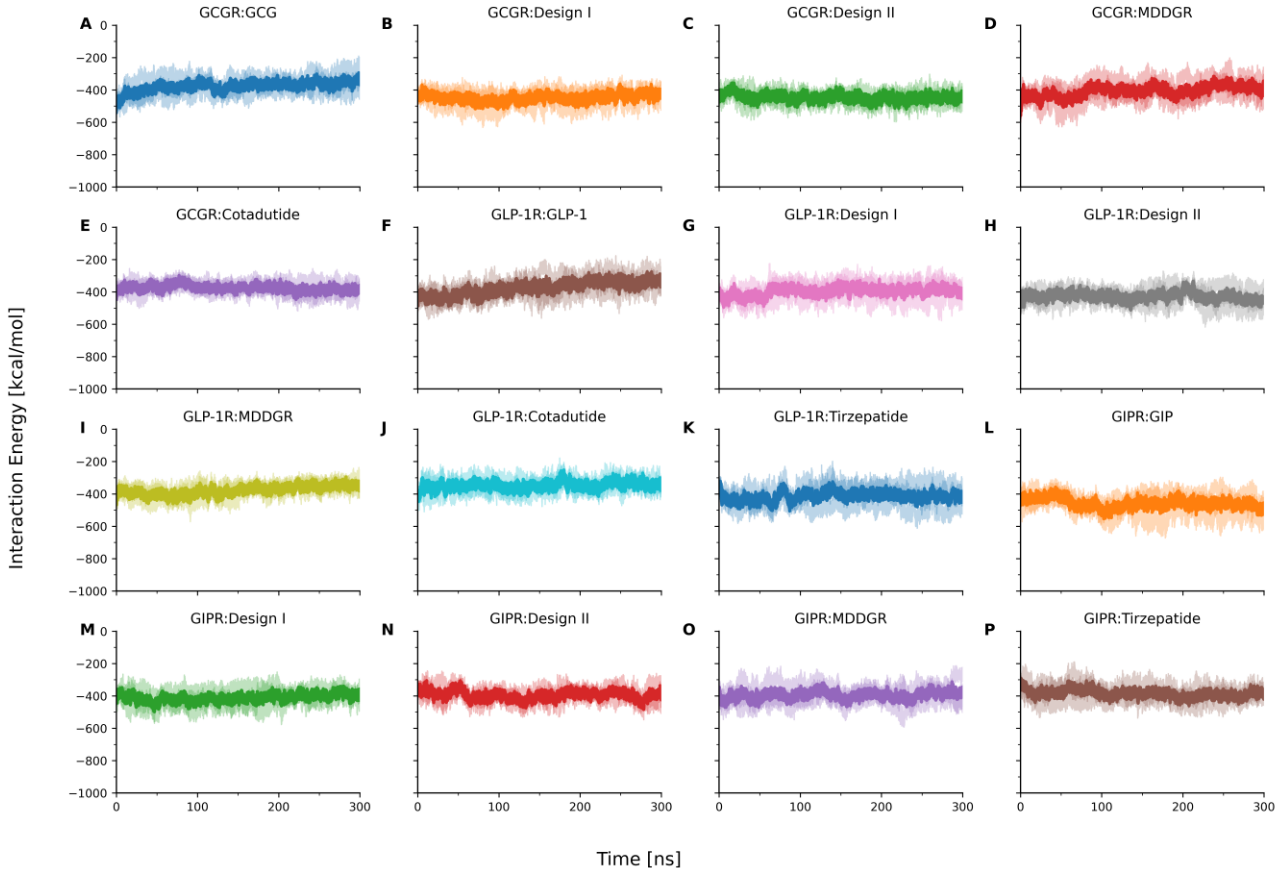

Fig. S5. Timelines of interaction energies for replicate simulations. Interaction energies over 0.3 µs are shown for all sixteen receptor-agonist complexes. Each panel presents data from three replicate 0.3 µs simulations (two independent repeats and the original run), with individual replicates span shown in lighter tones. The averaged timeline across replicates is overlaid, highlighting the consistency and reproducibility of interaction energy profiles across independent simulations. Complexes include the endogenous ligands

(glucagon, GLP-1 and GIP), cotadutide, tirzepatide, MDD_{GR}, and peptide designs I and II in complex with their respective receptors (GCGR, GLP-1R and GIPR).

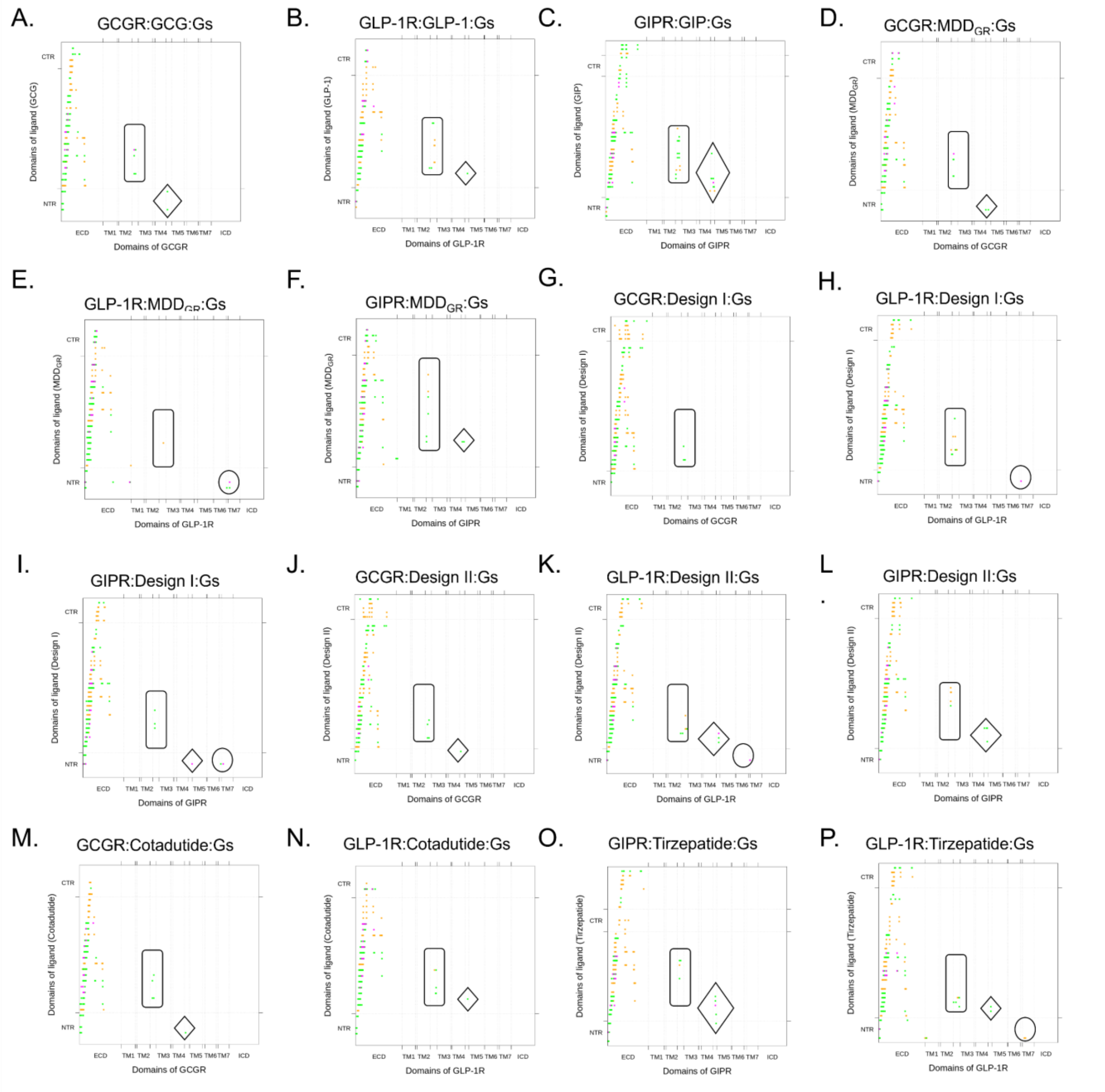

Fig. S6. Interaction maps of different agonists and receptor complexes computed from the last 100 ns of simulations using the CONAN tool[21]. Specific interaction types in the maps are coloured: orange - hydrophobic, green - hydrogen bond, and pink - salt bridge. The square shape (▢) highlights the interactions between the peptides and ECL1 connecting the helix TM2 and TM3; the diamond shape (◊) highlights the interactions between the peptides and ECL2 connecting the helix TM4 and The M5; circle (◯)) highlights the interactions between the peptides and ECL3 connecting the helix TM6 and TM7, including the agonist interactions with residues in extracellular (ECL3) proximity of TM7.

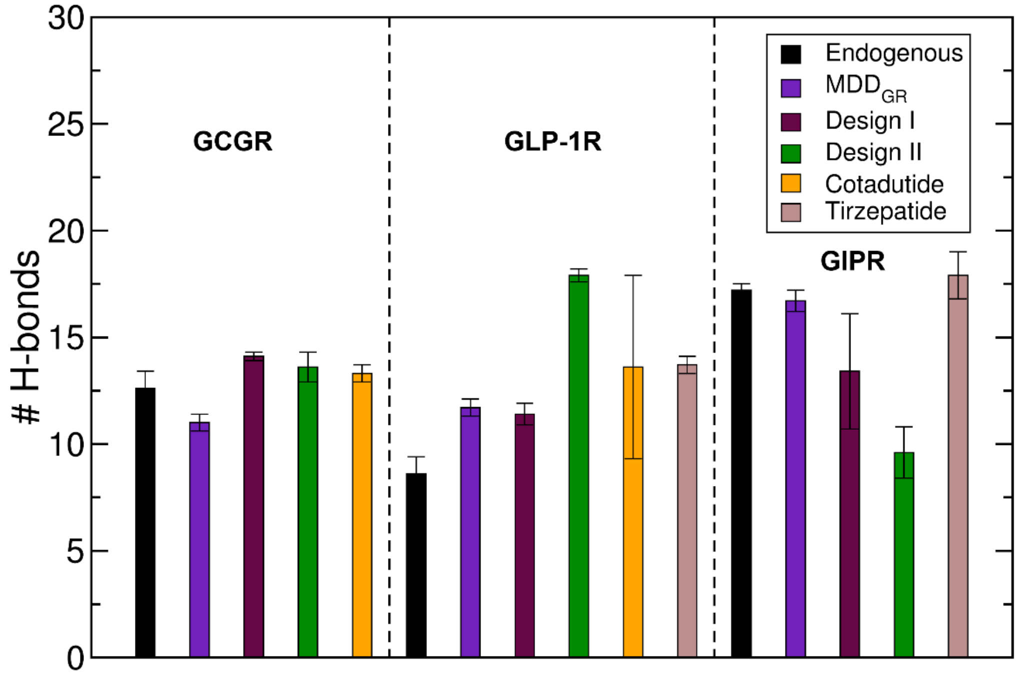

Fig. S7. Agonist-receptor intermolecular hydrogen bonds (H-bonds) from MD simulations. The average number of hydrogen bonds during the last 50 ns of simulations of peptides with GCGR, GLP-1R and GIPR. A minimum donor-acceptor distance of 3.5 Å and a hydrogen-donor-acceptor angle of 30° was used for the H-bond analyses.

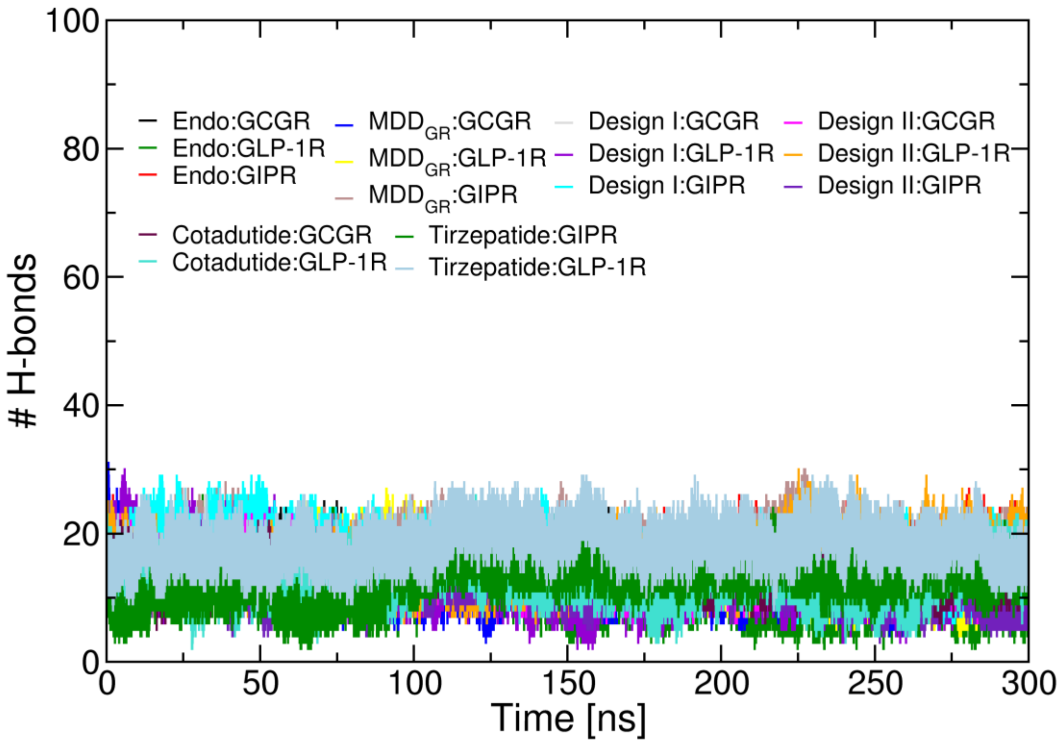

Fig. S8. The timelines of the calculated number of H-bonds for agonist/receptor complexes.

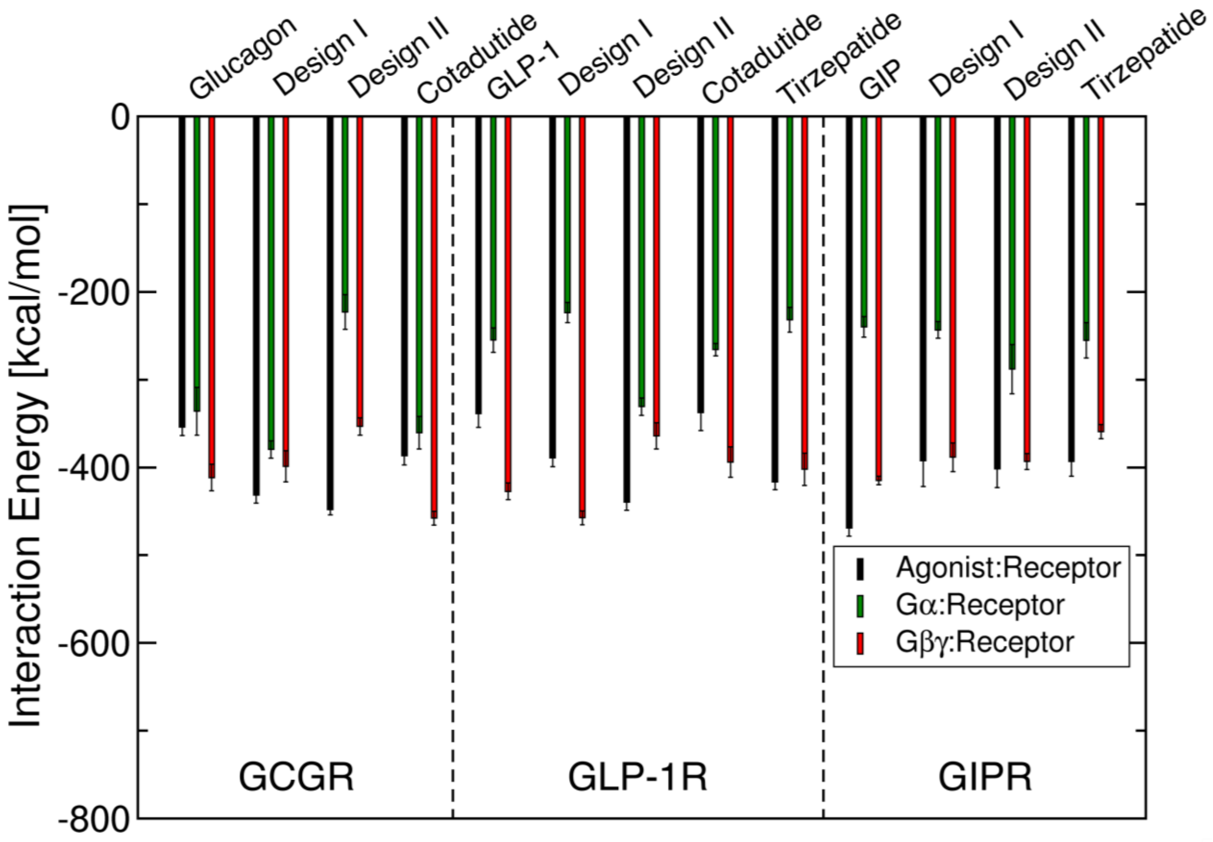

Fig. S9. Calculated interaction energies between Agonist:Receptor, Gα:Receptor and Gβγ:Receptor groups using GROMACS gmx energy tool. Data represent averages from replicate simulations. Error bars indicate the standard error of the mean (SEM).

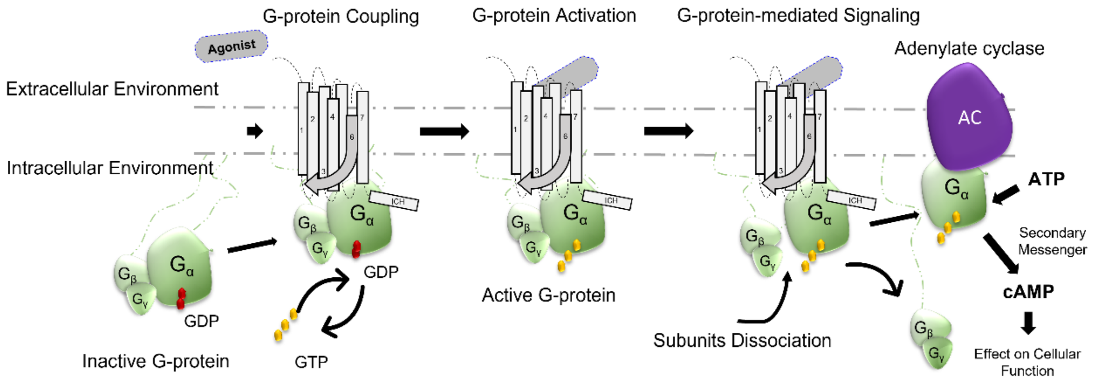

Fig. S10. Schematic of Gsα subunit activation upon GPCR stimulation.

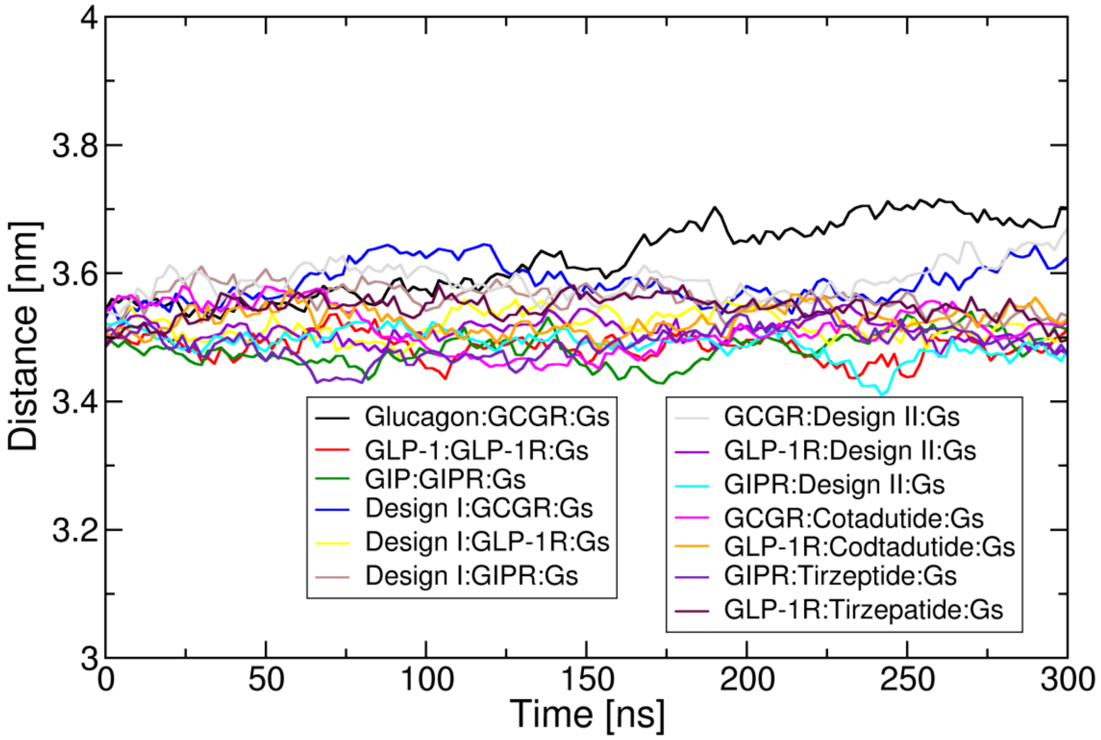

Fig. S11. Calculated COM-COM distances between Gα and Gβγ subunits using the GROMACS gmx_distance tool. Abbreviation: COM, centre-of-mass.

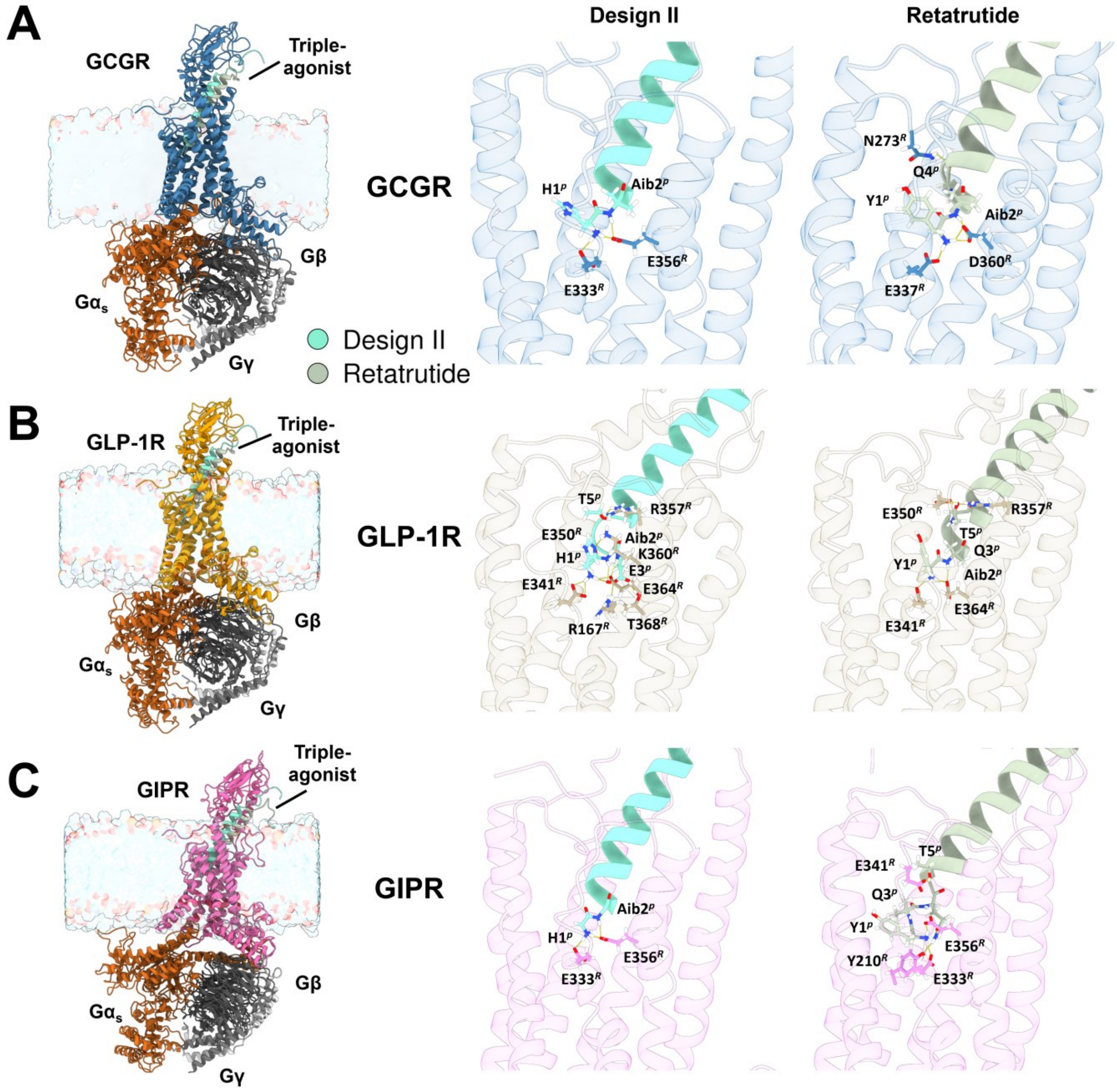

Fig. S12. Structural comparison and binding pocket alignment of Design II and retatrutide across Class B1 GPCR complexes. Full structural models embedded in a lipid bilayer with coupled heterotrimeric G-protein subunits (Gα, Gβ, Gγ) are shown for (A) GCGR, (B) GLP-1R and (C) GIPR. Zoomed-in panels illustrate the comparative local binding networks of N-terminal (residue indices 1 to 5) Design II (cyan helix) and retatrutide (green helix) captured from the final frame of the 300 ns trajectories. Dashed lines denote key hydrogen bonding interactions (within 3.5 Å) between specific peptide residues (superscript ***p***) and receptor pocket residues (superscript ***R***). Alignments demonstrate that Design II recapitulates key helical anchoring networks observed in the clinical benchmark and maintains the newly introduced, highly complementary exodomain contacts across all three interfaces.

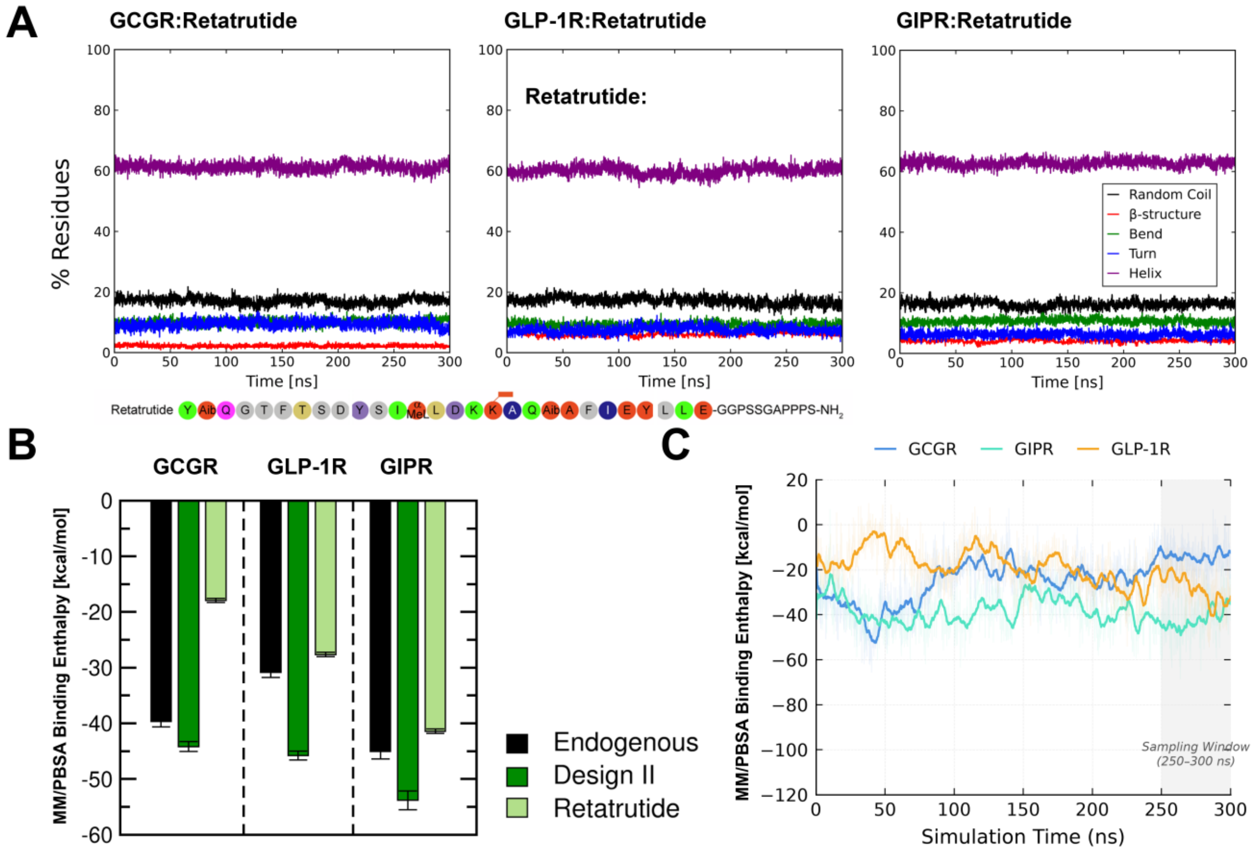

Fig. S13. Secondary structure stability and thermodynamic binding enthalpies of the triple-agonist benchmark, retatrutide, in complex with its respective receptors. (A) Secondary-structure analyses of GCGR, GLP-1R, and GIPR bound to retatrutide over the 300 ns trajectory, illustrating persistent helical backbone conservation. (B) Computed MM/PBSA binding enthalpies across the three target receptors, contrasting endogenous ligands against Design II and retatrutide. (C) Dynamic effective binding enthalpy (ΔH_{eff}) timelines highlighting trajectory stability. Mean calculated from the final 50 ns (250-300 ns) equilibrium sampling window, denoted by the shaded area in the plot.

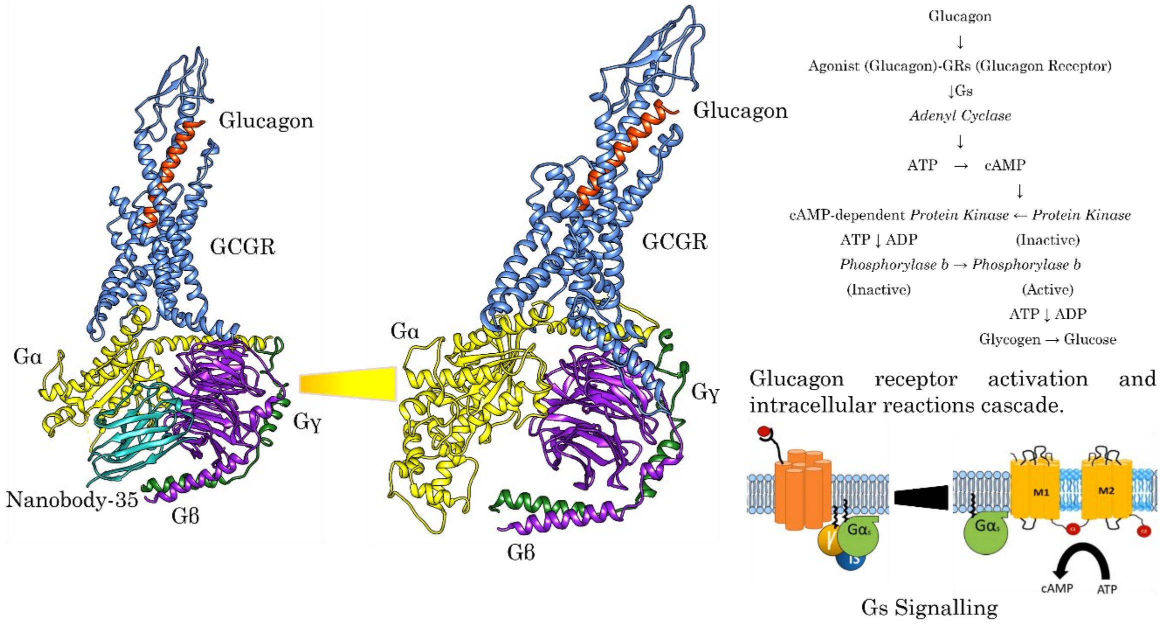

Fig. S14. Structure of the parent crystal structure of the representative receptor (glucagon receptor) from Class B1 GPCR family, the modelled GCG-GCGR-Gs complex, and the glucagon receptor signalling pathway.

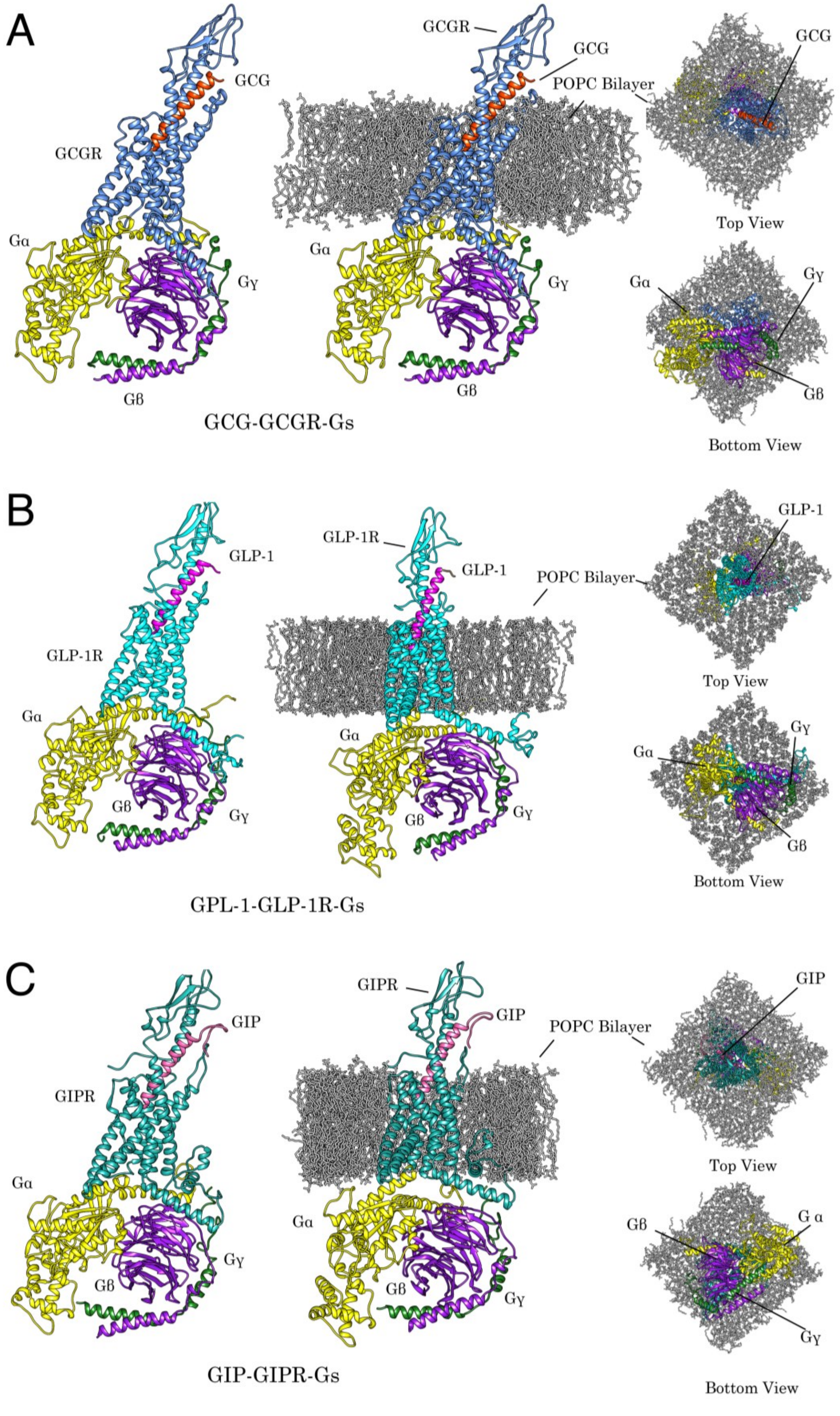

Fig. S15. Models of endogenous agonist-bound GCG, GIP, and GLP-1 receptors complexed with heterotrimeric Gs protein (Gα, Gβ, and Gγ subunits). Membrane-bound GCG–GCGR–Gs (A), GLP-1–GLP-1R–Gs (B) and GIP–GIPR–Gs (C) complexes are shown, with encasing water omitted for clarity.

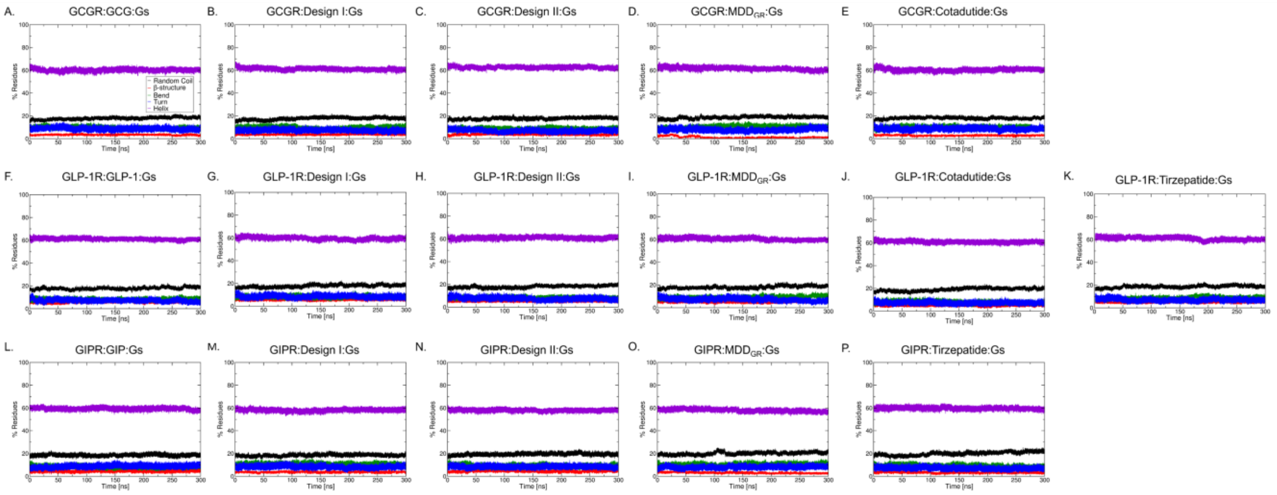

Fig. S16. Structural dynamics of class B1 GPCRs. Secondary structural changes in the class B1 GPCRs throughout simulations of peptide agonist:receptor:Gs complex: (A) glucagon (GCG) agonist-bound GCGR, (B) Design I triple-agonist-bound GCGR, (C) Design II triple-agonist-bound GCGR, (D) MDD$_{GR}$ co-agonist agonist-bound GCGR, (E) Cotadutide-bound GCGR, (F) GLP-1 agonist-bound GLP-1R, (G) Design I triple-agonist-bound GLP-1R, (H) Design II triple-agonist-bound GLP-1R, (I) MDD$_{GR}$ co-agonist agonist-bound GLP-1R, (J) Cotadutide-bound GLP-1R, (K) Tirzepatide-bound GLP-1R (L) GIP agonist-bound GIPR, (M) Design I triple-agonist-bound GIPR, (N) Design II triple-agonist-bound GIPR, (O) MDD$_{GR}$ co-agonist agonist-bound GIPR and (P) Tirzepatide-bound GIPR.

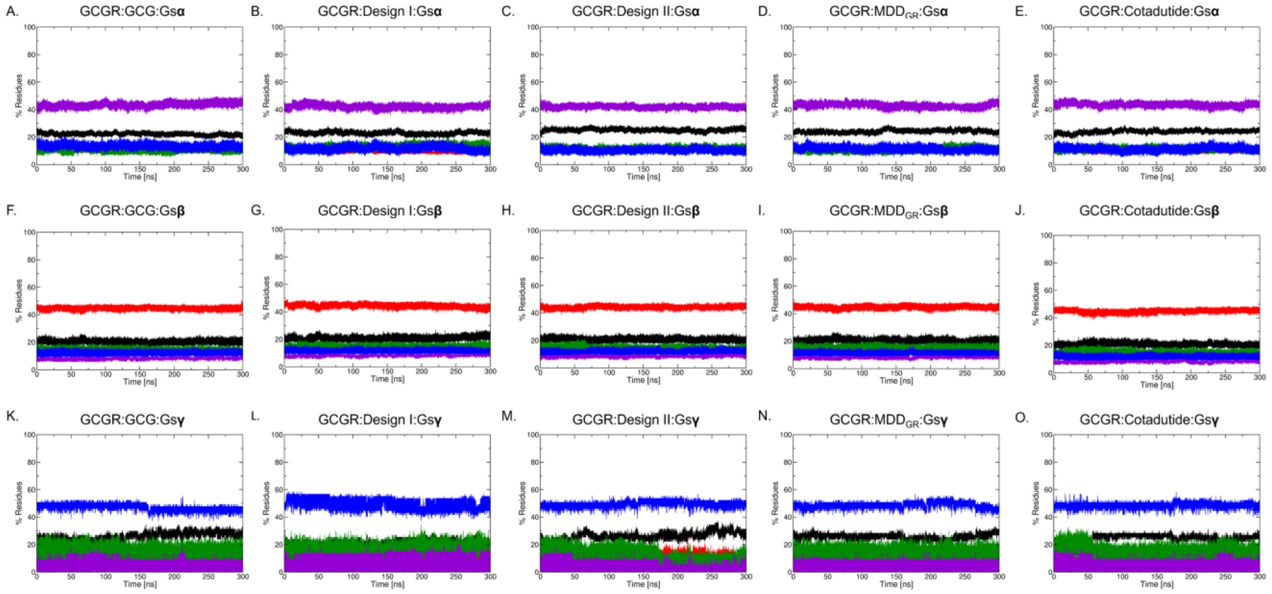

Fig. S17. Structural dynamics of the simulated Gs protein due to the influence of extracellular ligand binding to the glucagon receptor (GCGR). Secondary structural changes in the heterotrimeric Gs subunits throughout simulations of peptide agonist:GGCR:Gs complex when complexed with: (A-E) Gsα, (F-J) Gsβ and (K-O) Gsγ.

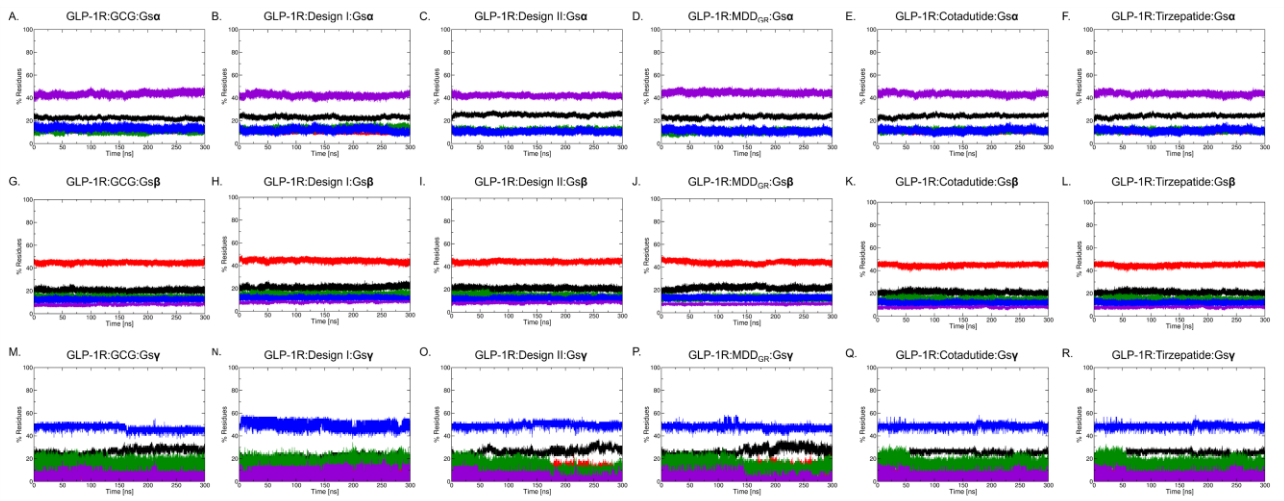

Fig. S18. Structural dynamics of simulated Gs protein due to the influence of extracellular ligand binding to GLP-1R. Secondary structural changes in the heterotrimeric Gs subunits throughout simulations of peptide agonist:GLP-1R:Gs complex when complexed with: (A-E) Gsα, (F-J) Gsβ and (K-O) Gsγ.

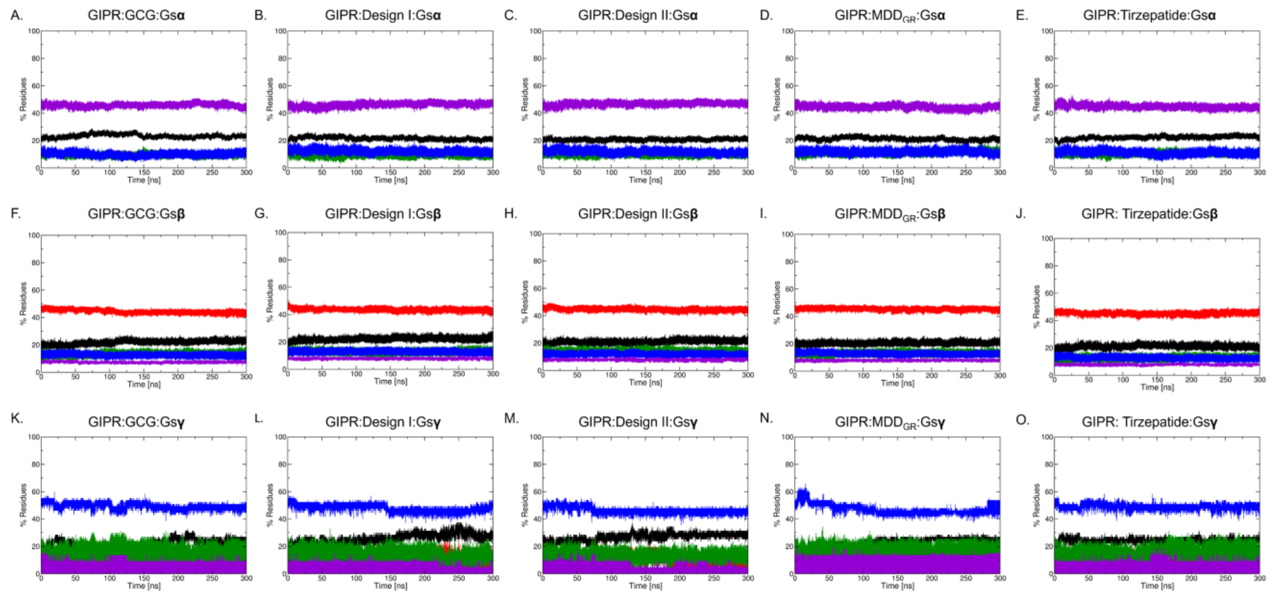

Fig. S19. Structural dynamics of the simulated Gs protein due to the influence of extracellular ligand binding to GIPR. Secondary structural changes in the heterotrimeric Gs subunits throughout simulations of peptide agonist:GIPR:Gs complex when complexed with: (A-E) Gsα, (F-J) Gsβ and (K-O) Gsγ.

Replicates of Simulations: Secondary Structure Analyses

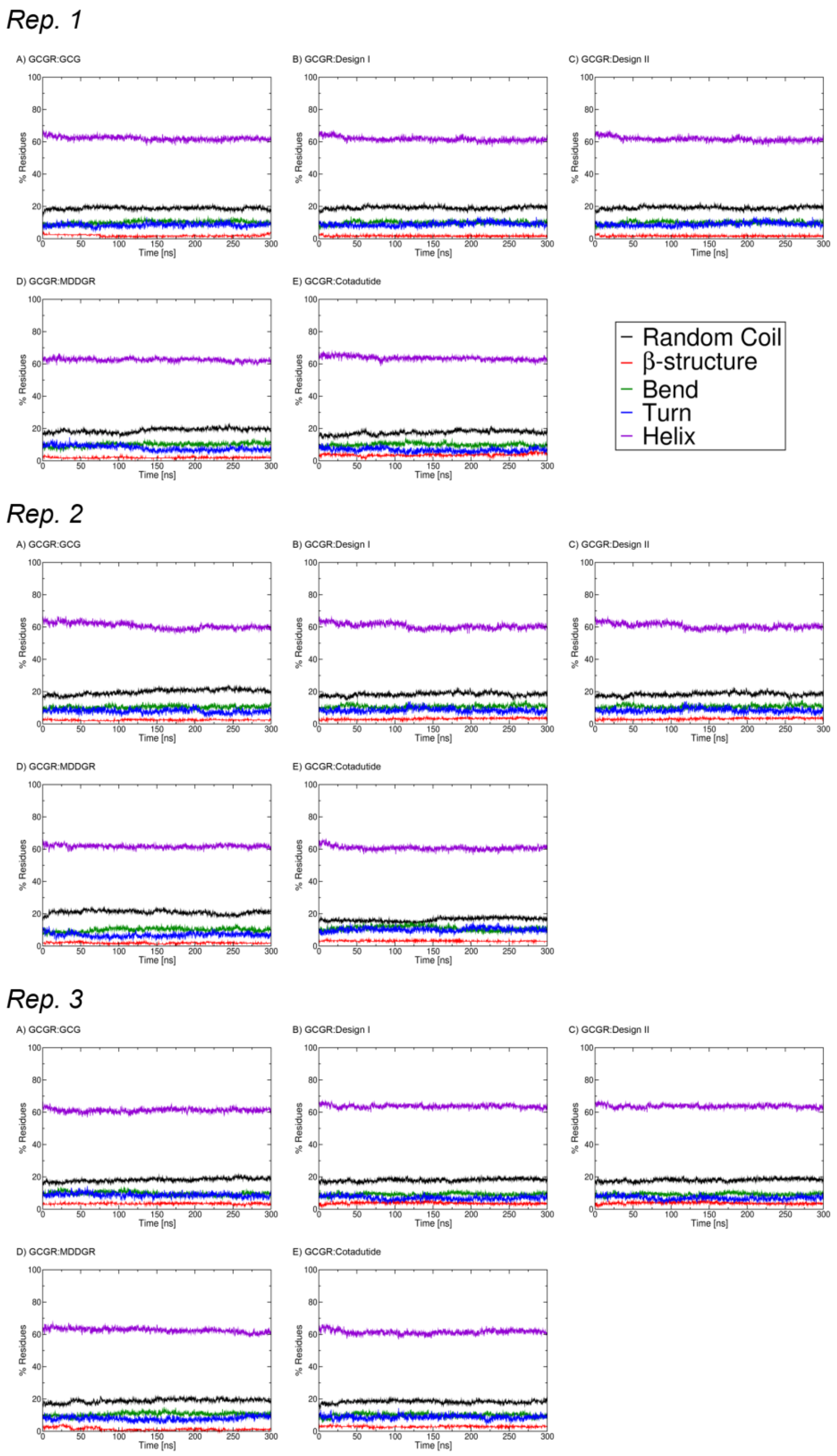

Fig. S20. Secondary structure analyses of the **GCGR receptor** bound to various ligands, shown for three independent simulation replicates (rep1, rep2, rep3). Each panel includes subpanels labelled A, B, C, *etc.*, corresponding to distinct ligand designs as specified. The images illustrate consistent secondary structure patterns across replicates and ligand variations.

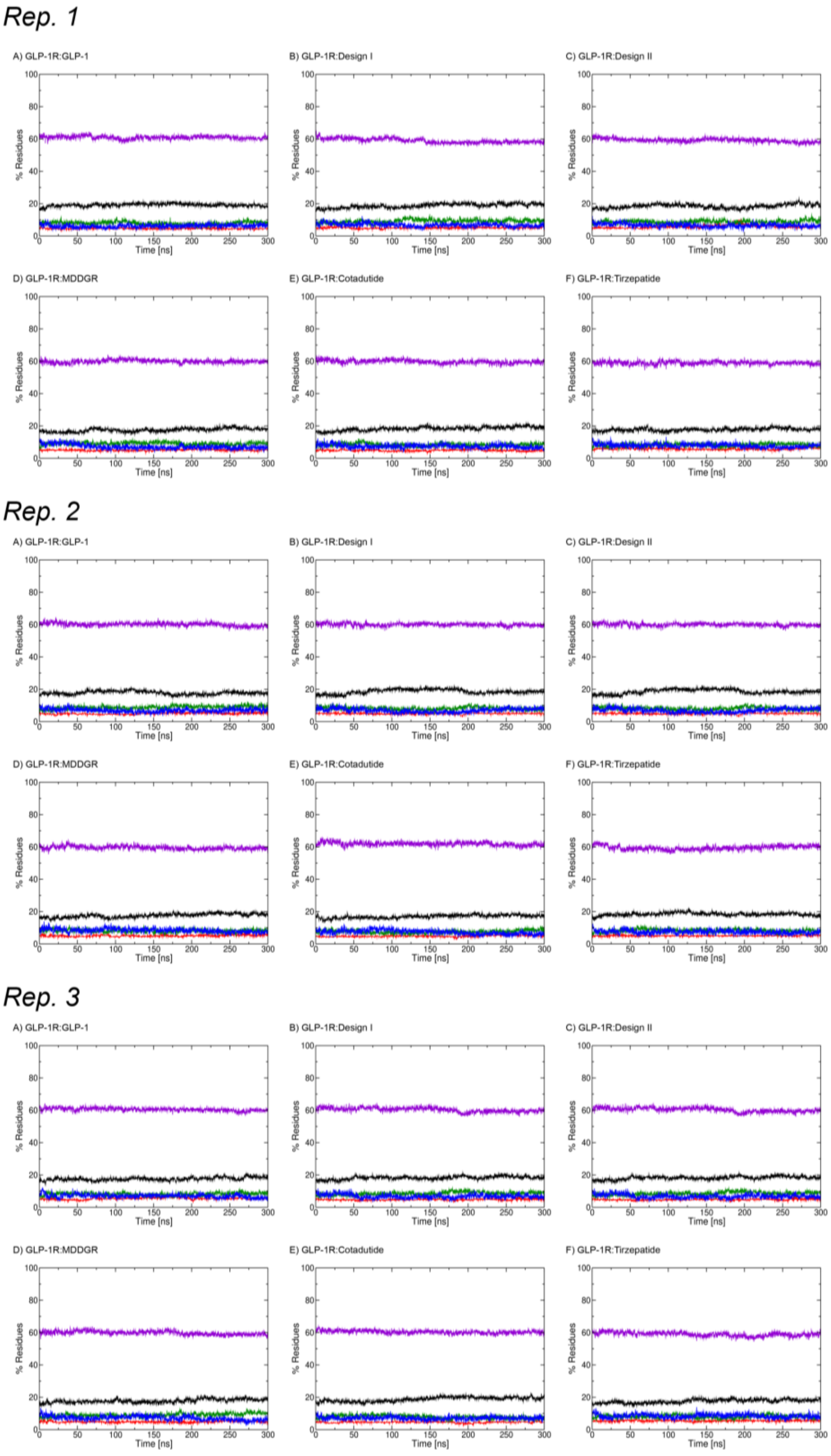

Fig. S21. Secondary structure analyses of the **GLP-1R receptor** with multiple ligand variants, shown for three independent simulation replicates (rep1, rep2, rep3). Subpanels, labelled A, B, C, *etc.*, highlight differences in secondary structure induced by ligand design. These replicate images demonstrate the reproducibility of structural features.

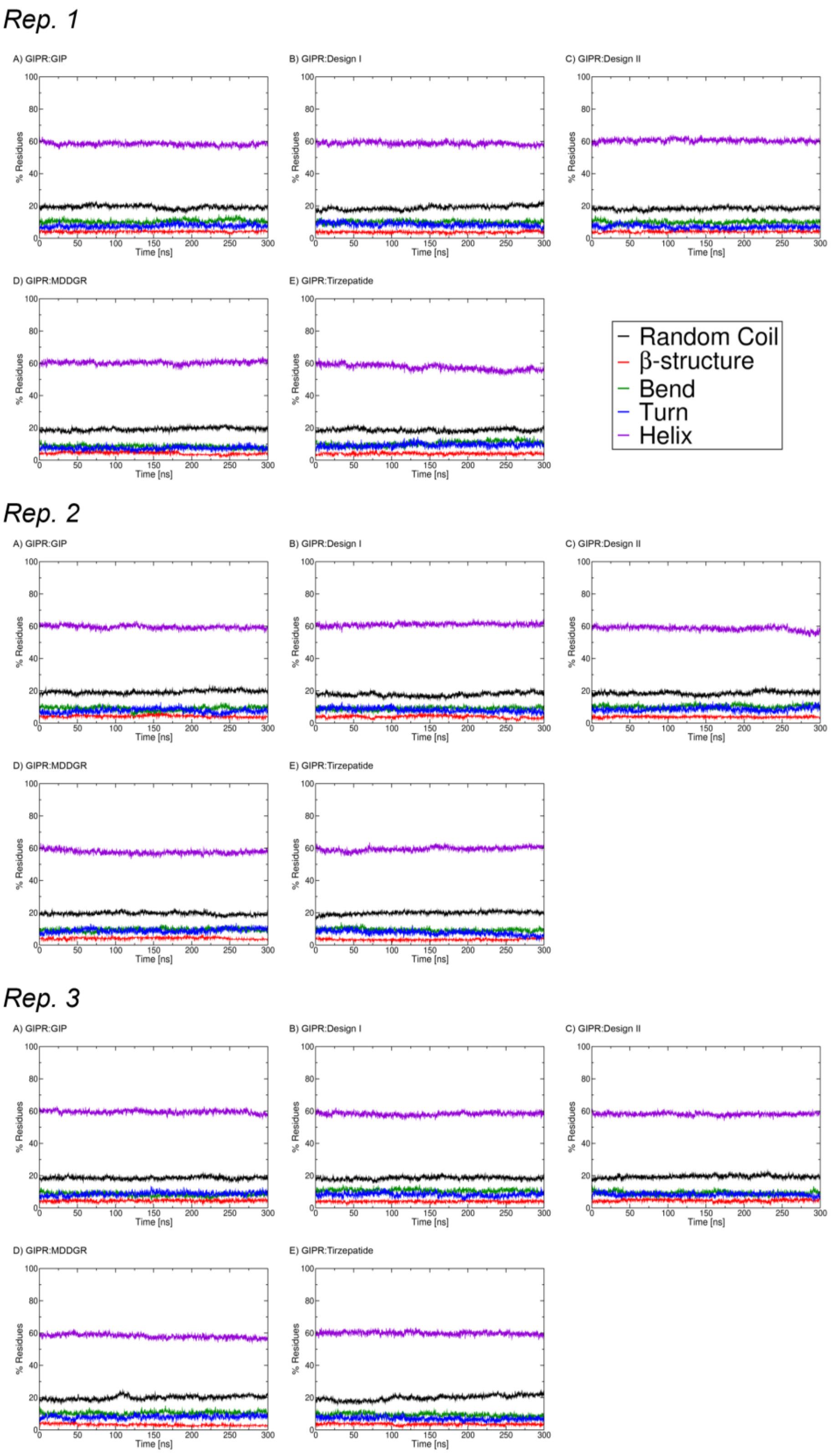

Fig. S22. Secondary structure analyses of the **GIPR receptor** bound to different ligands, shown for three independent simulation replicates (rep1, rep2, rep3). The replicates collectively showcase the robustness of observed secondary structure motifs.

Supplementary references

1. Parker VER, Hoang T, Schlichthaar H, Gibb FW, Wenzel B, Posch MG, Rose L, Chang YT, Petrone M, Hansen L, Ambery P, Jermutus L, Heerspink HJL, McCrimmon RJ (2022) Diabetes Obes Metab 24(7):1360
2. Boland ML, Laker RC, Mather K, Nawrocki A, Oldham S, Boland BB, Lewis H, Conway J, Naylor J, Guionaud S, Feigh M, Veidal SS, Lantier L, McGuinness OP, Grimsby J, Rondinone CM, Jermutus L, Larsen MR, Trevaskis JL, Rhodes CJ (2020) Nature Metabolism 2(5):413
3. Vishnoi S, Bhattacharya S, Walsh EM, Okoh GI, Thompson D (2023) Journal of Chemical Information and Modeling 63(15):4934
4. Nauck MA, D'Alessio DA (2022) Cardiovasc Diabetol 21(1):169
5. Chavda VP, Ajabiya J, Teli D, Bojarska J, Apostolopoulos V (2022) Molecules 27(13):4315
6. Wang L (2022) Drug Des Devel Ther 16:1547
7. Mishra R, Raj R, Elshimy G, Zapata I, Kannan L, Majety P, Edem D, Correa R (2023) Journal of the Endocrine Society 7(4)
8. Wilson JM, Nikooienejad A, Robins DA, Roell WC, Riesmeyer JS, Haupt A, Duffin KL, Taskinen M-R, Ruotolo G (2020) Diabetes, Obesity and Metabolism 22(12):2451
9. Baxa MC, Haddadian EJ, Jha AK, Freed KF, Sosnick TR (2012) Journal of the American Chemical Society 134(38):15929
10. Doig AJ, Sternberg MJ (1995) Protein Sci 4(11):2247
11. Hess B (2002) The Journal of Chemical Physics 116(1):209
12. Kabsch W, Sander C (1983) Biopolymers 22(12):2577
13. Bhat VK, Kerr BD, Vasu S, Flatt PR, Gault VA (2013) Diabetologia 56(6):1417
14. Alfaris N, Waldrop S, Johnson V, Boaventura B, Kendrick K, Stanford FC (2024) eClinicalMedicine 75
15. Wen J, Nadora D, Truong A, Bernstein E, How-Volkman C, Razick A, Razick D, Karabala M, Frezza E (2025) Nutrition, Metabolism and Cardiovascular Diseases 35(12):104213
16. Urva S, Coskun T, Loh MT, Du Y, Thomas MK, Gurbuz S, Haupt A, Benson CT, Hernandez-Illas M, D’Alessio DA, Milicevic Z (2022) The Lancet 400(10366):1869
17. KIM JK, LEE JS, CHOI J, JUNG SY, LEE SH, CHOI IY, KIM SJ (2018) Diabetes 67(Supplement 1):77
18. Bossart M, Wagner M, Elvert R, Evers A, Hübschle T, Kloeckener T, Lorenz K, Moessinger C, Eriksson O, Velikyan I, Pierrou S, Johansson L, Dietert G, Dietz-Baum Y, Kissner T, Nowotny I, Einig C, Jan C, Rharbaoui F, Gassenhuber J, Prochnow HP, Agueusop I, Porksen N, Smith WB, Nitsche A, Konkar A (2022) Cell Metab 34(1):59
19. Gasteiger E, Hoogland C, Gattiker A, Duvaud Se, Wilkins MR, Appel RD, Bairoch A (2005) Protein identification and analysis tools on the ExPASy server. Springer,
20. Zhao F, Zhou Q, Cong Z, Hang K, Zou X, Zhang C, Chen Y, Dai A, Liang A, Ming Q, Wang M, Chen L-N, Xu P, Chang R, Feng W, Xia T, Zhang Y, Wu B, Yang D, Zhao L, Xu HE, Wang M-W (2022) Nature Communications 13(1):1057
21. Mercadante D, Gräter F, Daday C (2018) Biophysical Journal 114(6):1267
